# Rapid Generation of Transition-State Conformer Ensembles via Constrained Distance Geometry

**DOI:** 10.1021/acs.jcim.5c02794

**Published:** 2026-02-12

**Authors:** Stefan P. Schmid, Henrik Seng, Thibault Kläy, Kjell Jorner

**Affiliations:** † Institute of Chemical and Bioengineering, Department of Chemistry and Applied Biosciences, 27219 ETH Zurich, Zurich CH-8093, Switzerland; ‡ NCCR Catalysis, Zurich CH-8093, Switzerland

## Abstract

Consideration of transition-state (TS) conformer ensembles is required to accurately model a reaction, and thus plays a key role in computational catalyst design. While CREST and GOAT are established methods for TS conformer ensemble generation, the associated computational cost remains a major bottleneck in computational chemistry pipelines, including for the generation of large machine learning data sets for catalyst design. To this end, we present racer^TS^ (**RA**pid **C**onformer **E**nsembles with **RDK**it for **T**ransition **S**tates), a method for efficient TS conformer ensemble generation. In this work, we describe the algorithm behind racer^TS^, which is based on constrained distance geometry. To benchmark the performance of racer^TS^ against CREST and GOAT, we created conformer ensembles for transition states of 20 diverse reactions. To assess the utility of each conformer generator in computational chemistry workflows, we optimize selected low-energy and diverse conformers at the DFT level. We use the generated conformer ensembles and the results of this pipeline to assess conformer generators according to the following metrics: computational cost, exhaustiveness, validity, and accuracy in low-energy regions. Considering the generated ensembles, we find that racer^TS^ covers the conformer space similarly to CREST and slightly less comprehensively than GOAT, while the validity of the DFT-optimized TSs is better and the accuracy in the low-energy region is sufficient for computational chemistry applications (median error of 0.17 kcal/mol). Remarkably, racer^TS^ achieves these results with a significant reduction in required wall-time. Our results demonstrate that racer^TS^ is a highly efficient TS conformer ensemble generator, allowing for rapid TS conformer sampling in computational chemistry pipelines. Additionally, racer^TS^ paves the way to create meaningful TS data sets to advance machine learning methods for the discovery of novel and sustainable catalysts.

## Graphical abstract

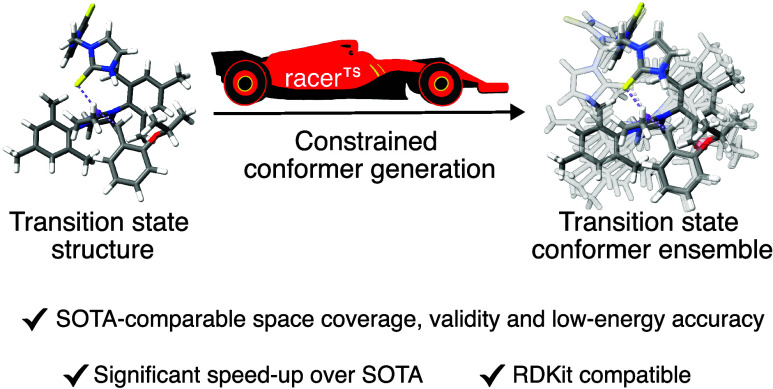

## Introduction

The development of catalysts for novel and more sustainable chemical reactions is a tedious process that can be accelerated via computations.^1,2^ Multiple reaction properties, such as the activation energy (*E* ^‡^) or the selectivity of a reaction, are governed by transition states (TSs), and their structures and energies should be precisely modeled to obtain accurate predictions. A prerequisite for an accurate modeling is the computation of conformer ensembles of compounds of interest.^3,4^ Showcasing the importance of conformer ensembles in TS modeling, Laplaza *et al.* recently demonstrated how different treatments of conformer ensembles for transition states can even reverse the predicted reaction selectivity.^5^ Conformer ensembles of transition states are also increasingly considered when predicting reaction properties with machine learning models.^6,7^ For instance, Zhu *et al.* ^7^ note that accounting for information in TS conformer ensembles to predict the enantiomeric excess in Ru-catalyzed asymmetric hydrogenations of enamides^8^ can lower prediction error by 20% compared to only considering the lowest-energy conformer. These investigations clearly underline that acquiring information from TS conformer ensembles is instrumental in achieving more accurate computational predictions, which are required for successful integration of computational tools into the catalyst development pipeline to discover sustainable chemical processes.

When computing ground-state structures, conformer ensembles are increasingly included in widely available data sets used for machine learning.^9–13^ For computing conformer ensembles of ground-state structures, multiple programs are openly available, such as CREST,^4,14,15^ Global Optimization Algorithm (GOAT),^16^ RDKit,^17–19^ Balloon,^20^ machine learning (ML) methods,^21–30^ and also commercial softwares.^31–38^ Each of these programs offers a different trade-off in terms of accuracy, conformer space coverage, and computational cost and have been extensively benchmarked in the past years.^9,39–41^ Of these programs, CREST is commonly employed when accurate conformer ensembles are required, but computational cost is a smaller problem due to its approach grounded in physics-based simulations using metadynamics (see below). In many cases, this metadynamics-based approach leads to conformer ensemble generation being the bottleneck within a computational chemistry pipeline.^11,42–44^ A faster alternative to physics-based simulations is RDKit’s ETKDG method,^17^ which has also been used to construct large data sets for machine learning purposes.^29^ Based on the distance geometry algorithm, the ETKDG method samples interatomic distances randomly from a distance bounds matrix, providing a significant reduction in computational cost.^11^ In addition to random sampling of interatomic distances, conformers are subsequently refined by using additional chemical knowledge and experimental data within the ETKDG algorithm. Conformers sampled with the ETKDG algorithm are commonly postprocessed via geometry optimization using a fast force-field method.^18^

In contrast to ground states, fewer conformer generation methods are available for transition states. Recently, ML has been applied to the task of transition-state generation,^45–51^ including the TSDiff^49^ model by Kim and co-workers. By sampling the model repeatedly for the same reactants and products, multiple TS conformers can be generated. Other models include those by Heid and co-workers^51^ and the React-OT model of Duan, Liu, Du, and co-workers that can generate TS conformer ensembles starting from different pairs of reactant and product conformers.^50^ While these advances are promising, all ML methods are inherently limited by their training data. Currently, most TS structure generation models are trained on data sets with only up to 11 heavy atoms,^46,52–54^ limiting their applicability domain. Most of the data sets also contain only organic structures, with, to the best of the authors’ knowledge, only Kraka and co-workers considering reactions involving transition metals.^47^ Quantum chemistry- and cheminformatics-based conformer generators currently have a broader applicability domain and will have a key role in generating more diverse and accurate training data for generative models. The lack of such methods can be explained by the fact that defining a valid molecular topology with appropriate atom types remains challenging for transition states, an area of active research for so-called transition-state force fields.^55^ In particular, the *reaction center*, *i.e.*, the atoms and bonds that are involved in the bond breakages and formations in a reaction, presents a major challenge in conformer generation. As the interatomic distances and angles of the TSs are typically not observed in ground-state structures, force fields show a low accuracy for these bonds/angles. They are further not integrated into knowledge-based algorithms, and ML generators have not seen them during training. TS conformer ensemble generators tackle this challenge by sampling conformers with constrained atom positions, distances, and/or angles for the reaction center, where the original positions are normally taken from a TS template provided by the user.^5^ This approach is also used by CREST, the state-of-the-art TS conformer generator (see Figure 1a).^15^ For TS optimizations, constrained metadynamics runs are performed with positions, distances, and/or angles between the reaction centers constrained.^5^ The metadynamics simulations are run using the established semiempirical tight binding methods of the extended tight binding (xTB) family,^56^ allowing to optimize conformers and calculate their energies with relatively little computational cost compared to higher-level computational methods, such as DFT. The GNF-FF force-field method can also be employed.^57^ Throughout the conformer search, conformers are consistently filtered based on their energies as well as the root-mean-square-deviation (RMSD) of the atomic coordinates, removing duplicate and irrelevant high-energy conformers. Through multiple rounds of metadynamics, the conformer space is explored while ensuring that only relevant and low-energy conformers are considered. Whereas CREST is considered state-of-the-art for open-source conformer generators, recently GOAT^16^ has been released as part of the popular ORCA quantum chemistry suite.^58,59^ GOAT is a conformer sampling algorithm that explores conformer space through multiple uphill–downhill cycles (Figure 1b). Starting from a user-provided conformation, the gradient is projected and the structure is perturbed in a direction of increasing energy, until a barrier is crossed. After barrier crossing, the structure is optimized toward a new minimum, to give potentially a new conformer, that is then kept or discarded, based on their similarity to already sampled conformers. Conformer searches with GOAT can be performed on multiple levels of theory such as DFT or semiempirical tight binding (xTB). For TS searches, constrain/fix to CREST, the atomic positions, bonds, and/or angles in the reaction center are constrained or fixed. Both CREST and GOAT rely on multiple quantum chemical or force-field calculations throughout their conformer searches. While these calculations in principle ensure that physically reasonable and meaningful conformers are sampled, they contribute to the significant computational cost and therefore also environmental footprint of a computational pipeline.^60^

**Figure 1:**
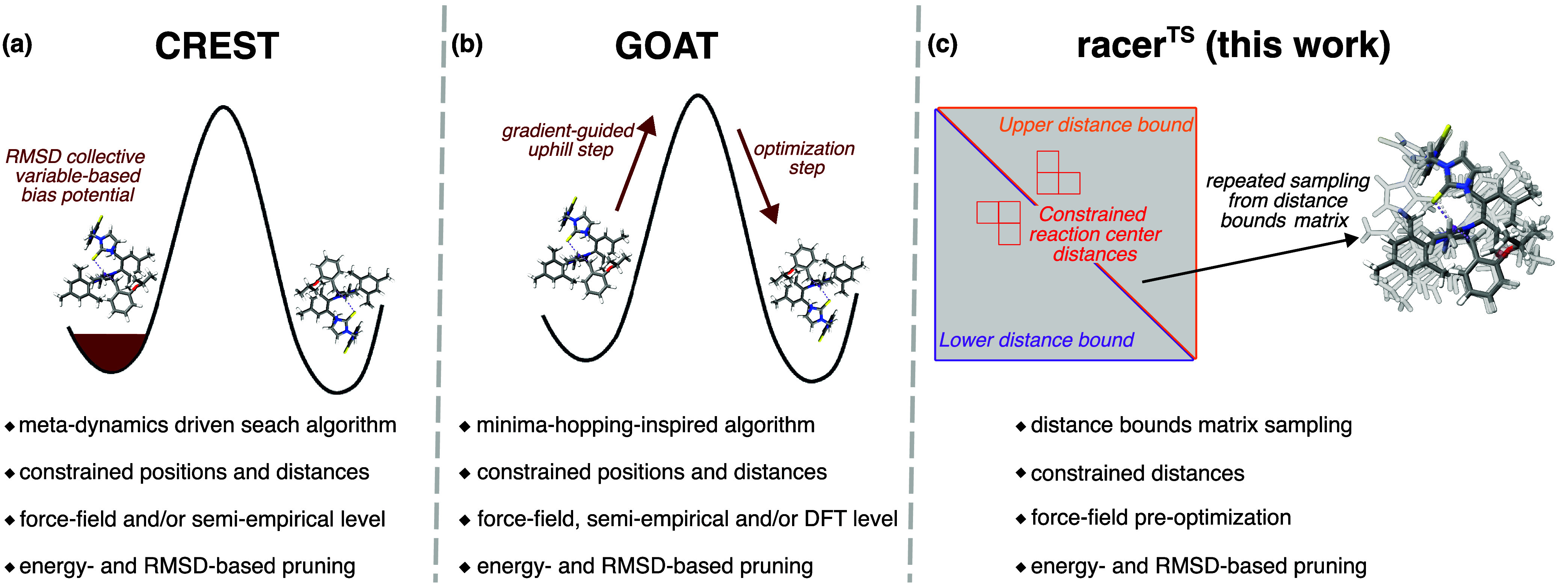
State-of-the-art open-source methods in TS conformer ensemble generation. (a) CREST performs metadynamics simulations to sample the conformer space. (b) GOAT uses a minima-hopping algorithm that projects the gradient of a structure to walk uphill on the energy landscape, followed by minimization to a new conformer. (c) We propose racer^TS^, generating TS conformers via sampling from a constrained distance bounds matrix.

When evaluating TS conformer ensemble generators, an ideal one can be considered to have five distinct properties: (i) be computationally inexpensive, (ii) be exhaustive (*i.e.*, sample the entire conformer space), (iii) produce valid structures (*i.e.*, provide structures that correspond to the desired TS), (iv) be accurate in the low-energy region (*i.e.*, find low-energy conformers and assign them as such), and (v) be easily integrable into computational chemistry workflows. As outlined above, we find that, in contrast to ground-state conformer searches, no fast conformer ensemble generation method is widely employed for transition states. In pioneering studies, the groups of West,^61,62^ and Reiher and Corminboeuf^63^ have investigated the usage of the distance geometry algorithm (the basis of RDKit’s ETKDG algorithm) to quickly sample TS conformers. The group of West used RDKit’s distance geometry algorithm to sample conformers of nonreactive parts of the TS while estimating distances in reaction centers via group contribution methods instead of via an input structure.^61^ Based on this methodology, the authors developed the AutoTST package^62^ and evaluated it based on three small-molecule reaction families involving only C, H, and O atoms. Given the small studied chemical space, the absence of comparison with other conformer generators, and differences in the employed methodology (see the Supporting Information), distance-geometry-based conformer generators for transition states remain understudied. The Reiher group integrated the capability to sample TS conformers in their Molassembler package,^64^ the utility of which was subsequently demonstrated in a study to predict the enantioselectivity of Rh­(III)-catalyzed asymmetric C–H activation reactions.^63^ While these pioneering studies show both the desire and utility of fast TS conformer generation methods, we find that currently no software exists that provides a fast, validated, and complete conformer generation pipeline for TSs in a user-friendly software. The lack of such a software significantly hinders high-throughput computational catalyst screening, as well as the creation of physically meaningful TS data sets that can be used to advance machine learning methods in catalyst discovery.

To address the lack of a software package that contains the complete pipeline for fast TS conformer generation, we propose “**RA**pid **C**onformer **E**nsembles with **RDK**it for **T**ransition **S**tates” (racer^TS^), a rapid TS conformer ensemble generation tool. Inspired by the works of West, Reiher, and Corminboeuf, racer^TS^ is based on a modification of RDKit’s standard ETKDG method, sampling transition states according to the distance geometry algorithm, followed by the necessary postprocessing steps. The sole dependence on the established cheminformatics package RDKit makes racer^TS^ easily integrable into existing cheminformatics workflows while rapidly providing exhaustive and reasonably accurate conformer ensembles. In the following, we describe the working principle of racer^TS^. To show its utility in computational chemistry workflows, we benchmark its performance in TS conformer sampling against CREST and GOAT based on the five desirable properties of a conformer ensemble generator.

## Methods

### Generating Transition-State Conformer Ensembles with racer^TS^

racer^TS^ is designed to work similarly to CREST and GOAT in that the user needs to provide the 3D structure of a TS guess (*i.e.*, an xyz coordinate file), the total charge, and a list of atom indices for the reaction center (see Figure 2, top). Optionally, a user can additionally provide the SMILES strings of either the reactants or the products. From this input, racer^TS^ generates an xyz coordinate file that contains multiple conformers of the input transition state. In this section, the working principle of the default implementation of racer^TS^ is described. An extensive user tutorial detailing further possible modifications to the default algorithm is provided in the Supporting Information.

**Figure 2:**
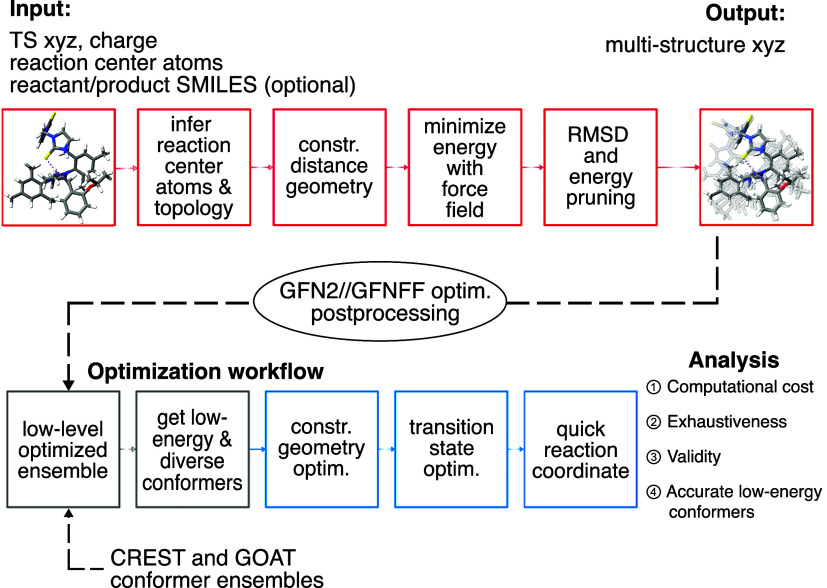
Top: Workflow of racer^TS^. The molecular topology is inferred based on an input xyz file of one transition-state conformer, a list of reaction center atoms, and optionally SMILES strings of reactant(s) or product(s). A constrained distance geometry conformer generation is performed, conformers undergo energy minimization with a force field and are further pruned based on RMSD and energetic criteria. As output, an xyz file with a conformer ensemble is obtained. Bottom: DFT optimization workflow. racer^TS^ conformer ensembles are postprocessed via constrained optimization at the GFN2-xTB//GFN-FF level. For all investigated conformer generators, low-energy and representative conformers are selected, and a constrained geometry optimization, TS optimization, and QRC with DFT is performed. The results are used to analyze the performance of conformer generators with respect to the computational cost, exhaustiveness, validity, and accuracy of the low-energy region.

In a first step, the user-provided 3D structure and total charge (and potential SMILES) are used to generate an RDKit Mol object using the RDKit rdDetermineBonds module.^65^ This Mol object contains information on bond types and formal charges, which is either inferred from the 3D structure directly or taken from the user-supplied SMILES of the product(s) or reactant(s), if either is provided. Assigning atom and bond types for reaction centers is often error-prone, as the reaction center does not display typical bonds and valencies. For these atoms and bonds, the program accepts any valid RDKit assignment, as correct labeling is not strictly required, since these atoms and bonds are constrained throughout conformer generation and refinement. If no assignment of bond orders is possible (*e.g.*, as often encountered for transition-metal complexes), racer^TS^ defaults to inferring solely the connectivity, followed by sanitization (see the Supporting Information).

In the next step, the obtained Mol object is used to generate the initial TS conformers. Similar to RDKit’s conformer generation method for ground-state conformers, racer^TS^ relies on the highly efficient ETKDG method for rapid TS conformer generation. To obtain reasonable conformers, ETKDG takes experimental torsional angles and geometry heuristics into account for the distance geometry method.^17,18^ For transition states, to conserve the critical reaction center, racer^TS^ constrains the corresponding atom positions. Additionally, during development, we found that constraining the distances between reaction center atoms and the neighbors of all reaction center atoms leads to more reliable TS conformers. While reacting atoms are the required input by the user, their neighbors are automatically inferred via connectivity assignment from RDKit. The unification of reaction centers and their immediate neighbors will be referred to below as the *frozen atoms*. Multiple conformers are subsequently generated by repeatedly sampling random distances from the constrained distance bounds matrix, followed by heuristic structural refinement as specified in the ETKDG method.^17,18^

After initial generation, the obtained conformers need further structural refinement.^39,41^ As a default, the energy of all generated conformers is minimized with the RDKit implementation^66^ of the MMFF94 force field,^67^ which is parametrized for organic molecules. One failure mode is unrecognized atom types, which might be particularly relevant for reaction centers (*e.g.*, radicals assigned to reaction centers) and for organometallic reactions. As a fallback, refinement via the RDKit implementation of UFF^68^ is performed, for which all unrecognized atom types are assigned default parameters.

In the last step, the conformer ensemble is pruned to remove duplicates, considering energetic and structural factors. Similar to the structure criterion used by CREST and GOAT, a conformer is removed if it has an RMSD < 0.125 Å to a lower-energy conformer. In contrast to CREST, racer^TS^ considers only heavy atoms for RMSD calculations by default, focusing on removing highly similar conformers. To consider only conformers within a relevant energetic window, CREST prunes all conformers whose energy is higher than 6 kcal/mol to the energy of the lowest-energy conformer. Due to inaccuracies in force-field-derived energies,^69^ and to avoid removing relevant conformers, a more conservative energy window of 20 kcal/mol, according to force-field energies, is applied by default within racer^TS^. All refined conformers that are not removed in this step are then concatenated in a single multistructure xyz file as output.

### Benchmarking

#### Optimization Workflow

To assess the quality of the racer^TS^ -generated conformer ensembles, we compare them with those from the state-of-the-art methods CREST and GOAT. For benchmarking, we evaluate the five criteria of a desirable conformer generator, as discussed above. To assess the usability of racer^TS^ in high-throughput computational chemistry workflows, we take transition states of *n*_rxn_ = 20 reactions from the literature to generate TS conformer ensembles^5,70–84^ (see Figures S1–S4), including challenging organometallic complexes. While 20 reactions exceed the number of systems considered in other papers presenting conformer generators,^16^ benchmarking on this small number of reactions can lead to variances in results. By picking diverse chemistries, we ensure that the strengths and weaknesses of racer^TS^ are accurately portrayed. As reacting atoms, every atom was chosen that is part of at least one dashed bond as drawn in Figures S1–S4. The reactions are discussed in more detail in the Supporting Information. In the main text, we compare five different methods: (i) racer^TS^ using a 3D structure as input (called **racer** ^**TS**^), (ii) racer^TS^ using a 3D structure and reactant SMILES as input (called **racer** ^**TS**^ **SMILES**), (iii) racer^TS^ using a 3D structure as input and a reduced level of postprocessing (called **racer** ^**TS**^ **(no xTB)**), (iv) CREST at the GFN2-xTB//GFN-FF level with constrained frozen atoms positions and distances (called **CREST**), and (v) GOAT at the GFN2-xTB level (the cheapest available level at the time of writing), with GFN-FF uphill activated and autofrag deactivated and default settings otherwise. The positions of and distances between the frozen atoms were fixed (called **GOAT**). To increase accuracy of energetic conformer ranking in the methods **racer** ^**TS**^ and **racer** ^**TS**^ **SMILES**, all conformers are postoptimized using the xtb program^56^ with the GFN-FF force field^57^ and exact fixing of the frozen atoms and the final energy used for sorting evaluated with single-point calculations using GFN2-xTB.^85^ After postoptimization, high-energy conformers were again pruned with an energy window of 6 kcal/mol w.r.t. the lowest-energy conformer, and potential duplicate conformers were pruned with a threshold of heavy-atom RMSD < 0.125 Å to a lower-energy conformer. No such postoptimization is performed for **racer** ^**TS**^ **(no xTB)**. Further computational details and other tested variants of racer^TS^ and GOAT are described in the Supporting Information, as well as in Tables S1 and S2.

To compare the generated conformer ensembles, we apply a conventional high-throughput computational chemistry workflow to each ensemble (see Figure 2, bottom). As the first step in this workflow, we extract the five lowest-energy conformers for each method. Additionally, we also use the marc program^5^ to extract up to 10 representative conformers for each ensemble. Throughout this paper, the extracted conformers will be termed low-energy (*lowe*) conformers and *marc* conformers, respectively. The conformer extraction methods are designed to mimic how a computational chemist uses conformer ensemble output, focusing on low-energy (lowe) and diverse (marc) conformers, respectively. Each extracted conformer is then calculated at the DFT level at the r^2^SCAN-3c level of theory^86^ using ORCA version 6.0.1.^58,59,87–94^ In this step, a constrained geometry optimization is first performed with fixed distances between the reaction atoms, followed by a transition-state optimization and then a quick reaction coordinate (QRC)^95,96^ calculation to ensure that the transition state connects the desired reactants and products.

#### Metrics

To assess the performance of racer^TS^, we analyze the described TS conformer generation methods following the aforementioned five properties of an ideal conformer generator.

##### Computational Cost

To analyze the computational cost of method *i*, we consider the wall-time of each method and normalize by the wall-time of **racer** ^**TS**^ **(no xTB)**

$$\mathrm{comp}.\;{\mathrm{cost}}_{i}=\frac{1}{n_{\mathrm{rxn}}}\overset{n_{\mathrm{rxn}}}{\underset{r=1}{\sum }}\frac{t_{r}^{i}}{t_{r}^{{\mathrm{racer}}^{\mathrm{TS}}(\mathrm{no}\;\mathrm{xTB})}}$$ 1

where *t*_*r*_^*i*^ is the wall-time to generate all the conformers for reaction *r* with method *i*, including postoptimization for **racer** ^**TS**^ and **racer** ^**TS**^ **SMILES**, as discussed above. For fair comparison and to simulate the use case in high-throughput simulations, each calculation was performed on one CPU core.

##### Exhaustiveness

To analyze whether a TS conformer generator samples the entire relevant conformer space, we consider two metrics: *space exploration* and *space distribution*. For the space exploration metric, we first reoptimize all generated conformers at the GFN2-xTB//GFN-FF level, constraining the distances between the reaction center atoms from the original input structure. This reoptimization is required to ensure a fair structural comparison, as different methods output structures at different levels of theory (*e.g.*, **GOAT** outputs GFN2-xTB level structures, **racer** ^**TS**^ and **CREST** output GFN-FF level structures). We combine these reoptimized structures from all the five methods discussed in the main text and also all structures from the additional racer^TS^ and GOAT variants described in the Supporting Information. We again prune this combined ensemble with an energy window of 6 kcal/mol w.r.t. the lowest-energy conformer and a threshold of RMSD < 0.125 Å. We regard this combined ensemble as the best possible approximation of a complete reference TS conformer ensemble. Inspired by work from Iribarren *et al.*,^39^ all conformers from the combined ensemble are clustered based on pairwise RMSD, using Butina Clustering^97,98^ and an RMSD threshold of RMSD_thr_ = 1.0 Å. For a given reaction *r*, we can calculate the precision of method *i*, PRE_*i*_^*r*^ (how many conformers of method *i* are in a cluster of the combined ensemble and not filtered out in the combination step) and the recall of method *i*, REC_*i*_^*r*^ (how many clusters of the combined ensemble are represented by the conformers of method *i*) as

2 $${\mathrm{PRE}}_{i}^{r}=\frac{n_{\mathrm{in}\;\mathrm{cluster},\;r}^{i}}{n_{\mathrm{total},\;r}^{i}}$$

3 $${\mathrm{REC}}_{i}^{r}=\frac{n_{\mathrm{clusters},\;r}^{i}}{n_{\mathrm{total}\;\mathrm{clusters},\;r}^{i}}$$

where *n*_in cluster, *r*_^*i*^ is the number of conformers from method *i* and reaction *r* that are in a cluster of the combined ensemble, and *n*_total, *r*_^*i*^ is the number of conformers identified by method *i* for reaction *r. n*_clusters, *r*_^*i*^ is the number of clusters of the combined ensemble to which at least one conformer from method *i* belongs, while *n*_total clusters, *r*_^*i*^ is the total number of clusters in the combined ensemble for reaction *r*. From this, we can calculate the space exploration of method *i* as an average over the F1 scores of all reactions

4 $$\mathrm{space}\;{\mathrm{exploration}}_{i}=\frac{1}{n_{\mathrm{rxn}}}\sum_{r=1}^{n_{\mathrm{rxn}}}\frac{2\cdot {\mathrm{REC}}_{i}^{r}\cdot {\mathrm{PRE}}_{i}^{r}}{{\mathrm{REC}}_{i}^{r}+{\mathrm{PRE}}_{i}^{r}}$$

For the space distribution metric, we again use the combined ensemble as the best approximation of a complete TS conformer ensemble. For each reoptimized conformer (see above), we calculate the RMSD to the 3D geometry that was given as an input to all conformer generators (each conformer generator got the same 3D geometry as input) to obtain an RMSD distribution. The same is done for every reoptimized conformer (see above) of the combined ensemble to obtain a second RMSD distribution. For the space distribution of method *i*, we calculate the Jensen–Shannon divergence of the RMSD distributions of method *i* and the combined ensemble,^99^ and average it over all benchmarked reactions

5 $$\mathrm{space}\;{\mathrm{distribution}}_{i}=\frac{1}{n_{\mathrm{rxn}}}\sum_{r=1}^{n_{\mathrm{rxn}}}\mathrm{JS}\left(s_{\mathrm{rmsd},\;r}^{i},s_{\mathrm{rmsd},\;r}^{\mathrm{all}}\right)$$

where *JS*(*a*, *b*) is the Jensen–Shannon (*JS*) divergence between distributions *a* and *b*, *s*_rmsd, *r*_^*i*^ is the RMSD distribution between the input conformer and the reoptimized conformers for method *i* for reaction *r* and *s*_rmsd, *r*_^all^ is the RMSD distribution between the input conformer and the combined ensemble from all methods for reaction *r*. A low *JS* divergence means that the distribution of the true ensemble is well-reproduced by a method.

##### Validity

To assess whether conformer ensemble generators produce valid TS structures, we consider whether they can generate structures that correspond to the desired transition states. To determine the success rate of method *i*, we calculate the fraction of the filtered (low-energy and marc) conformers that successfully generate the desired TSs in the DFT pipeline

6 $$\mathrm{success}\;{\mathrm{rate}}_{i}=\frac{1}{n_{\mathrm{rxn}}}\sum_{r=1}^{n_{\mathrm{rxn}}}\frac{n_{\mathrm{successful},\;r}^{i}}{n_{\mathrm{total},\;r}^{i}}$$

where *n*_successful, *r*_^*i*^ is the number of successfully DFT-optimized transition states for method *i* and reaction *r*, and *n*_total, *r*_^*i*^ is the total number of conformers for method *i* and reaction *r*. Conformers are considered valid if all calculations converge without errors and produce a transition state that connects the desired reactants and products, as evaluated through the QRC calculation. The latter was checked manually to ensure that no compounds are parsed erroneously, as automated parsing, in particular for transition-metal compounds with nontrivial bonding, can lead to incorrect bonding assignments. While automated high-throughput workflows involving more reactions will require automated parsing, one might be willing to accept a certain error rate as the price of automation^42,45,100,101^ or create specialized parsers for the systems of interest. Importantly, we want to highlight that the validation workflow is identical for all methods and does not affect the output of the conformer generators. Notably, we only perform one iteration of the DFT pipeline to simulate a truly high-throughput computation routine where error rates between 10 and 50% are considered state-of-the-art.^42,45,100,101^ The reported validity would therefore be an underestimation compared with a more elaborate TS optimization workflow (potentially with error correction).

##### Accurate Low-Energy Conformers

To investigate whether the conformer ensemble generators are accurate for the highly relevant low-energy conformers, we consider two metrics, *Top-N accuracy* and Δ*E* ^‡^. To assess the identification of low-energy conformers for method *i*, we consider the Top-N accuracy and evaluate how often the overall lowest-DFT-energy conformer across all methods is present in the top-1 or top-5 low-energy conformers of that method

$$T\mathrm{op}‐1_{i}=\frac{1}{n_{\mathrm{rxn}}}\overset{n_{\mathrm{rxn}}}{\underset{r=1}{\sum }}I\left[R_{r,\;i}\left(c_{r}^{n_{\mathrm{lowest},\;\mathrm{all}}}\right)=1\right]$$ 7

and

$$T\mathrm{op}‐5_{i}=\frac{1}{n_{\mathrm{rxn}}}\overset{n_{\mathrm{rxn}}}{\underset{r=1}{\sum }}I\left[R_{r,\;i}\left(c_{r}^{\mathrm{lowest},\;\mathrm{all}}\right)\leq 5\right]$$ 8

where *c*_*r*_^lowest, all^ is the overall lowest-DFT-energy conformer for reaction *r. R_r_*_,_ *_i_* (*c*_*r*_^lowest, all^) indicates at which position a conformer is found that is identical to the overall lowest-energy conformer. For energy ranking of the conformers, we consider the ranking by the original TS ensemble generator; *i.e.*, the conformer that is judged lowest in energy by a method *i* is considered for the top-1 accuracy, neglecting potential reordering by DFT. This choice is motivated by how the methods would be used in high-throughput workflows. Conformers are considered to be identical if they have an RSMD < 0.125 Å and an energy difference of Δ*E* < 0.05 kcal/mol.

To determine the utility of using the lowest-energy conformers to compute activation energies (*E* ^‡^), we calculate the error in *E* ^‡^ prediction for the lowest-energy conformer as well as the error for a Boltzmann-average of marc-selected conformers for each method. The first metric is highly similar to the one presented in the original publication of GOAT,^16^ where the energy of the lowest-energy conformer for each method is compared. However, the metric presented here is geared toward activation energy calculations, which is the quantity of interest for practitioners in TS calculations. To compute the error for low-energy conformers, we determine the overall lowest-energy conformer at the DFT level and the lowest-energy conformer for each method *i*

9 $$\Delta E_{\mathrm{lowe}}^{‡}=E\left(c_{r}^{\mathrm{lowest},\;i}\right)-E\left(c_{r}^{\mathrm{lowest},\;\mathrm{all}}\right)$$

where *E*(*c*) is the DFT energy of conformer *c*, *c*_*r*_^lowest, all^ is the conformer for reaction *r* with the lowest energy among all DFT conformers, and *c*_*r*_^lowest, *i*^ is the conformer for reaction *r* with the lowest energy among the DFT conformers of method *i*. Note that to get *c*_*r*_^lowest, *i*^, we only consider the conformers that were extracted due to their low energy and that went through the TS optimization pipeline successfully (see above). It might therefore be that some lower-energy conformers failed to optimize. Again this behavior reflects how the workflow would typically be used in high-throughput simulations. To compute the utility of racer^TS^ for more elaborate *E* ^‡^ computations, we compute the difference between the Boltzmann-averaged activation energies of marc conformers of method *i* (maximum of 10 conformers) and the Boltzmann-averaged activation energies of all marc conformers (not limited in number, but pruned to remove duplicates)

10 $$\Delta E_{\mathrm{marc}}^{‡}=E_{B}\left(ce_{r}^{\mathrm{marc},\;i}\right)-E_{B}\left(ce_{r}^{\mathrm{marc},\;\mathrm{all}}\right)$$

where *E*_B_(*ce*) is the Boltzmann-averaged energy of a conformer ensemble, *ce*_*r*_^marc, *i*^ is the ensemble of marc conformers for method *i* and reaction *r*, and *ce*_*r*_^marc, all^ is the pruned conformer ensemble of all marc conformers of reaction *r*.

##### Usability

Usability is a subjective criterion that cannot be rigorously assessed with a metric. CREST and GOAT are established quantum chemistry softwares that are easily usable via the command line. racer^TS^ can be used both via the command line and in Python as a lightweight pip-installable package that can be run locally. By having RDKit as its sole dependency, we ensure that it is easily integrated into existing cheminformatics workflows.

Focusing on all the above-described metrics allows us to thoroughly assess the performance of racer^TS^ compared to state-of-the-art TS conformer ensemble generators, showcasing how racer^TS^ can be successfully used as a TS conformer ensemble generator.

## Results and Discussion

To study the strengths and weaknesses of racer^TS^ as a conformer generator, we benchmark it against CREST and GOAT. The following sections describe the benchmarking results on the computational cost, exhaustiveness, validity, and accuracy in the low-energy region.

### Computational Cost

A key driver for the development of racer^TS^ is the requirement for an accurate but significantly faster TS conformer ensemble generator. Figure 3 shows the runtime to generate each conformer ensemble, normalized by the time of **racer** ^**TS**^ **(no xTB)** for each reaction (see eq 1). Overall, we find that using racer^TS^ can provide a 4× speed-up over **CREST**, and 500× over **GOAT**. Such speed-ups can be expected, given the established computational efficiency of the distance geometry algorithm. The large speed-up compared to **GOAT** can also be rationalized by the fact that, at the time of writing, **GOAT** could only be run on the GFN2-xTB level, which is considerably slower than GFN2-xTB//GFN-FF but also produces higher quality structures. In contrast, since **CREST**, **racer** ^**TS**^ and **racer** ^**TS**^ **SMILES** all produce structures on the GFN2-xTB//GFN-FF level, the increase in efficiency for **racer** ^**TS**^ and **racer** ^**TS**^ **SMILES** therefore demonstrates a faster sampling of the distance geometry method over metadynamics. Notably, the timing difference between **racer** ^**TS**^/**racer** ^**TS**^ **SMILES** and **racer** ^**TS**^ **(no xTB)** is solely due to the GFN2-xTB//GFN-FF postoptimization within **racer** ^**TS**^/**racer** ^**TS**^ **SMILES**, accounting for approximately three-quarters of the total runtime (see Table S3). While the lower level of theory within **racer** ^**TS**^ **(no xTB)** leads to significant reduction in the required wall-time, structures and energy ranking of conformers is of poorer quality. In the following, analysis on accuracy in the low-energy region (see below) indicates that postoptimization is required to provide more accurate conformer ensembles. In spite of this, if the ranking of conformers is not decisive for the application (*e.g.*, when the entire ensemble is optimized at a higher level in any case), the speed-up provided by racer^TS^ can be even more pronounced. Thus, our results indicate that racer^TS^ achieves the desired significant speed-up, reducing computational cost by orders of magnitude compared to state-of-the-art methods.

**Figure 3:**
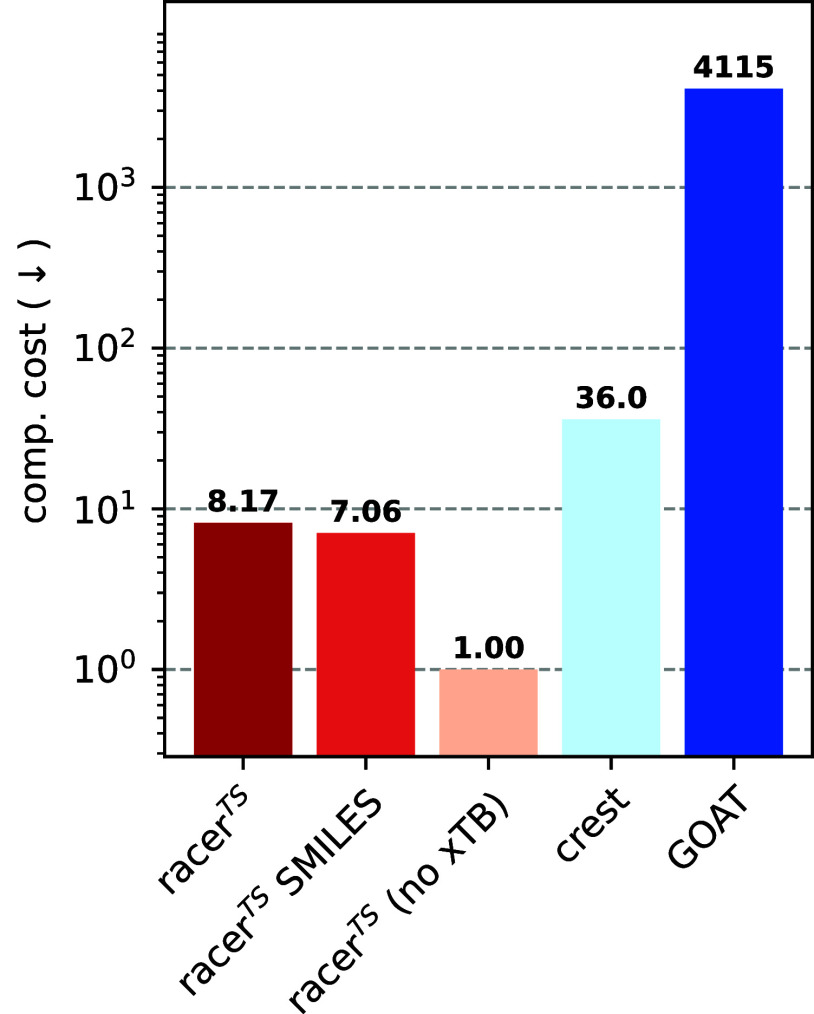
Average run time of conformer ensemble generators, normalized to the run time of **racer** ^**TS**^ **(no xTB)** (eq 1). Note the logarithmic scale.

### Exhaustiveness

To study the exhaustiveness of each method in exploring conformer space, we begin by looking at the number of conformers for each reaction (Table 1). As a general trend, we observe that **CREST** produces the highest number of conformers, doing so for 16 of the 20 benchmarked reactions (bold in Table 1). This trend is consistent with earlier investigations.^39^ We hypothesize that this observation can be partly rationalized by a combination of less stringent fixing of frozen atoms (**CREST** does not allow exact fixing, only constraining in contrast to **racer** ^**TS**^ and **GOAT**; see Figure S10) and less strict RMSD pruning (**CREST** also considers hydrogen atoms in the RMSD pruning while **racer** ^**TS**^ does not). On the contrary, we find that no method consistently produces the fewest conformers, with **racer** ^**TS**^ doing so for 11 reactions, **racer** ^**TS**^ **SMILES** for 4, and **GOAT** for 6 reactions (underlined in Table 1). Comparing the number of conformers produced by **racer** ^**TS**^ and **racer** ^**TS**^ **(no xTB)**, one clearly sees that GFN2-xTB//GFN-FF postoptimization, followed by a tighter energy filtering, removes many high-energy conformers. On average only 41% of the conformers from **racer** ^**TS**^ **(no xTB)** remain in the postoptimized **racer** ^**TS**^ ensemble. Comparing the number of conformers from **racer** ^**TS**^ and **racer** ^**TS**^ **SMILES** shows that they are consistently within the same order of magnitude, suggesting that both topology inference directly from the 3D structure and taking the topology from the reactant SMILES are suitable methods. In fact, most of the notable deviations in conformer numbers between **racer** ^**TS**^ and **racer** ^**TS**^ **SMILES** can be explained by only being able to infer the connectivity and not the complete bonding within **racer** ^**TS**^, as seen, *e.g.*, for the Ru olefin metathesis reaction. Another reason for large differences in conformer numbers appears when a method finds a conformer with a particularly low energy, leading to more pruned (high-energy) conformers.

**Table 1:** Number of Conformers Generated per Method for Each of the Investigated Reactions^a^

reaction	**racer** ^**TS**^	**racer** ^**TS**^ **SMILES**	**racer** ^**TS**^ **(no xTB)**	**CREST**	**GOAT**
amide methylation	10	11	16	**119**	34
cyclization	17	19	33	**34**	1
epoxidation	4	10	9	**426**	10
epoxide rearrangement	28	39	37	**53**	48
hydride transfer	21	26	40	**228**	47
hydroborylation	7	5	15	**104**	24
NHC	93	70	115	**1426**	215
nucleophilic addition	35	57	143	**264**	192
peptide	2	3	**97**	86	21
pericyclic	24	23	**48**	6	3
propargylation	9	9	31	**232**	3
proton transfer	11	5	39	**740**	1
S_N_2	2	4	2	**181**	9
S_N_2 sugar	2	19	106	14	**182**
S_N_Ar	14	4	23	**214**	1
tropane alkylation	5	7	16	**806**	7
Pd carbofluorination	11	3	**55**	17	7
Pd oxidative addition	2	2	8	**111**	71
Ru olefin metathesis	14	57	173	**408**	181
Ti elimination	7	6	21	**266**	4

a The highest number of conformers is marked in bold, while the lowest number is underlined.

While the number of conformers can be indicative of the explored conformer space, this metric does not directly account for diversity, as many highly similar conformers could be found even after RMSD pruning. To investigate how the conformer space is covered, we calculated the space exploration metric (eq 4) for each investigated method. As shown in Figure 4a, **GOAT** offers the best capabilities in conformer space exploration with an average F1 score of 0.93, while all three racer^TS^ variants and **CREST** perform similarly, with an average F1 score between 0.74 and 0.80 for all four methods. The F1 scores offer a trade-off between precision (eq 2) and recall (eq 3) of a method and are only high when both components are high. A closer investigation into these metrics for each method reveals that for all methods with exception of **racer** ^**TS**^ **(no xTB)**, the precision (*i.e.*, how many conformers of a method are in clusters of the true ensemble, eq 2) is close to 1, demonstrating that essentially all conformers produced with these methods belong to the complete ensemble. The only exception is **racer** ^**TS**^ **(no xTB)**, with a significantly lower precision of 0.84. This result suggests that some conformers generated with **racer** ^**TS**^ **(no xTB)** do not belong to the complete ensemble, which was obtained by considering optimization and a second, stricter energy pruning. Thus, this result is expected, as **racer** ^**TS**^ **(no xTB)** does not undergo a second pruning with a stricter energy threshold. The high-energy conformers that are not removed via this pruning often do not belong to one of the clusters of the combined ensemble, thus decreasing the observed precision.

**Figure 4:**
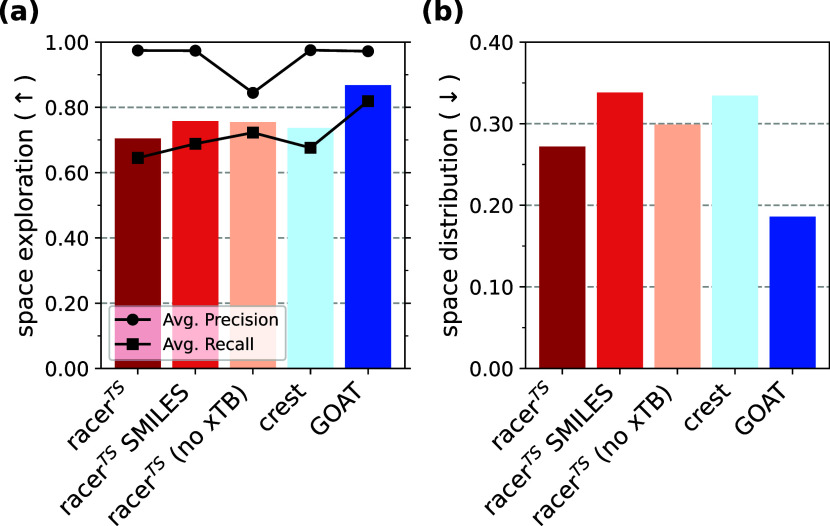
Comparison of exhaustiveness metrics for the five benchmarked conformer generators. (a) Space exploration as determined by eq 4 for each method. (b) Space distribution as determined by eq 5 for each method.

With precisions near unity for most methods, the recall (*i.e.*, how many clusters of the complete ensemble are identified by each method; eq 3) is the limiting factor for space exploration. Indeed, for all racer^TS^ variants as well as **CREST**, this metric lies between 0.65 and 0.72, significantly below 1. Meanwhile, the superior space exploration performance of **GOAT** can be explained by its superior recall of 0.82, far outperforming the other methods. The inferior recall of **CREST** is initially surprising, as **CREST** produces the highest number of conformers for a vast majority of reactions. However, this observation suggests that the metadynamics-based **CREST** produces many similar conformers that are not pruned, likely due to perturbed reaction centers (Figure S10). This observation again demonstrates that the number of conformers does not necessarily correlate with how much of the conformer space has been explored. In contrast, the inferior recall of racer^TS^ variants can be explained by their lower number of produced conformers, resulting in some clusters of the combined ensemble not being populated. In particular, **racer** ^**TS**^ has the lowest number of conformers for 11/20 reactions and also the lowest recall, with 0.65. Within racer^TS^, the number of initial conformers generated (before any pruning) can be easily tuned by passing the argument conf_factor (default value 30). More initially generated conformers lead to more discovered clusters and thus also to an increase in the F1 score, as shown in Figure S11. During development of racer^TS^, we have found 30 to be a good default value for conf_factor as it matches the performance of the state-of-the-art method **CREST**. However, in applications where the generation of all relevant conformers is critical, racer^TS^ can easily be modified to achieve a higher space exploration (Figure S11).

To further investigate the conformer space that is produced by each method, we investigate the distribution of RMSDs between the conformers of the ensemble and the input 3D geometry. This distribution is compared with the RMSD distribution computed for the combined ensemble (eq 5). Figures demonstrating the RMSD distributions for each conformer generator and for each reaction are depicted in Figures S12–S31. In agreement with the results for the space exploration metric, **GOAT** covers the space most completely as the observed RMSD distribution is the most similar to the distribution of the combined ensemble. This is evidenced by the average space distribution score of 0.19 (lower is better; see Figure 4b). Again confirming results from the space exploration metric, racer^TS^ variants show a similar JS divergence to **CREST**. This result again corroborates that the number of conformers does not necessarily correlate with the explored conformer space; since **CREST** produces many highly similar conformers (*vide supra*), the RMSD distribution is dissimilar from the distribution of the combined ensemble, where most of these conformers have been pruned.

Overall, our results indicate that **GOAT** is the most proficient method for exhaustively sampling the conformer space. While racer^TS^ performs on par with **CREST**, we also demonstrate how the settings of racer^TS^ can be easily modified to more exhaustively sample the conformer space, although at an increased computational cost.

### Validity

Next to exhaustive exploration of conformer space, conformer ensemble generators should also provide valid TS structures (eq 6). To assess this, we measure how many of the selected conformers provide a valid transition state at the DFT level after going through the optimization pipeline described above. Comparing all five methods, we find that all variants of racer^TS^ produce a higher fraction of valid TSs than both **CREST** and **GOAT** (Figure 5). All methods produce valid TSs with a high success rate between 82% (**GOAT**) and 90% (**racer** ^**TS**^ **SMILES**). These failure rates are typical for a high-throughput computation pipeline, where error rates between 10 and 50% are commonly encountered.^42,45,100,101^ The observation that racer^TS^ produces more valid TSs also translates when comparing the number of reactions for which at least one valid TS is identified. All racer^TS^ variants produce at least one valid TS for all 20 tested reactions while **CREST** and **GOAT** do so for only 19 and 17 reactions, respectively. **CREST** fails to produce a valid TS conformer for the S_N_2 sugar reaction, while **GOAT** fails for the hydroborylation, NHC, and Ru olefin metathesis reactions, respectively. In the case of **GOAT**, the hydroborylation and NHC reactions fail due to geometry convergence issues in the computational pipeline, while the identified TS for the Ru olefin metathesis leads to the reactant and product of a different reaction after performing QRC.^95,96^ Using **CREST** as a conformer generator, the only reaction with no valid TS is S_N_2 sugar, where the identified TSs lead to the same reactant and product after QRC. In contrast to the failures of **CREST** and **GOAT**, racer^TS^ is able to produce valid TSs for every tested reaction, surpassing the state-of-the-art methods. The capability of racer^TS^ to generate valid TSs in all cases demonstrates its capability for a broad variety of applications, including highly complex, flexible, and organometallic systems.

**Figure 5:**
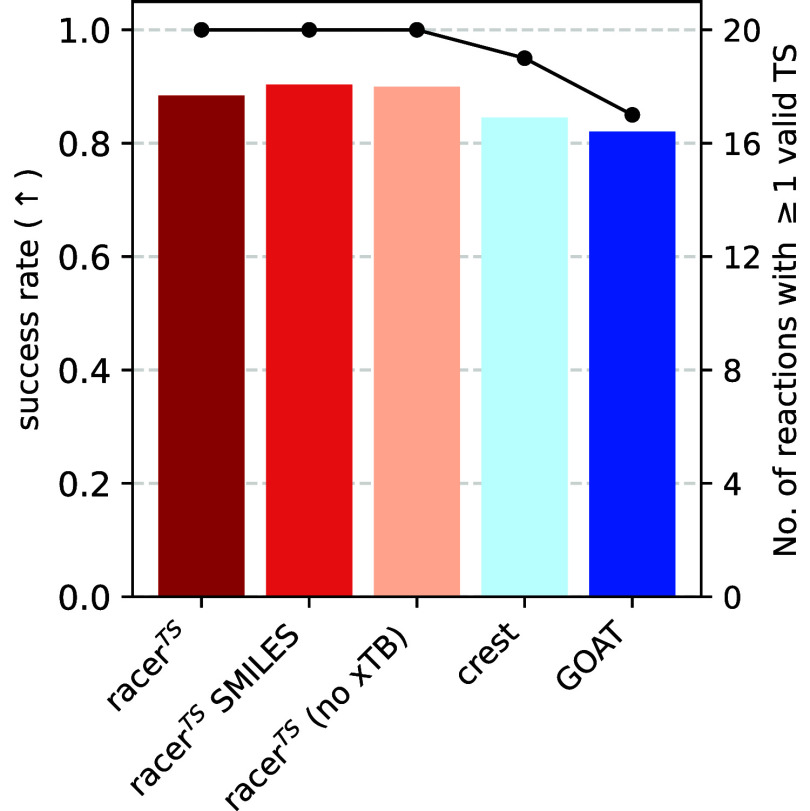
Success rate of conformers producing correct transition states in the benchmarking DFT pipeline (eq 6, left). For each method, conformers are selected, and transition states are optimized with DFT. The fraction of DFT transition states connecting the desired reactants and products is assessed and averaged over all reactions. The black dots show the number of benchmarked reactions with at least one successfully converged conformer.

### Accurate Low-Energy Conformers

While consideration of entire conformer ensembles is becoming more common within computational chemistry due to computational efficiency, activation energies are often calculated by considering only the lowest-energy conformer of the transition state. As low-energy conformers contribute the most toward the ensemble energy due to having the highest Boltzmann weight, correctly identifying low-energy conformers is highly relevant for every conformer ensemble generator. To study whether the benchmarked conformer ensemble generators are accurate in the low-energy region, we consider two metrics.

We first determine the overall lowest-energy conformer at the DFT level found by any method. Then, we assess whether the extracted low-energy conformers for each method provide a conformer that is identical to the lowest-energy conformer. We consider top-1 (eq 7) and top-5 (eq 8) accuracy, *i.e.*, does the lowest-energy conformer and the five lowest-energy conformers (as ranked by the ensemble generators) contain a conformer identical to the overall lowest-energy conformer. Overall, the fraction of identified lowest-energy conformers is surprisingly low (Figure 6), with **racer** ^**TS**^ showing the highest top-1 accuracy at 25% (5/20 reactions). Other methods identify the lowest-energy conformer correctly for three (**racer** ^**TS**^ **(no xTB)** and **GOAT**) or four (**racer** ^**TS**^ **SMILES** and **CREST**) reactions. For **racer** ^**TS**^, the correctly top-1 identified low-energy conformers are for the cyclization, epoxide rearrangement, pericyclic, propargylation, and S_N_Ar reactions. Considering top-5 accuracies moderately increases the fraction of identified lowest-energy conformers for most methods, with values up to 35% (7/20) reactions attained. A detailed description of which low-energy conformers are found by which method is listed in Table S5. We hypothesize that this relatively low retrieval rate is due to inaccurate ranking of conformers at the semiempirical level or force-field level. For 5 reactions (25%), the overall lowest-energy conformer was only found among the *marc* and not the *lowe* conformers, *i.e.*, ranked as low-energy by DFT, but not by the semiempirical methods. For the other 75% of reactions, the identified lowest-energy conformer is spread throughout the conformer generators, with racer^TS^ performing comparatively to the state-of-the-art. Considering the high conformer space exhaustiveness of all methods, it can be assumed that more conformers that would be low in energy at the DFT level are actually generated, but not ranked as such by the semiempirical methods. This oversight of crucial conformers has also been identified by other works.^102^ This hypothesis, which is supported by the lower accuracy of **racer** ^**TS**^ **(no xTB)**, can only be confirmed by optimizing every single conformer at the DFT level, which is out of scope of this study. The biggest improvement in accuracy can thus be achieved by optimizing and evaluating every conformer at the DFT level, or other, more recent semiempirical methods^103^ or machine learning potentials.^104^ Our results confirm racer^TS^ as an efficient conformer screener with an accuracy that matches or exceeds the current state-of-the-art.

**Figure 6:**
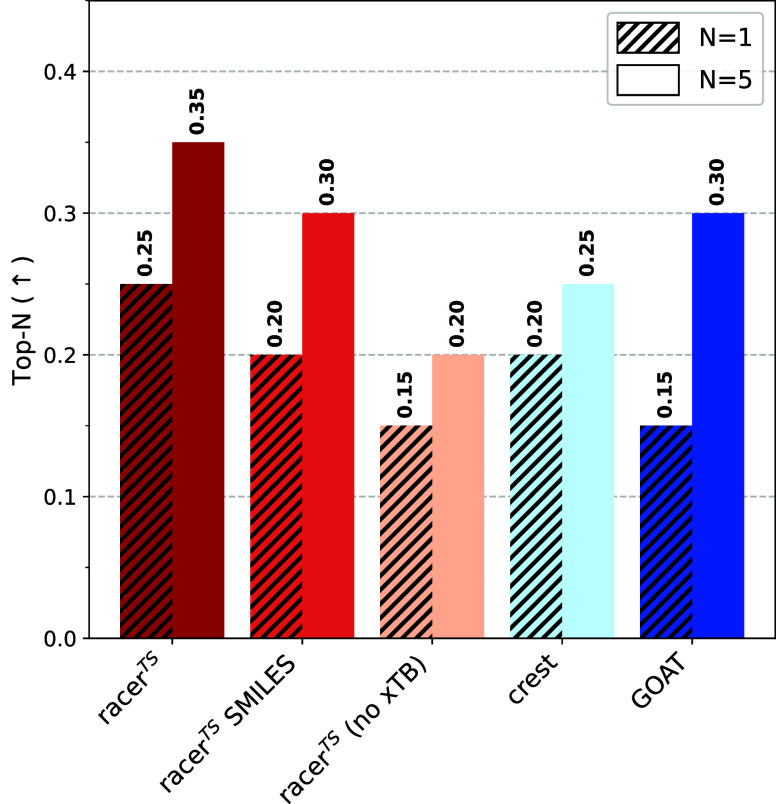
Top-1 (hatched) (eq 7) and top-5 (eq 8) accuracy in identifying the overall lowest-energy conformer.

To further explore the accuracy of the conformer generators for low-energy conformers, we investigate the deviations in calculating activation energies. First, we investigate the activation energy deviation when the lowest-energy conformer identified by each method is picked compared to the overall lowest-energy conformer (Δ*E*_lowe_^‡^; eq 9). Second, we also investigate the deviations in activation energies of the selected marc conformers for each method and an overall marc ensemble (Δ*E*_marc_^‡^; eq 10). Comparing Δ*E*_lowe_^‡^ (Figure 7, top) across conformer ensemble generators, we see that all racer^TS^ variants enable accurate activation energy calculations. For each of these methods, the median Δ*E*_lowe_^‡^ is significantly lower than the chemical accuracy (1 kcal/mol). For all methods, with the exception of **racer** ^**TS**^ **(no xTB)**, Δ*E*_lowe_^‡^ < 0.18 kcal/mol, showcasing the suitability of racer^TS^ for activation energy calculations. The comparatively high median deviation for **racer** ^**TS**^ **(no xTB)** is explained by energy ranking via MMFF or UFF, while the other conformer generators are ranked at the GFN2-xTB//GFN-FF level. Inaccurate rankings lead to conformers with higher energy being estimated as low in energy, resulting in higher Δ*E*_lowe_^‡^. Nonetheless, a median Δ*E*_lowe_^‡^ well below chemical accuracy for all methods demonstrates that each method is suitable to find low-energy conformers in the “average” use case, although individual reactions of interest can of course be higher than the mean. Shown by the broader distributions in Figure 7, we find that racer^TS^ variants are more likely to produce high-energy outliers in special cases. As significant outliers (Δ*E*_lowe_^‡^ > 5 kcal/mol), we observe amide methylation, epoxidation, peptide, S_N_2 sugar, and Pd oxidative addition reactions for multiple racer^TS^ variants. A more thorough investigation in these reactions shows two major factors leading to high-energy TS conformers: unfavorable rotamers with respect to amide bonds leading to steric clashes for TSs (amide methylation and peptide) and a propensity to generate boat-like structures for some six-membered rings (epoxidation, S_N_2 sugar, and Pd oxidative addition). A larger number of sampled conformers could plausibly mitigate some of these problems at an increase of computational cost. **CREST** also showed an outlier for the epoxidation reaction, where the conformer space of the fused rings is too rigidly explored, and thus, the lowest conformer is not identified.

**Figure 7:**
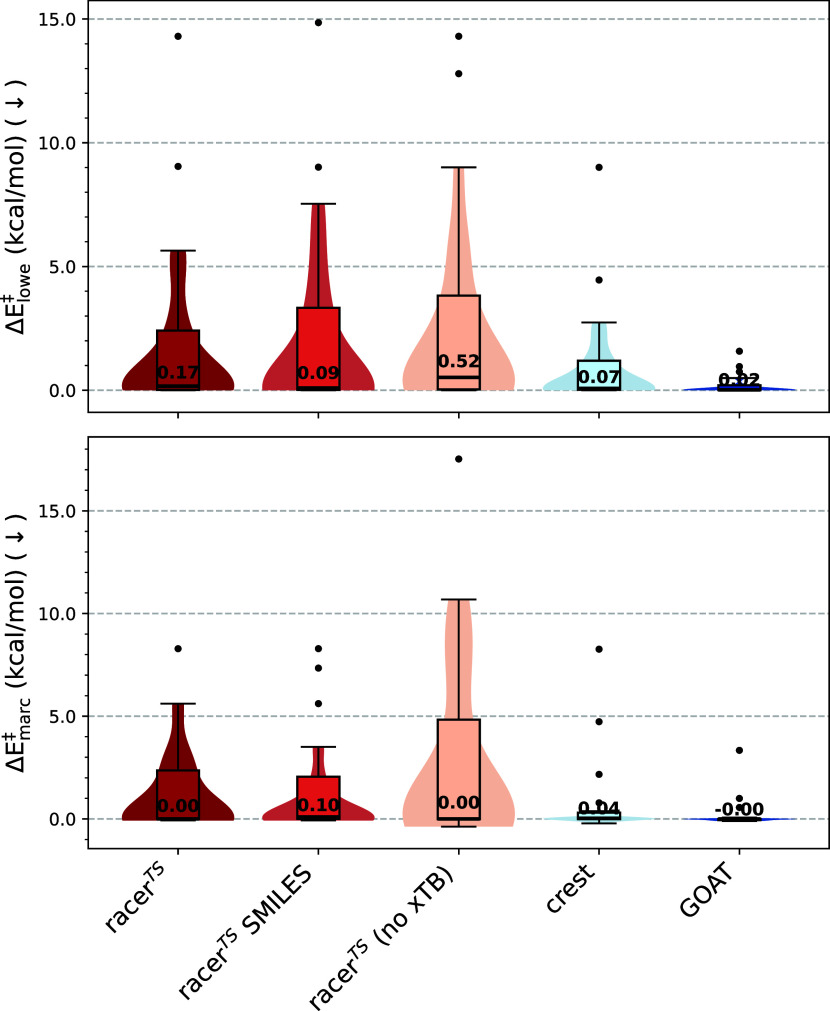
(Top) Tukey boxplot of Δ*E*_lowe_^‡^ (eq 9) for each method over all benchmarked reactions. (Bottom) Tukey boxplot of Δ*E*_marc_^‡^ (eq 10) for each method over all benchmarked reactions. The numerical value of the median is provided for all methods.

While calculating activation energies based on the lowest-energy conformer is still widely performed, more sophisticated methods, such as marc,^5^ consider multiple conformers that represent the Boltzmann distribution to achieve more accurate activation energies. To simulate this use case, we calculate activation energies based on marc-selected conformers (eq 10) for various conformer generators. Our observations show that the median deviation is Δ*E*_marc_^‡^ ≤ 0.1 kcal/mol for all conformer generators, again demonstrating the utility of racer^TS^ in everyday use cases. Similar to investigations into Δ*E*_lowe_^‡^, we observe that racer^TS^ variants are also more prone to outliers when considering Δ*E*_marc_^‡^. Frequently occurring outliers with a high deviation (Δ*E*_marc_^‡^ > 5 kcal/mol) among racer^TS^ variants are the same as Δ*E*_lowe_^‡^, with reasons as explained above. Outliers for **CREST** are also consistent with those discussed above.

Overall, we observe that the highest outliers occur for **racer** ^**TS**^ **(no xTB)**, most likely due to the poor energy-ranking performance of MMFF/UFF compared to GFN2-xTB//GFN-FF. Thus, racer^TS^ has to be carefully applied in cases that are prone to outliers, as discussed above. Nonetheless, we show that racer^TS^ generates conformer ensembles that enable an accurate activation energy estimate for a majority of use cases. This observation showcases the utility of racer^TS^ to serve as a conformer ensemble generator in a computational chemistry workflow for both computing activation energies based on the lowest-energy conformer and more sophisticated selection strategies. Combined with our previous analysis, the results indicate that racer^TS^ provides reasonably accurate transition-state conformer ensembles, broad conformer space coverage, and high validity rate at a fraction of the computational cost of state-of-the-art methods.

## Conclusion and Outlook

In this paper, we present racer^TS^, a method for the rapid generation of transition-state conformer ensembles. racer^TS^ uses a modified variant of distance-geometry-based conformer generation, enabling the constrained distance sampling required to preserve reaction center atoms in transition states. Its sole dependency is the popular cheminformatics library RDKit, making it easily integrable into existing cheminformatics and computational chemistry workflows. The use of racer^TS^ is inspired by state-of-the-art transition-state conformer ensemble generators CREST and GOAT, which only require a 3D geometry of a transition state as an input, along with information about which atoms belong to the reaction center. By generating TS conformer ensembles for 20 reactions involving four organometallic reactions, we have compared racer^TS^ to CREST and GOAT for use in high-throughput computational chemistry workflows. While the variance of results can be considerable with only 20 considered reactions, we are confident that the diverse chemistry sampled, together with detailed discussion on outliers, gives a good understanding of the applicability and limitations of racer^TS^ and other conformer generators. Overall, we find that racer^TS^ performs on par or better than state-of-the-art methods in the exhaustiveness, validity, and accuracy of the low-energy conformers of the generated ensembles, although with outliers for a few reactions. Most importantly, racer^TS^ achieves these results while providing significant reductions in wall-time compared to CREST and GOAT, respectively. Such efficiency enhancements also enable a more sustainable use of computational chemistry methods.^60^ While we only considered computational cost on CPUs, another avenue to increase the computational throughput of conformer generators is via GPU acceleration, which was reported for CREST while our study was carried out.^105^ With the recently released package for GPU-accelerated RDKit functions,^106^ GPU acceleration might also prove useful for further lowering the computational cost of racer^TS^. Altogether, our results indicate that racer^TS^ is a highly efficient TS conformer ensemble generator. Thus, our method paves the way for efficiently creating large TS data sets, which can be used to advance ML methods for the discovery of newer, more sustainable catalysts.

## Supplementary Material



## Data Availability

All data and scripts used in this study are available on Zenodo: https://doi.org/10.5281/zenodo.17610186. The developed software is available under the following GitHub link: https://github.com/digital-chemistry-laboratory/racerts. This repository contains a link on the data to recreate this paper. This repository also contains an example script for the use of racer^TS^.

## Notes

Extended user manual for racer^TS^; details on benchmarking reactions; computational details for used conformer generators; details on additionally benchmarked variants of racer^TS^; analysis of results for additionally benchmarked variants of racer^TS^; analysis of additional investigation into reaction center conservation, influence of the conf_factor parameter, and input conformer sensitivity; and detailed results for every benchmarked reaction (PDF)
