# Supplementary material for: Rapid Generation of Transition-State Conformer Ensembles via Constrained Distance Geometry
Source: J Chem Inf Model. 2026 Feb 12;66(5):2777–90. doi: 10.1021/acs.jcim.5c02794 (PMC12977065; doi:10.1021/acs.jcim.5c02794)
Supplement: Supplementary file 1 [file ci5c02794_si_001.pdf]

Supporting Information

Rapid generation of transition-state conformer ensembles via constrained distance geometry

Stefan P. Schmid,[†,‡] Henrik Seng,[†] Thibault Kläy,[†] and Kjell Jorner[*,†,‡]

[†] Institute of Chemical and Bioengineering, Department of Chemistry and Applied Biosciences, ETH Zurich, Zurich CH-8093, Switzerland

[‡] NCCR Catalysis, Switzerland

E-Mail: kjell.jorner@chem.ethz.ch

The Supporting Information contains the following content:

- Extended user manual for racer^{TS}
- Details on benchmarking reactions
- Computational details for used conformer generators
- Details on additionally benchmarked variants of racer^{TS}
- Analysis of results for additionally benchmarked variants of racer^{TS}
- Analysis of additional investigation into reaction center conservation, influence of the `conf_factor` parameter, and input conformer sensitivity
- Detailed results for every benchmarked reaction

Supporting Information

Extended user manual of racer^{TS}

As discussed in the main text, racer^{TS} consists of multiple independent modules: a `molgetter` that assigns molecular topology and reacting center atoms from 3D coordinates, an `embedder` that performs constrained distance geometry sampling, an `optimizer` that optimizes the initial conformers with a force-field and a `pruner` that prunes irrelevant conformers. This section will discuss the details of each step and potential settings that are helpful for a user.

`molgetter`

The `molgetter` obtains an RDKit Mol object from the 3D coordinates of the initial transition state template provided by the the user. racer^{TS} provides three different options to generate a Mol object: (1) `MolGetterSMILES`, (2) `MolGetterBonds`, and (3) `MolGetterConnectivity`. The default option is `MolGetterBonds` if no SMILES string is provided and `MolGetterSMILES` otherwise. If `MolGetterSMILES` cannot successfully assign a molecular topology, `MolGetterBonds` is invoked, and lastly `MolGetterConnectivity` as a fallback option in case of further errors.

`MolGetterConnectivity` only determines the connectivity of the atoms in the provided structures, neglecting bond orders and formal charges. It uses the `DetermineConnectivity` function from the `rdkit.Chem.rdDetermineBonds` module to assign single bonds between the atoms based on only their interatomic distances and atom types. A partial sanitization with the sanitize flags: `SANITIZE_SETHYBRIDIZATION`, `SANITIZE_SETAROMATICITY`, `SANITIZE_SETCONJUGATION`, and `SANITIZE_SYMMRINGS`, as well as stereochemistry assignment (via `rdkit.Chem.AssignStereochemistryFrom3D`) is then used for further handling of the Mol object.

`MolGetterBonds` uses the `DetermineBonds` function from the `rdkit.Chem.rdDetermineBonds` module, combining the two functions `DetermineConnectivity` and `DetermineB

ondOrders. DeterminebondOrders attempts to assign the bond orders and atomic formal charges based on the connectivity and the user-provided molecular charge. Sanitization (via rdkit.Chem.SanitizeMol) and stereochemistry assignment (via rdkit.Chem.AssignStereochemistryFrom3D) are then performed on this Mol object for further handling. In our benchmark study, this function failed repeatedly for some reactions. The reason is that the module detects partial TS bonds as true bonds, leading to incorrect atom valences, which means that it is not always possible to assign the correct bond orders based on the connectivity.

To circumvent this failure mode but still obtain bond orders, racer^{TS} also includes MolGetterSMILES, that is based on the (optionally user-provided) SMILES of the reactant(s) or product(s). The first step of MolGetterSMILES is matching the atom numbering of the SMILES string to the transition state structure, for which the connectivity has initially been determined using the function DetermineConnectivity. The atom numbering can either be provided by the user via the atom maps in the SMILES string, or is assigned so that the atom numbers match the atom indices of the SMILES string through an iterative process (see below). First, the atom map numbers of the TS Mol object are set to be the negative values of the corresponding atom indices of the Mol object, so that unmatched atoms in the transition state molecule can be tracked. After this step, an iterative process of finding the maximum common substructure (MCS) between the unmatched atoms of the Mol object derived from the SMILES string and the Mol object derived from the TS structure is performed, followed by setting the atom map numbers in the TS Mol object. In the process, truncated Mol objects are used, so that the matched atoms can be deleted before searching for the next maximum common substructure. Maximum common substructures are found using the function FindMCS from the rdkit.Chem.rdFMCSmodule with the setting bondCompare=rdFMCS.BondCompare.CompareAny, so that only the connectivity of the SMILES string is taken into account. This entire procedure results in a Mol object and the function GetSubstructMatch is used to get the list of atom indices that matches the

MCS. Using this list of indices, the atom map numbering of the TS-derived Mol object is set to correspond to the numbering of the SMILES-derived Mol object. Notably, the list of indices is referred to the truncated Mol objects, therefore the previously negative atom map number are used to find the corresponding atom between the truncated Mol objects and the TS Mol object. After the atom map numbers are matched, the bonding information and charge information is taken from the SMILES Mol object and copied to the TS Mol object. Sanitization (via `rdkit.Chem.SanitizeMol`) and stereochemistry assignment (via `rdkit.Chem.AssignStereochemistryFrom3D`) are then performed on this Mol object for further handling.

Regardless which `molgetter` module is used, the frozen atoms are determined based on their connectivity to the reaction center atoms. As explained above, as frozen atoms we count the reaction center atoms and their directly connected neighbors.

`embedder`

Once the Mol object is retrieved, conformer generation is performed in the `embedder` module via sampling from a constrained distance matrix. racer^{TS} provides two different options in the embedder module: (1) the `CmapEmbedder`, which uses a coordination map of the frozen atoms to provide position constraints[1], and (2) the `BoundMatrixEmbedder`, which introduces the constraints by directly modifying the bounds matrix and additional triangle smoothing of the bounds matrix. As the default within racer^{TS} , we chose the `CmapEmbedder`.

Within `CmapEmbedder`, a coordination map (a mapping between atoms in the conformer and the reference conformer) is used to provide the necessary constraints. The coordination map is generated by providing reference 3D coordinates of every frozen atom, which is taken from the user-provided template transition state structure. This coordination map is then passed as an argument to the `rdkit.AllChem.EmbedMultipleConfs` function. The default settings for the `AllChem.EmbedMultipleConfs` function are `useMacrocycleTorsions=True`, to set `useSmallRingTorsions=True`, set `embedFragmentsSeparately=False`, to use

ETversion=2, and to not prune conformers at this stage, *i.e.*, to set pruneRmsThresh to -1, as pruning is performed later on force-field optimized structures. Additionally, the default setting within racer^{TS} is to set the flag useRandomCoords=True, which uses a random coordinate embedding to generate the initial conformers and has been demonstrated to provide better alignment of constrained regions[1]. The randomSeed=12 is used by default, and the number of requested conformers per default is number_of_conformers $=$ num_rot_bonds $\cdot$ conf_factor $+ 30$[2], where num_rot_bonds is the number of rotatable bonds of the retrieved Mol object and conf_factor is a user-input which defaults to 30. The additive term ensures that regardless of molecule size, at least 30 conformers are generated. As discussed in the main text, conf_factor can easily be tuned for a more comprehensive coverage of conformer space, and alternatively, a fixed number of conformers can also be set by the user. As an initial step, using the rdkit.AllChem.EmbedMultipleConfs function, n conformers (n is a user-definable parameter, $n \geq 3$) are generated and checked for chiral errors. If such errors are detected, chiral tags are either set to UNSPECIFIED or we set enforceChirality=False to ensure that conformers are generated, as appropriate for the detected error[3]. In the case of neglecting chirality in the generation, a warning message is printed to the user. The remaining requested number_of_conformers - n conformers are subsequently generated with the potentially updated settings.

In the BoundMatrixEmbedder module, the bounds matrix is modified such that the lower and upper distance bounds for each combination of frozen atoms are exactly the distances in the user-provided structure. As explained in the main text, as frozen atom we count every reaction center and their directly connected neighbors. The initial bounds matrix without constraints is generated via the rdkit.AllChem.GetMoleculeBoundsMatrix function. The bounds matrix is first modified according to the distances in the transition state template by manually replacing the elements, then further refined via triangle smoothing, modifying the bounds to adhere to the triangle inequality[4]. Notably, we empirically observe that the integration of a linearly increasing tolerance factor (tol) as a keyword in the rdkit.Dist

anceGeometry.DoTriangleSmoothing function is necessary for the triangle smoothing to succeed, most likely due to the manual modification of the bounds matrix. The modified bounds matrix is then passed to the rdkit.AllChem.EmbedMultipleConfs function to generate the conformers. All other settings as well as chirality error handling are as described above for the CmapEmbedder.

optimizer

After conformer generation, each conformer is refined with a force field, as is common practice[5,6]. racer^{TS} has two refinement methods implemented: MMFFOptimizer and UFFOptimizer, which optimize using the MMFF94[7] or the UFF[8] force field, respectively. For both options, the positions of the frozen atoms are fixed to respect the distances for reaction centers. The default within racer^{TS} is an optimization via the MMFFOptimizer. MMFF94 is only parametrized for organic molecules, not recognizing atom types for transition metal atoms. Additionally, atom types are sometimes not recognized for reaction center atoms due to their uncharacteristic connectivity. In case refinement with MMFFOptimizer is unsuccessful due to these reasons, the UFFOptimizer is used, which assigns all unrecognized atom types a default value, thus enabling refinement, albeit with lower accuracy.

pruner

In the last step, redundant conformers are pruned from the ensemble. Redundant conformers are either duplicates of already existing conformers or significantly higher in energy compared to the lowest identified energy conformer. racer^{TS} contains two pruner modules, which are applied in consecutive order: EnergyPruner, which removes high-energy conformers, and RMSDPruner, which removes duplicate conformers.

In EnergyPruner, the energy of each conformer is taken from the force field refinement in the previous step. As a non-default option, we offer the flag YAeHMOP_energies, which calculates the energy according to the extended Hückel theory based on the YAeHMOP library

interfaced in the `rdkit.Chem.rdEHTTools` module. All conformers with an energy higher than a defined threshold (default is 20 kcal/mol) compared to the most stable conformer are then removed from the ensemble.

In the `RMSDPruner` module, duplicate conformers are removed based on their RMSD. Two conformers are considered as duplicates if their energetic difference is below 0.1 kcal/mol, their principal moments of inertia are less than 8% different, and their RMSD is below 0.125. If two conformers are judged to be identical, the conformer with the higher energy is removed. RMSD calculations can be set to include hydrogen atoms and include frozen atoms, both of which are not included in the default settings of racer^{TS}.

Benchmarking reactions

To assess the performance of racer^{TS} and compare it to state-of-the-art conformer generators CREST and GOAT, we generate transition state conformer ensembles for 20 benchmarking reactions. The transition states are taken from different sources (see below), re-optimized at the r^{2}SCAN-3c level of theroy[9] until a transition state is identified that connects the appropriate reactants and products, as judged via the Quick Reaction Coordinate (QRC) procedure[10,11]. The benchmarking reactions are shown in Figures S1, S2, S3, and S4. The reactions were manually selected from the literature to broadly cover reaction space, and include 16 organic reactions and four organometallic reactions. Furthermore, positively, and negatively charged transition states, as well as transition states involving carbenes are also included. For a better overview, we here list the reaction types and the sources from which we took the original transition states: amide methylation[12], intramolecular cyclization (referred to as cyclization)[13], epoxidation[14], hydride transfer[15], hydroborylation[15], addition of a nucleophile to an NHC (referred to as NHC)[16], intramolecular nucleophilic addition to an iminium cation (referred to as nucleophilic addition)[15], cyclization of a peptide (referred to as peptide)[17,18], the first step of a step-wise pericyclic reaction (referred to as pericyclic)[15], propargylation reaction[19], intra-molecular proton transfer reaction (referred to as proton

transfer)[15], intramolecular epoxide formation via rearrangement (referred to as epoxide rearrangement)[20], S_N2-like addition elimination reaction (referred to as S_N2)[21], S_N2 reaction on a sugar molecule (referred to as S_N2 sugar)[15], S_NAr[22], nucleophilic addition of CH_3I to tropane (referred to as tropane alkylation)[23], Pd-catalyzed carbofluorination (referred to as Pd carbofluorination)[24], Pd oxidative addition (referred to as Pd oxidative addition)[25], Ru-catalyzed olefin metathesis (referred to as Ru olefin metathesis)[26], and Ti-catalyzed reductive elimination (referred to as Ti elimination)[27].

Computational details of conformer generation for CREST and GOAT

To generate conformers with CREST, version 2.12 was used. Even though CREST version 3.0 was already available we picked the previous version as the computational level used in this study (optimization with GFN-FF[28], single-point energy calculation with GFN2-xTB[29]) was not available in version 3.0 at the time of calculation. Used settings include the `--noreftopo` flag, which disables the initial topology check prior to conformer search, and the `subrmsd` flag, which only considers the atoms not constrained to calculate the RMSD for pruning. The distances between all frozen atoms were constrained to the distance of the user-provided input structure, with a force constant of 1.5.

GOAT conformer searches were run with ORCA version 6.0.1[30,31]. Similarly to the CREST search, all distances between frozen atoms were fixed to those in the user-provided template structure that was used for the GOAT run. Additionally, atom positions of the frozen atoms were also constrained to further ensure conservation of the reaction center. As GOAT settings, the `randomseed` was set to `False` to make the run deterministic, and `autofrag` was set to `False` to avoid initial fragment detection which might be problematic due to the presence of reaction centers. The GOAT conformer search was performed at the GFN2-xTB level, with GFN-FF used for the uphill steps within GOAT.

A further sub-variant of GOAT was also tested and is discussed here in the Supporting Information: GOAT-ENTROPY, referred hereafter as ENTROPY. ENTROPY aims to

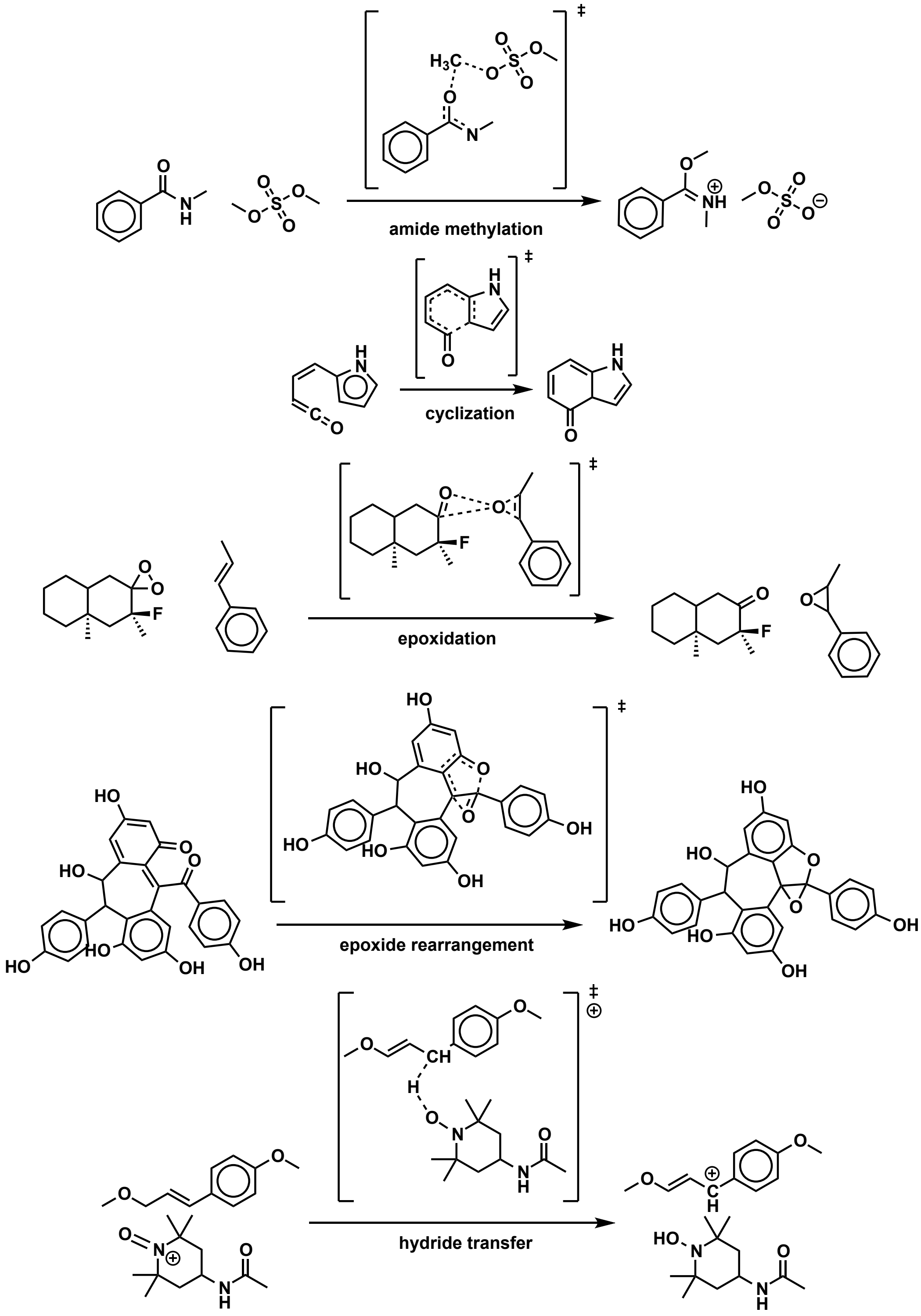

Figure S1: Organic reactions considered in the benchmarking procedure. Further reactions are shown in Figures S2, S3, and S4.

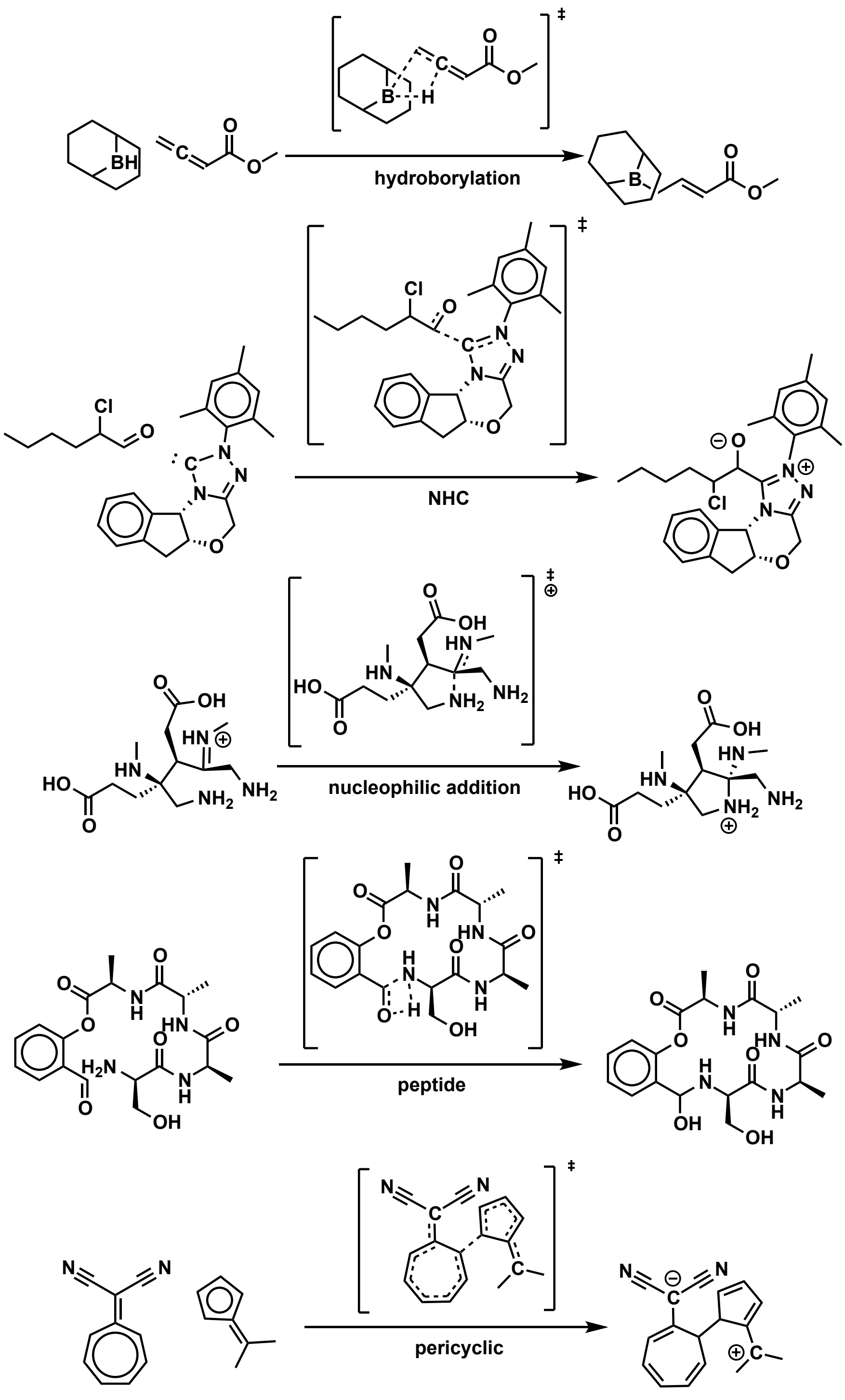

Figure S2: Organic reactions considered in the benchmarking procedure. Further reactions are shown in Figures S1, S3, and S4.

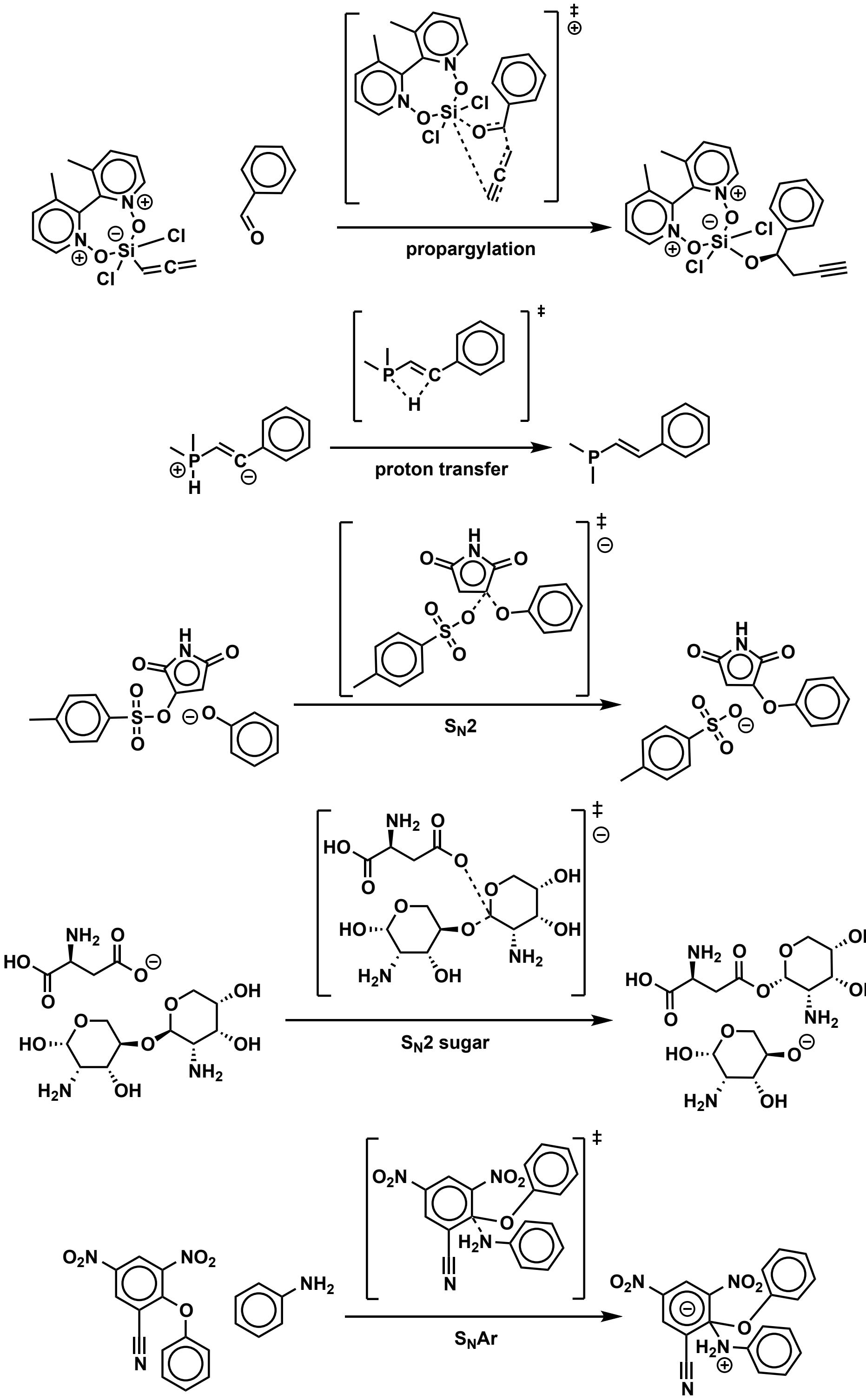

Figure S3: Organic reactions considered in the benchmarking procedure. Further reactions are shown in Figures S1, S2, and S4.

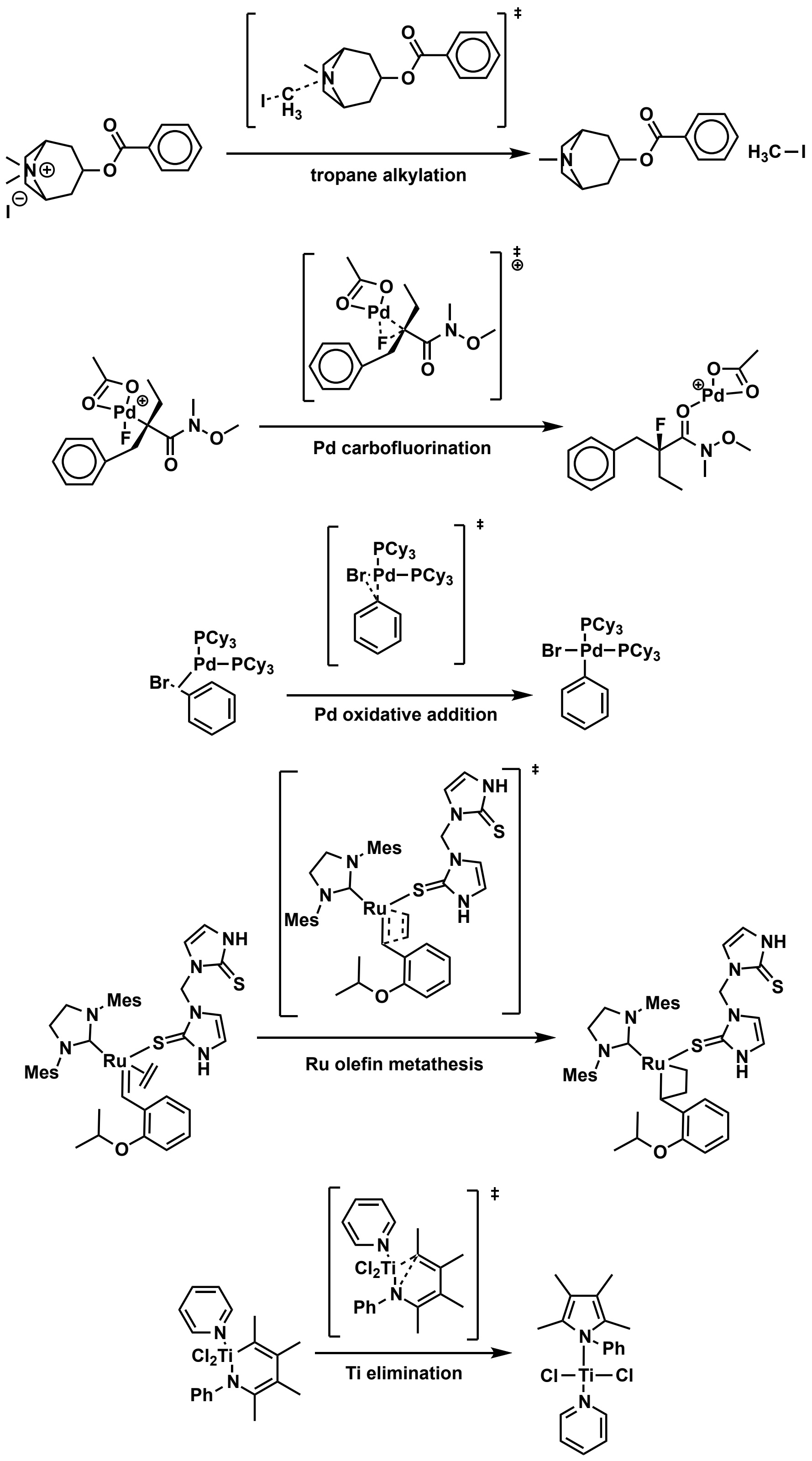

Figure S4: Organic and organometallic reactions considered in the benchmarking procedure. Further reactions are shown in Figures S1, S2, and S3.

achieve a higher ensemble completeness, at additional computational cost, by only terminating when the conformational entropy has converged. All other settings were the same as for GOAT conformer searches.

Additional benchmarked variations of racer$^{\mathbf{TS}}$

In addition to the racer^{TS} variants discussed in the main text, two further options were tested. Their performance is discussed below. The two methods are: i) **racer$^{\mathbf{TS}}$ SMILES (bounds)** , which has the same setting as **racer$^{\mathbf{TS}}$ SMILES** , but uses the `BoundMatrixEmbedder` in the embedder module, and ii) **racer$^{\mathbf{TS}}$ (minimal)** , which has the least expected accurate settings for every module, *i.e.*, uses the `MolGetterConnectivity` to get the Mol object and only refine conformers using the `UFFOptimizer`. **racer$^{\mathbf{TS}}$ SMILES (bounds)** shows the influence on the results by changing the `embedder`, while **racer$^{\mathbf{TS}}$ (minimal)** shows the 'worst case' results that racer^{TS} is expected to return. Notably, **racer$^{\mathbf{TS}}$ SMILES (bounds)** uses an embedding methodology that is similar to the one used in the AutoTST package from West and co-workers[32]. AutoTST shows several differences: (1) a less robust method to embed conformers which produces fewer valid TS structures for larger molecules is used (via the bounds matrix, see results below), (2) only conformer optimization via the universal force field (UFF[8]) is enabled, (3) critical pruning tools for ensemble generation, instead of finding only the lowest energy conformer, are not provided, and (4) reactive center distances are generated via group contributions instead of an input structure. Next to the additional racer^{TS} variants, the Supporting Information also compares benchmark results for the ENTROPY variant of GOAT discussed above. An overview of the computational details of all compared methods is given in Table S1, while the modules as described above of all tested racer^{TS} variants are described in Table S2.

Table S1: Overview of the input and post-optimization methods discussed in the Supporting Information.

Name	Method	Input	GFN2-xTB//GFN-FF post-optimization
**racer$^{\mathbf{TS}}$**	racer^{TS}	3D structure, reaction centers, charge	yes
**racer$^{\mathbf{TS}}$ SMILES**	racer^{TS}	3D structure, reaction centers, reactant SMILES, charge	yes
**racer$^{\mathbf{TS}}$ (no xTB)**	racer^{TS}	3D structure, reaction centers, charge	no
**CREST**	CREST	3D structure, reaction centers, charge	no
**GOAT**	GOAT	3D structure, reaction centers, charge	no
**racer$^{\mathbf{TS}}$ SMILES (bounds)**	racer^{TS}	3D structure, reaction centers, reactant SMILES, charge	yes
**racer$^{\mathbf{TS}}$ (minimal)**	racer^{TS}	3D structure, reaction centers, charge	yes
**ENTROPY**	GOAT-ENTROPY	3D structure, reaction centers, charge	no

Table S2: Description of the modules used for each racer^{TS} variant.

Name	`molgetter`	`embedder`	`optimizer`	`pruner`
**racer^{TS}**	`MolGetterBonds`, if that fails `MolGetterConnectivity`	`CmapEmbedder`	`MMFFOptimizer`, if that fails `UFFOptimizer`	`EnergyPruner` + `RMSDPruner`
**racer^{TS} SMILES**	`MolGetterSMILES`, if that fails `MolGetterBonds`, if that fails `MolGetterConnectivity`	`CmapEmbedder`	`MMFFOptimizer`, if that fails `UFFOptimizer`	`EnergyPruner` + `RMSDPruner`
**racer^{TS} (no xTB)**	`MolGetterBonds`, if that fails `MolGetterConnectivity`	`CmapEmbedder`	`MMFFOptimizer`, if that fails `UFFOptimizer`	`EnergyPruner` + `RMSDPruner`
**racer^{TS} SMILES (bounds)**	`MolGetterSMILES`, if that fails `MolGetterBonds`, if that fails `MolGetterConnectivity`	`BoundMatrixEmbedder`	`MMFFOptimizer`, if that fails `UFFOptimizer`	`EnergyPruner` + `RMSDPruner`
**racer^{TS} (minimal)**	`MolGetterConnectivity`	`CmapEmbedder`	`UFFOptimizer`	`EnergyPruner` + `RMSDPruner`

Additional results

In addition to the methods discussed in the main text, the benchmarking of other variants of racer^{TS} and ENTROPY is described in this section. Additional investigations as the ones referenced in the main text are described here and the results of all benchmarked reactions are summarized here.

Computational cost

As the first metric, we consider the computational cost of each conformer generator (Equation 1, as shown in Figure S5). As expected, **racer^{TS} SMILES (bounds)** shows a highly similar runtime to **racer^{TS} SMILES**, including xTB post-optimization. Surprisingly, **racer^{TS} (minimal)** significantly exceeds this runtime. As explained previously, the `MolGetterConnectivity` used within **racer^{TS} (minimal)** does not properly recognize aromatic or conjugated systems. The added flexibility leads to more initial conformers generated in the `embedder` module within racer^{TS} (see also Table S4). These conformers are then optimized in the post-optimization stage, and the additionally generated conformers lead to more time-consuming optimizations. Although **racer^{TS} (minimal)** is by far the slowest variant of racer^{TS}, it still provides a 3x speed-up on average over the most rapid state-of-the-art method CREST, again underlining the reduction in computational cost.

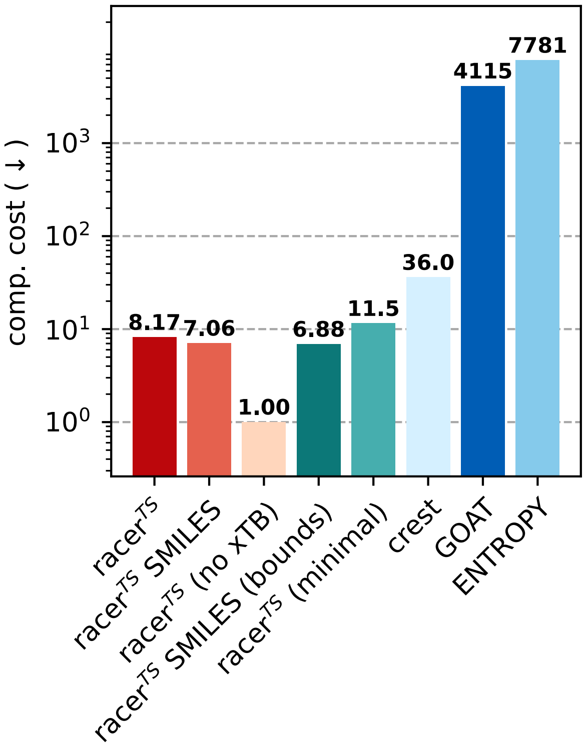

Figure S5: Average runtime of conformer ensemble generators, normalized to the runtime of **racer$^{\mathbf{TS}}$ (no xTB)** (Equation 1).

Table S3: Runtime for each benchmarked conformer generator to generate the TS conformer ensembles for each reaction on one CPU. For variants of racer^{TS} , the runtime fraction of the xTB post-optimization is provided in brackets.

reaction	**racer^{TS}** / s	**racer^{TS} SMILES** / s	**racer^{TS} (no xTB)** / s	**racer^{TS} SMILES (bounds)** / s	**racer^{TS} (minimal)** / s	**CREST** / s	**GOAT** / s	**ENTROPY** /s
amide methylation	29 (77%)	33 (80%)	7 (0%)	46 (83%)	170 (95%)	140	18239	30542
cyclization	29 (98%)	23 (98%)	1 (0%)	6 (89%)	21 (97%)	66	14602	27307
epoxidation	33 (44%)	40 (57%)	20 (0%)	50 (57%)	25 (33%)	353	87753	174589
epoxide rearrangement	96 (70%)	136 (76%)	29 (0%)	176 (77%)	133 (77%)	490	23643	42388
hydride transfer	149 (56%)	327 (77%)	66 (0%)	507 (83%)	898 (91%)	452	76566	123278
hydroborylation	19 (81%)	13 (60%)	4 (0%)	34 (82%)	37 (82%)	308	42661	69756
NHC	359 (83%)	418 (85%)	60 (0%)	422 (83%)	662 (91%)	1688	114362	252428
nucleophilic addition	233 (89%)	236 (89%)	27 (0%)	319 (89%)	424 (94%)	505	51063	117869
peptide	208 (87%)	233 (87%)	28 (0%)	98 (65%)	337 (93%)	592	76566	113910
pericyclic	88 (95%)	86 (93%)	5 (0%)	137 (95%)	141 (95%)	130	14259	26046
propargylation	87 (71%)	87 (71%)	25 (0%)	158 (76%)	151 (83%)	447	21518	40596
proton transfer	39 (94%)	9 (84%)	2 (0%)	18 (91%)	20 (90%)	212	6298	14508
S_N2	12 (29%)	10 (52%)	8 (0%)	19 (66%)	74 (85%)	507	33799	55699
S_N2 sugar	288 (81%)	362 (68%)	56 (0%)	324 (63%)	856 (94%)	1055	111591	429097
S_NAr	55 (77%)	18 (45%)	13 (0%)	61 (73%)	91 (88%)	308	14049	27073
tropane alkylation	29 (73%)	18 (61%)	8 (0%)	33 (72%)	32 (76%)	541	48280	101560
Pd carbofluorination	147 (74%)	100 (85%)	39 (0%)	90 (83%)	153 (75%)	548	47665	73950
Pd oxidative addition	345 (12%)	410 (28%)	305 (0%)	314 (36%)	339 (10%)	1390	90086	244046
Ru olefin metathesis	1805 (70%)	2471 (78%)	540 (0%)	1696 (73%)	1834 (71%)	3174	412341	175595
Ti elimination	77 (50%)	56 (25%)	39 (0%)	78 (24%)	81 (52%)	1020	30504	52640

Exhaustiveness

Similarly to the discussion presented in the main manuscript, **CREST** produces the highest number of conformers for a majority of the benchmarked reactions (16/20) (see Table S4). Comparing the number of conformers between **racer$^{\mathbf{TS}}$ SMILES (bounds)** and **racer$^{\mathbf{TS}}$ SMILES** shows that the amount of generated conformers is approximately the same throughout all reactions. These results are an early indication that both `embedder` modules, `BoundMatrixEmbedder` and `CmapEmbedder`, are suitable for generating conformers, although more detailed results are discussed below. In contrast, **racer$^{\mathbf{TS}}$ (minimal)** produces the lowest number of conformers for 10/20 reactions. A closer investigation reveals that the majority of conformers from **racer$^{\mathbf{TS}}$ (minimal)** are removed after GFN2-xTB//GFN-FF post-optimization. This observation indicates that **racer$^{\mathbf{TS}}$ (minimal)** produces few conformers that are low-energy at the GFN2-xTB//GFN-FF level. We hypothesize that this behaviour can be explained by the missing bonding information when the molecular topology is inferred, leading to many high energy conformers (*e.g.*, aromatic rings are not recognized as aromatic, and thus non-planar conformers are produced). These conformers are then pruned when the stricter energy window is applied after GFN2-xTB//GFN-FF optimization. Notably, **ENTROPY** , a variant of **GOAT** that emphasizes sampling of the entire conformational space, produces the same number of conformers as **GOAT** for all reactions, indicating that already the standard variant of **GOAT** samples the space exhaustively for these reactions, according to their internal conformational entropy metric. A particularly interesting case for **GOAT** and **ENTROPY** is the S_N2 sugar reaction, where these methods produce the highest number of conformers. We suspect that this is because the other methods, in particular variants of racer^{TS} , lose many conformers due to pruning of high energy conformers, where mostly non-chair structures are produced for the ring systems, as discussed in the main text.

Investigating the exhaustiveness of all methods, we again consider the space exploration metric (Equation 4). The results of all methods are given in Figure S6. Similarly to the

observations discussed in the main text, the variations of racer^{TS} cover the space similarly to **CREST** , while **GOAT** and **ENTROPY** cover the space most exhaustively. For both **racer^{TS} SMILES (bounds)** and **racer^{TS} (minimal)** , the average precision is close to 1, indicating that almost all generated conformers belong to the (combined) ground truth ensemble. As the precision is close to 1, the space exploration metric is again determined by the recall. **racer^{TS} SMILES (bounds)** performs similarly to **racer^{TS} SMILES** for this metric, indicating that both `embedder` modules are suitable for sampling the conformer space. In contrast, **racer^{TS} (minimal)** demonstrates the lowest recall, which is in alignment with this method producing the lowest number of conformers for almost half of benchmarked reactions.

Considering the space distribution metric (Equation 5), one notices that both **racer^{TS} SMILES (bounds)** and **racer^{TS} (minimal)** perform similarly to the racer^{TS} variants discussed in the main text (see Figure S6). Consistent with results discussed in the main text, **GOAT** and **ENTROPY** cover the space most exhaustively as indicated by a low space distribution metric.

Considering the two metrics, it can be stated that, in agreement with the conclusions in the main text, racer^{TS} explores the space exhaustively and finds similarly or even more diverse conformers than state-of-the-art methods. The results discussed herein show that the `embedder` module does not significantly impact the exhaustiveness of the explored conformer space. However, using **racer^{TS} (minimal)** tends to generate a low number of conformers, lowering the extent to which the conformer space is covered.

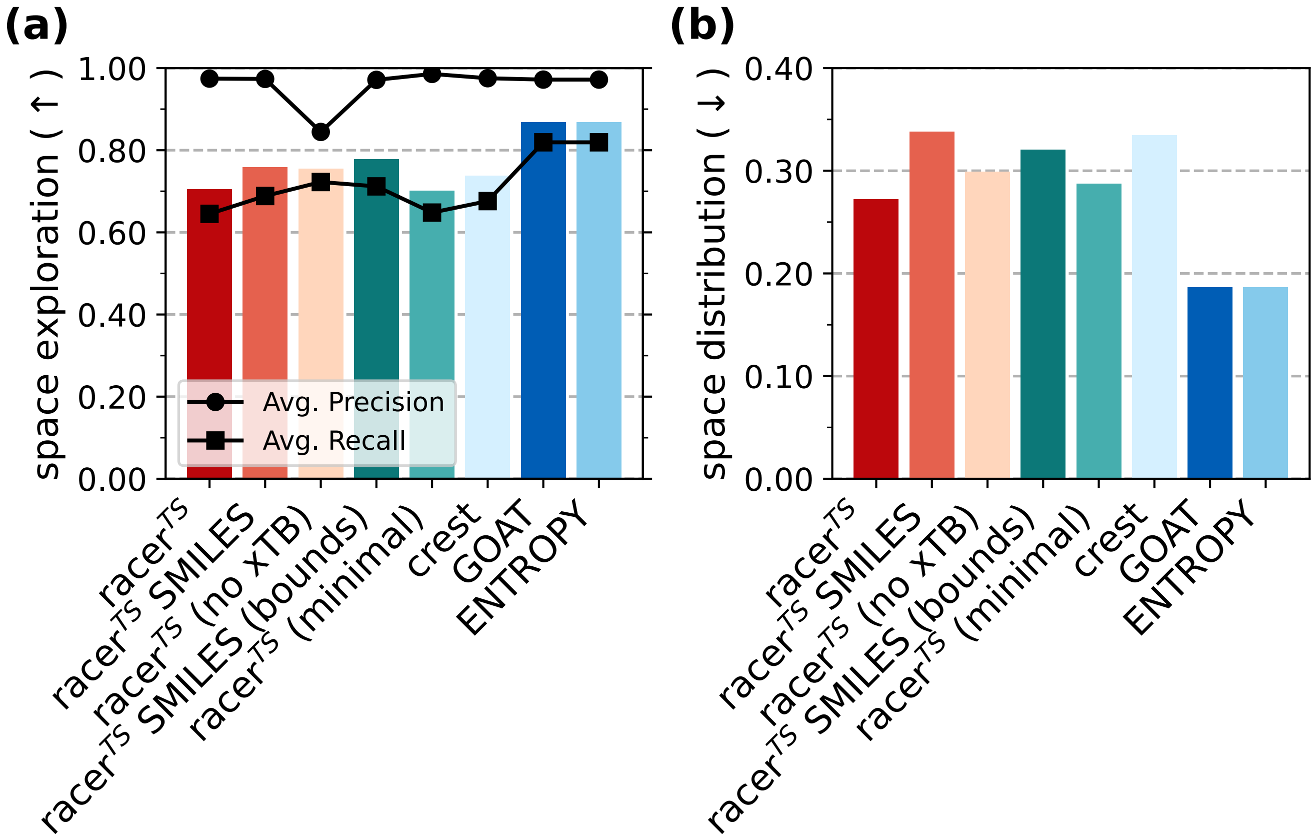

Figure S6: Comparison of exhaustiveness metrics for the five benchmarked conformer generators. **Left:** Space exploration as determined by Equation 4 for each method. **Right:** Space distribution as determined by Equation 5 for each method.

Table S4: Number of conformers generated per method for each investigated reaction. The highest number of conformers is marked in bold, while the lowest number is underlined.

reaction	**racer^{TS}**	**racer^{TS} SMILES**	**racer^{TS} (no xTB)**	**racer^{TS} SMILES (bounds)**	**racer^{TS} (minimal)**	**CREST**	**GOAT**	**ENTROPY**
amide methylation	10	11	16	17	1	**119**	34	34
cyclization	17	19	33	6	4	**34**	1	1
epoxidation	4	10	9	2	4	**426**	10	10
epoxide rearrangement	28	39	37	46	23	**53**	48	48
hydride transfer	21	26	40	28	13	**228**	47	47
hydroborylation	7	5	15	7	10	**104**	24	24
NHC	93	70	115	59	12	**1426**	215	215
nucleophilic addition	35	57	143	30	30	**264**	192	192
peptide	2	3	**97**	9	3	86	21	21
pericyclic	24	23	**48**	36	25	6	3	3
propargylation	9	9	31	11	13	**232**	3	3
proton transfer	11	5	39	15	7	**740**	1	1
S_N2	2	4	2	6	1	**181**	9	9
S_N2 sugar	2	19	106	2	2	14	**182**	**182**
S_NAr	14	4	23	17	32	**214**	1	1
tropane alkylation	5	7	16	9	5	**806**	7	7
Pd carbofluorination	11	3	**55**	5	11	17	7	7
Pd oxidative addition	2	2	8	6	2	**111**	71	71
Ru olefin metathesis	14	57	173	34	14	**408**	181	181
Ti elimination	7	6	21	8	7	**266**	4	4

Validity

Comparing the success rate (Equation 6) among the different methods shows that **racer$^{\mathbf{TS}}$ SMILES (bounds)** and **racer$^{\mathbf{TS}}$ (minimal)** generate valid TS structures with a similar rate as the state-of-the-art methods **CREST** and **GOAT**, albeit slightly lower than the racer^{TS} variants discussed in the main text (see Figure S7). This increased error rate results in reactions for which no valid TS could be identified with the respective methods. For **racer$^{\mathbf{TS}}$ SMILES (bounds)**, no valid reaction could be generated for the NHC reaction, for **racer$^{\mathbf{TS}}$ (minimal)** no valid TS was identified for the NHC and S_N2 reactions. Investigating the failure modes for the NHC reaction shows that the DFT TS optimizations did not converge in all cases for the two methods. This particular reaction proved tricky for almost all methods, as optimizations failed either by reaching the maximum number of steps in TS optimization or failed to connect reactants and products. For the S_N2 reaction with **racer$^{\mathbf{TS}}$ (minimal)**, constrained geometry optimization did not converge, as the input structure was not sensible, since only the connectivity has been assigned, leading to unconventional bonding scenarios in the produced conformer. Comparing **GOAT** and **ENTROPY** shows that **ENTROPY** finds a valid TS for one additional reaction, the hydroborylation reaction. A common error mode for this reaction was the coordination of the carbonyl-oxygen to the boron atom, which was considered as an unsuccessful QRC (products had to be separated). While this coordination process might correspond to a barrierless relaxation to real minimum after crossing the TS, treating this as an unsuccessful TS optimization does not put any of the benchmarked methods at a particular disadvantage.

Overall, all benchmarked methods perform on an adequate level and produce error rates consistent with literature reports on high-throughput computations[33–36]. Therefore, we conclude that all variants of racer^{TS} are suited for use in high-throughput computational chemistry pipelines.

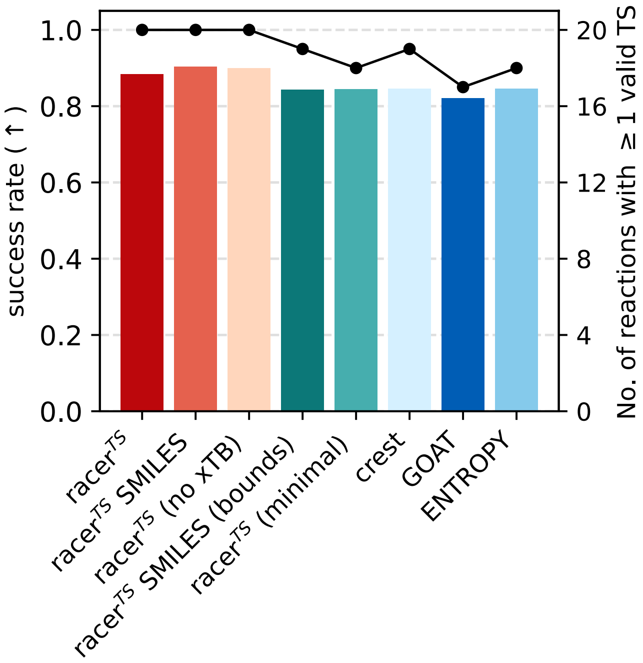

Figure S7: Success rate of conformers producing correct transition states in the benchmarking DFT pipeline (Equation 6, left). For each method, conformers are selected and transition states are optimized with DFT. The fraction of DFT transition states connecting the desired reactants and products is assessed, averaged over all reactions. The black dots show the number of benchmarked reactions with at least one successfully converged conformer.

Accurate low-energy regions

Similarly to the main text, the identification of the lowest-energy conformers is investigated for the additionally benchmarked methods and the results are shown in Figure S8 and in Table S5. Considering **racer$^{\mathbf{TS}}$ SMILES (bounds)** , we notice an accuracy comparable to the state-of-the-art for discovering the lowest energy conformers. For top-1 accuracy (Equation 7), the lowest energy conformer is successfully identified for the cyclization, epoxidation, and pericyclic reactions, all reactions involving ring systems. Considering top-5 accuracy (Equation 8), the lowest energy conformers are additionally discovered for the Ru olefin metathesis, propargylation and tropane alkylation reaction. The diversity of reactions for which **racer$^{\mathbf{TS}}$ SMILES (bounds)** successfully discovers the lowest energy conformer shows its broad utility. All reactions for which the lowest energy conformers are correctly identified are shown in Table S5. Investigating **racer$^{\mathbf{TS}}$ (minimal)** , the lowest energy conformer is successfully identified for the cyclization, pericyclic, propargylation,and proton transfer reaction (top-1), and additionally for the Ti elimination and the S_NAr (top-5). All methods correctly identify the lowest-energy conformer for the cyclization and pericyclic reaction due to the low flexibility of the TS. However, the diversity of reactions for which **racer$^{\mathbf{TS}}$ (minimal)** identifies the lowest-energy conformer, again demonstrates that all racer^{TS} variants are in principle able to provide accurate low-energy conformers for a large variety of reactions. Similarly to the conclusions drawn in the main text, we identify the biggest bottleneck in energy-ranking based on the GFN2-xTB//GFN-FF level. Therefore, optimizing all conformers with DFT or similarly accurate methods[37,38] will significantly increase the chances of identifying the lowest-energy conformer. However, such an approach can become unfeasible due to the associated computational cost. As an alternative, our conclusions show that all racer^{TS} variants provide ensembles that yield the lowest energy conformer with an accuracy that matches or exceeds state-of-the-art.

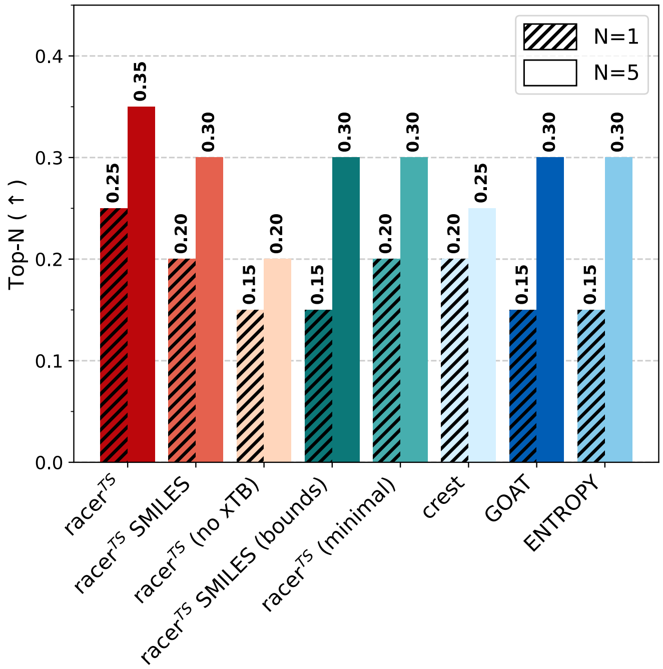

Figure S8: Top-1 (hatched) (Equation 7) and top-5 (Equation 8) accuracy in identifying the overall lowest energy conformer.

Table S5: Indication if the lowest energy conformer is correctly identified by each method. x marks that the lowest energy conformer is assessed as the lowest energy conformer by the method (i.e., top-1 accuracy, (Equation 7)), while the o marks that the lowest energy conformer is among the five lowest energy conformers for each method (i.e., top-5 accuracy, (Equation 8)).

reaction	**racer$^{\mathbf{TS}}$**	**racer$^{\mathbf{TS}}$ SMILES**	**racer$^{\mathbf{TS}}$ (no xTB)**	**racer$^{\mathbf{TS}}$ SMILES (bounds)**	**racer$^{\mathbf{TS}}$ (minimal)**	**CREST**	**GOAT**	**ENTROPY**
amide methylation	-	-	-	-	-	-	o	o
cyclization	x	x	x	x	x	x	x	x
epoxidation	-	-	-	x	-	-	-	-
epoxide rearrangement	x	-	-	-	-	-	-	-
hydride transfer	-	-	-	-	-	-	-	-
hydroborylation	-	-	-	-	-	-	-	-
NHC	-	-	-	-	-	-	-	-
nucleophilic addition	-	-	-	-	-	x	o	o
peptide	-	-	-	-	-	o	-	-
pericyclic	x	x	x	x	x	x	x	x
propargylation	x	x	o	o	x	x	o	o
proton transfer	o	o	x	-	x	-	-	-
S_N2	-	-	-	-	-	-	x	x
S_N2 sugar	-	-	-	-	-	-	-	-
S_NAr	x	-	-	-	o	-	-	-
tropane alkylation	-	-	-	o	-	-	-	-
Pd carbofluorination	-	x	-	-	-	-	-	-
Pd oxidative addition	-	-	-	-	-	-	-	-
Ru olefin metathesis	-	-	-	o	-	-	-	-
Ti elimination	o	o	-	-	o	-	-	-

Besides testing the capabilities in identifying the lowest energy conformer, we also test their suitability to calculate activation energies $\Delta E^{\ddagger}_{\mathrm{lowe}}$ (Equation 9) and $\Delta E^{\ddagger}_{\mathrm{marc}}$ (Equation 10). As indicated in Figure S9, **racer$^{\mathbf{TS}}$ SMILES (bounds)** is able to provide accurate estimations of the activation energy for both $\Delta E^{\ddagger}_{\mathrm{lowe}}$ and $\Delta E^{\ddagger}_{\mathrm{marc}}$. This observation is consistent with its ability to identify low-energy conformers (see Figure S8). Similarly, **racer$^{\mathbf{TS}}$ (minimal)** also provides accurate activation energy estimates for $\Delta E^{\ddagger}_{\mathrm{marc}}$. In contrast, when calculating activation energies solely based on the lowest-energy conformer, this method leads to slightly higher errors. However, with the median deviation being significantly below chemical accuracy, we again demonstrate that all racer^{TS} variants are suitable for calculating activation energies with reasonable accuracy in a majority of cases. Investigating the shown outliers (in Figure S9) more closely, we find that outliers for **racer$^{\mathbf{TS}}$ SMILES (bounds)** and **racer$^{\mathbf{TS}}$ (minimal)** are consistent with the discussion on outliers from the main text.

Additional investigations in conservation of reaction center distances

To investigate the reaction center preservation for each conformer generator, we investigate the difference in interatomic distances $< 2\,\text{Å}$ between atoms in the reaction center. For each of these atom pairs, we calculate the distance using the input 3D structure as well as using each generated conformer. The distribution of distance deviations across all reactions is shown in Figure S10. **CREST** only allows for constraining of atoms instead of absolute fixing, leading to a small deviation in atom center distances, while other methods have virtually no deviations w.r.t. the input 3D structure. As the mean deviation for **CREST** is only 0.01 Å, the reaction center remains well conserved for all conformer generators. The close structural alignment of the reaction center between the ground-truth TS conformer and the generated conformers is plausibly the reason for the low error rates in the TS optimization.

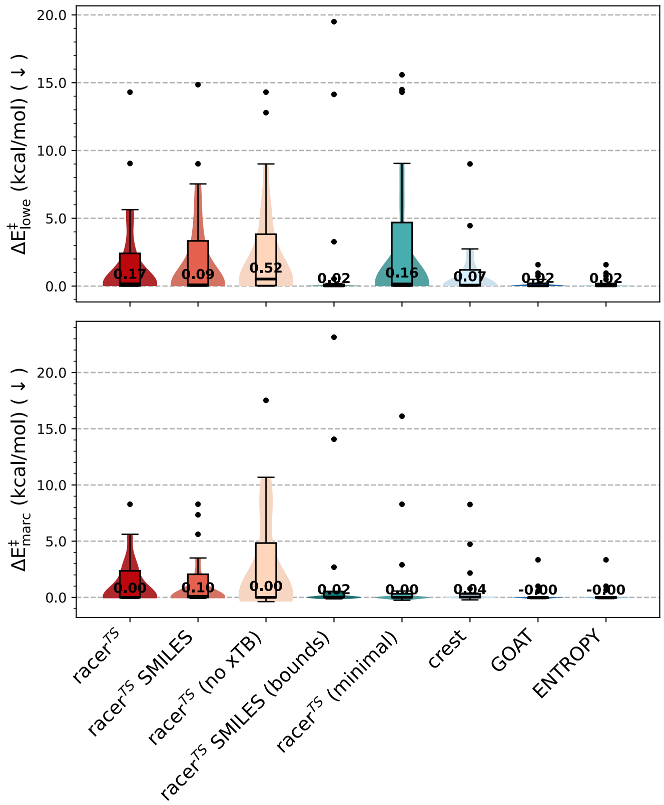

Figure S9: (Top): Tukey boxplot of $\Delta E^{\ddagger}_{\mathrm{lowe}}$ (Equation 9) for each method over all benchmarked reactions. (Bottom): Tukey boxplot of $\Delta E^{\ddagger}_{\mathrm{marc}}$ (Equation 10) for each method over all benchmarked reactions. The numerical value of the median is provided for all methods.

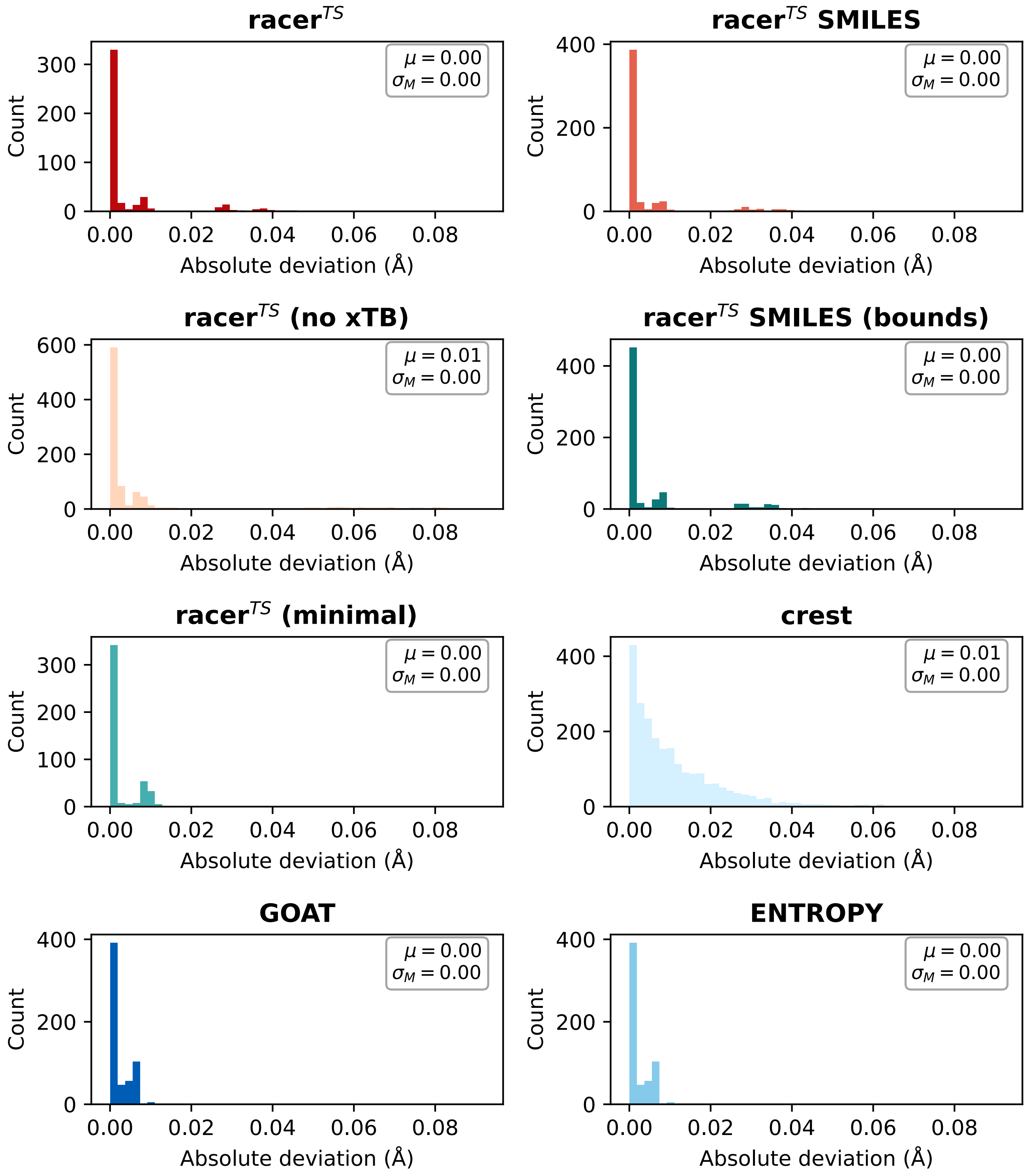

Figure S10: Distribution of deviations of interatomic distances between input 3D structure and all generated conformers for each conformer generator. Only distances $< 2\,\text{Å}$ are considered.

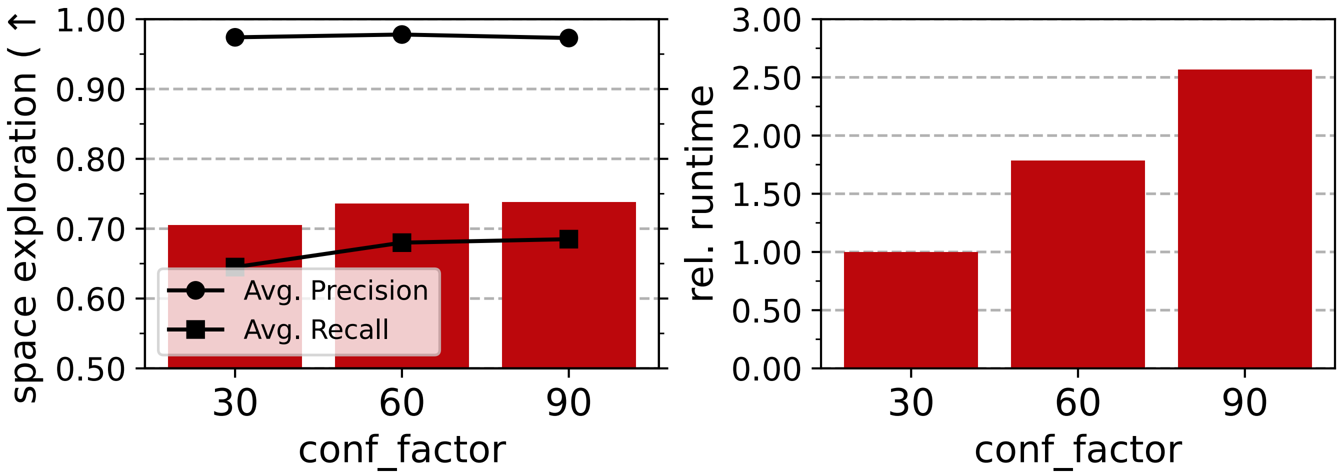

Figure S11: Comparison of the performance of **racer^{TS}** with different `conf_factor` settings. **Left:** Space exploration metric (Equation 4) for `conf_factor` set to 30, 60, and 90. **Right:** Runtime relative to **racer^{TS}** with `conf_factor` 30, for **racer^{TS}** with `conf_factor` 30, 60, and 90.

Additional investigation into `conf_factor` parameter of racer^{TS}

As explained in the main text, we integrated the `conf_factor` argument within racer^{TS}, which determines the number of initially requested conformers. To demonstrate the influence of altering `conf_factor`, we performed conformer generation with the **racer^{TS}** parameter `conf_factor` chosen as 30, 60, or 90. The setting with `conf_factor` 30 is discussed in the main text. In particular, we investigated the influence of `conf_factor` on the space exploration metric (Equation 4) and the runtime (Equation 1). Consistent with expectations, a higher `conf_factor` results in a more comprehensively explored conformer space (see Figure S11 left), albeit with diminishing returns. In addition, we also investigated the runtime associated with generating more conformers, as shown in Figure S11 right, where we notice a steady increase in runtime when generating more conformers. However, even with `conf_factor` set to 90, the average runtime is still below that of **CREST**.

Our results indicate that varying the number of requested conformers, easily doable with racer^{TS}, influences the explored conformer space and runtime. A higher `conf_factor` leads to a more comprehensively explored conformer space associated with higher computational cost. As discussed in the main text, we observe state-of-the-art space exploration

of **racer^{TS}** and significant runtime savings with `conf_factor` set to 30. As such, this was chosen as the default value.

Additional investigation into input conformer sensitivity

To investigate the sensitivity of racer^{TS} towards its input conformer, we conduct conformer generation with **racer^{TS}** on a different set of input conformers. The input conformers were obtained by taking a new conformer obtained from previous runs, which is structurally different from the used input conformer. The conformers were reoptimized on the r^{2}SCAN-3c level, and the correctness of the TS was confirmed via frequency analysis and QRC. **racer^{TS}** was run with the new input structures, and conformers analyzed according to the procedure described in the main text. Notably, for comparisons that consider ensembles from other conformer generators (e.g. identifying the overall lowest-energy conformer), the ensembles created with the original input structures were used. As can be seen from Table S6, racer^{TS} performs roughly similar across most metrics irrespective of the used input conformers. Considering exhaustiveness, similar results are achieved for both the space exploration and space distribution metrics, showing comparable performance to other racer^{TS} variants, as well as **CREST** . The observed success rates are slightly lower, which is caused by unsuccessful TS optimizations for the pericyclic reaction based on the new input conformer. Given a total of benchmarked 20 reactions, one unsuccessful reaction already lowers the success rate considerably. While this shows that high-quality conformers should still be used as an input to any conformer generator, a success rate of 80 % is still well within commonly observed rates for high-throughput TS calculations[33–36]. Notably, the biggest changes were observed in metrics concerning the accuracy in the low-energy region. While Top-1 accuracy decreased from 25 % to 10 %, Top-5 accuracy remained at 35 %. While the seemingly large variation is mainly an artifact from using only 20 benchmarked reactions, this result primarily shows that the conformers generated with the new input conformers were worse ranked. The consistent Top-5 accuracy shows that regardless of the input structure, racer^{TS} generates

low-energy conformers with a rate exceeding or matching the state-of-the-art. It also shows that the ranking of conformer energy remains the limiting factor for generating and assigning the lowest-energy conformer. **racer$^{\mathbf{TS}}$(new input)** shows a smaller median error for $\Delta E^{\ddagger}_{\mathrm{lowe}}$, while having a higher median deviation for $\Delta E^{\ddagger}_{\mathrm{marc}}$. Importantly, both of these deviations lie within chemical accuracy. Reassuringly, racer^{TS} achieves these results with a significant speed-up compared to CREST and GOAT for both sets of input conformers.

Table S6: Performance of racer^{TS} for the original and newly generated input conformers on the metrics discussed in the main text.

metric	**racer$^{\mathbf{TS}}$**	**racer$^{\mathbf{TS}}$(new input)**
comp. cost (↓)	8.2	10.0
space exploration (↑)	0.74	0.74
space distribution (↓)	0.26	0.30
success rate (↑)	0.88	0.80
Top-1 (↑)	0.25	0.10
Top-5 (↑)	0.35	0.35
Median $\Delta E^{\ddagger}_{\mathrm{lowe}}$ (↓)	0.17	0.15
Median $\Delta E^{\ddagger}_{\mathrm{marc}}$ (↓)	0.00	0.29

Detailed results for every reaction

Throughout the manuscript, results of particular reactions are discussed to emphasize the strengths and limitations of racer^{TS} and other benchmarked methods. To support these discussions, in this section we provide a summary of the investigated benchmarks for each reaction individually.

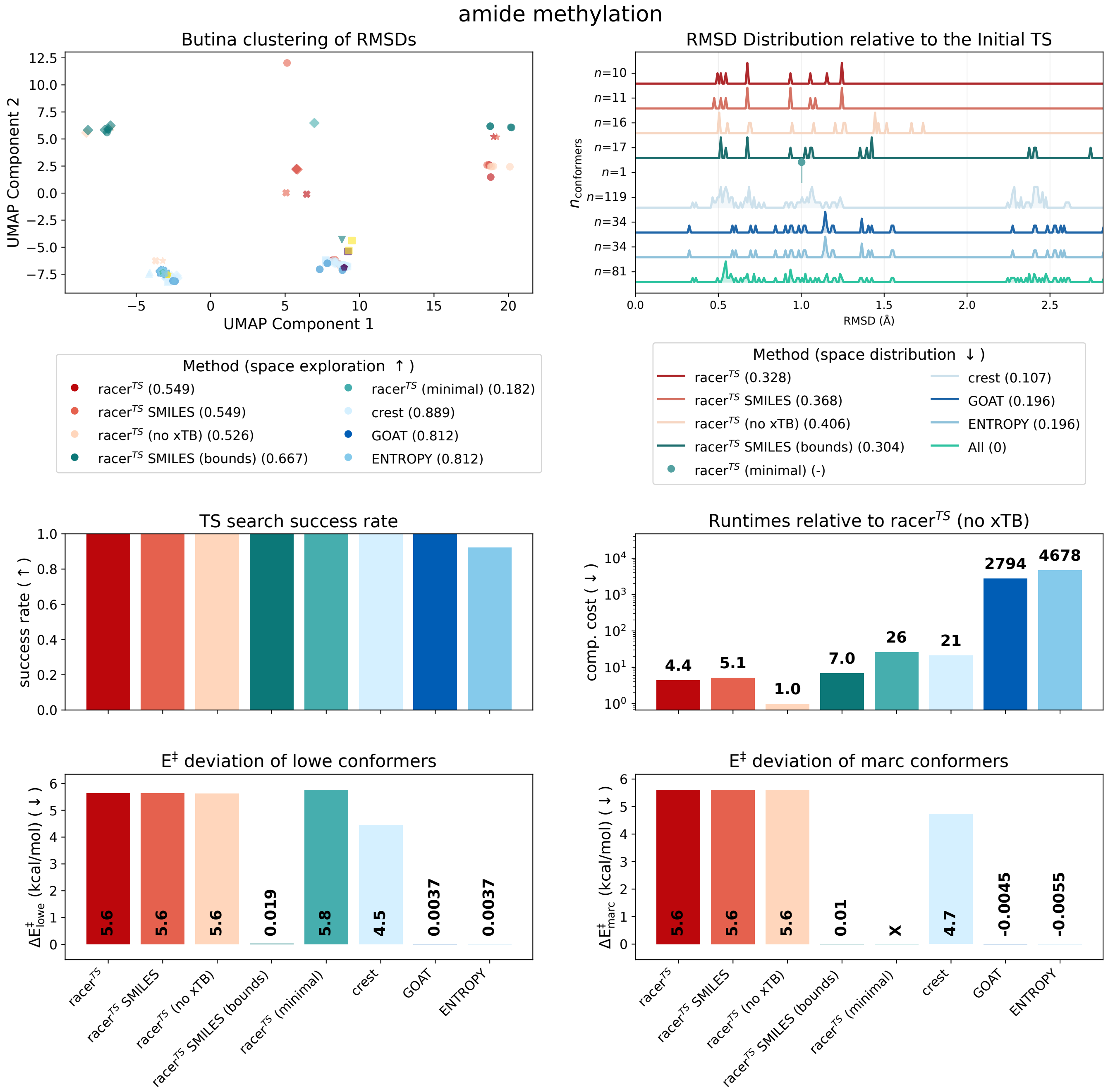

Figure S12: Summary plot of investigated metrics for the amide methylation reaction. **Top left:** UMAP projection and cluster assignment (shape of points) of generated conformers of all methods. The fraction of represented clusters for each method is given in the legend. **Top right:** RMSD distributions w.r.t. the input conformer. JS divergence between the pruned combined distribution and distribution of each method is given in the legend. **Middle left:** Success rate in the DFT pipeline. **Middle right:** Runtime relative to **racer$^{\mathbf{TS}}$ (no xTB)**. **Bottom left:** Deviation of activation energy as calculated using the lowest energy conformer, $\Delta E^{\ddagger}_{\text{lowe}}$. **Bottom right:** Deviation of activation energy as calculated by a marc-selected conformer ensemble $\Delta E^{\ddagger}_{\text{marc}}$.

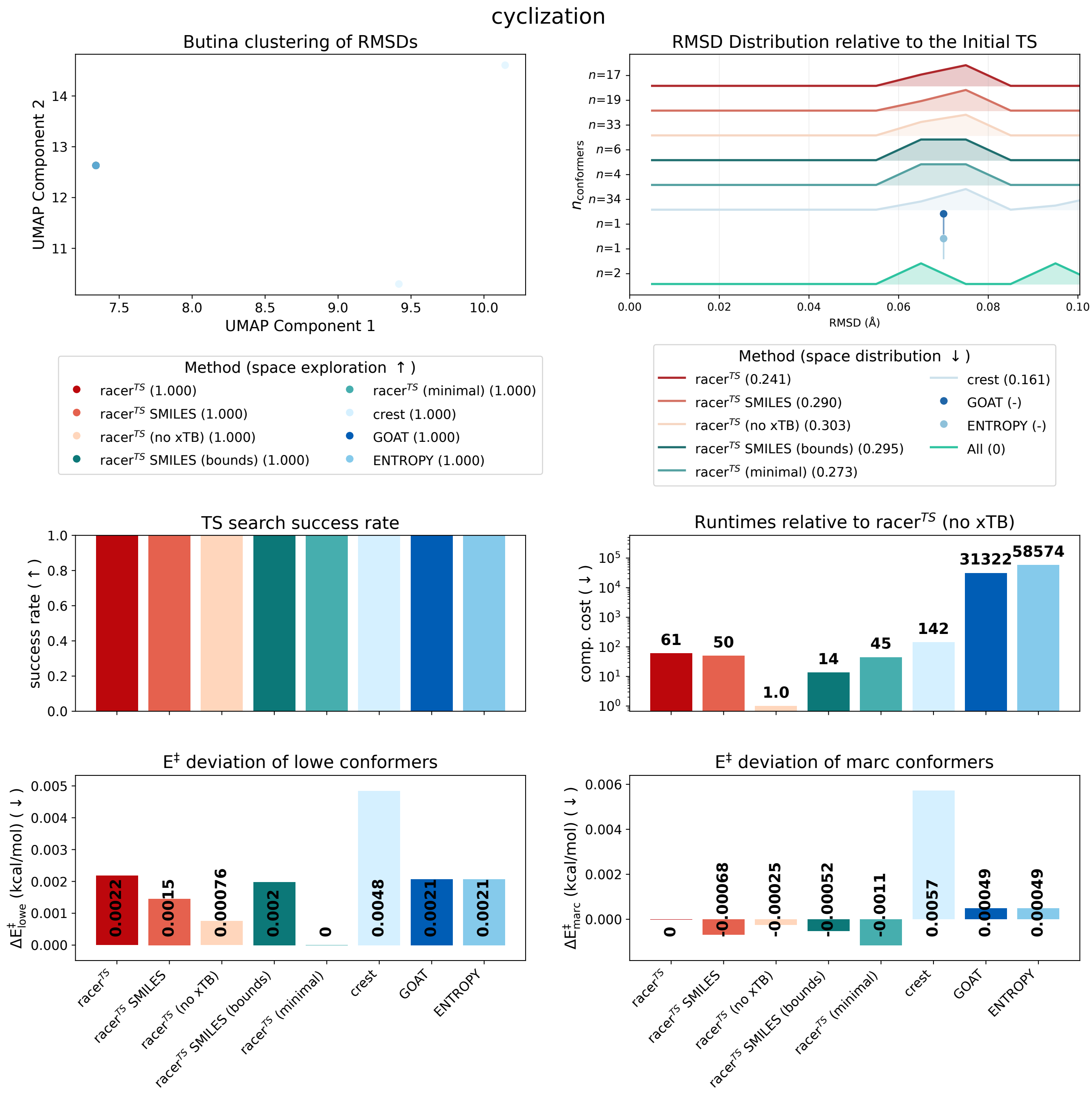

Figure S13: Summary plot of investigated metrics for the cyclization reaction. **Top left:** UMAP projection and cluster assignment (shape of points) of generated conformers of all methods. Fraction of represented clusters for each method is given in the legend. **Top right:** RMSD distributions w.r.t. input conformer. JS divergence between the pruned combined distribution and distribution of each method is given in the legend. **Middle left:** Success rate in the DFT pipeline. **Middle right:** Runtime relative to **racer$^{\mathbf{TS}}$ (no xTB)** . **Bottom left:** Deviation of activation energy as calculated using the lowest energy conformer, $\Delta E^{\ddagger}_{\mathrm{lowe}}$. **Bottom right:** Deviation of activation energy as calculated by a marc-selected conformer ensemble $\Delta E^{\ddagger}_{\mathrm{marc}}$.

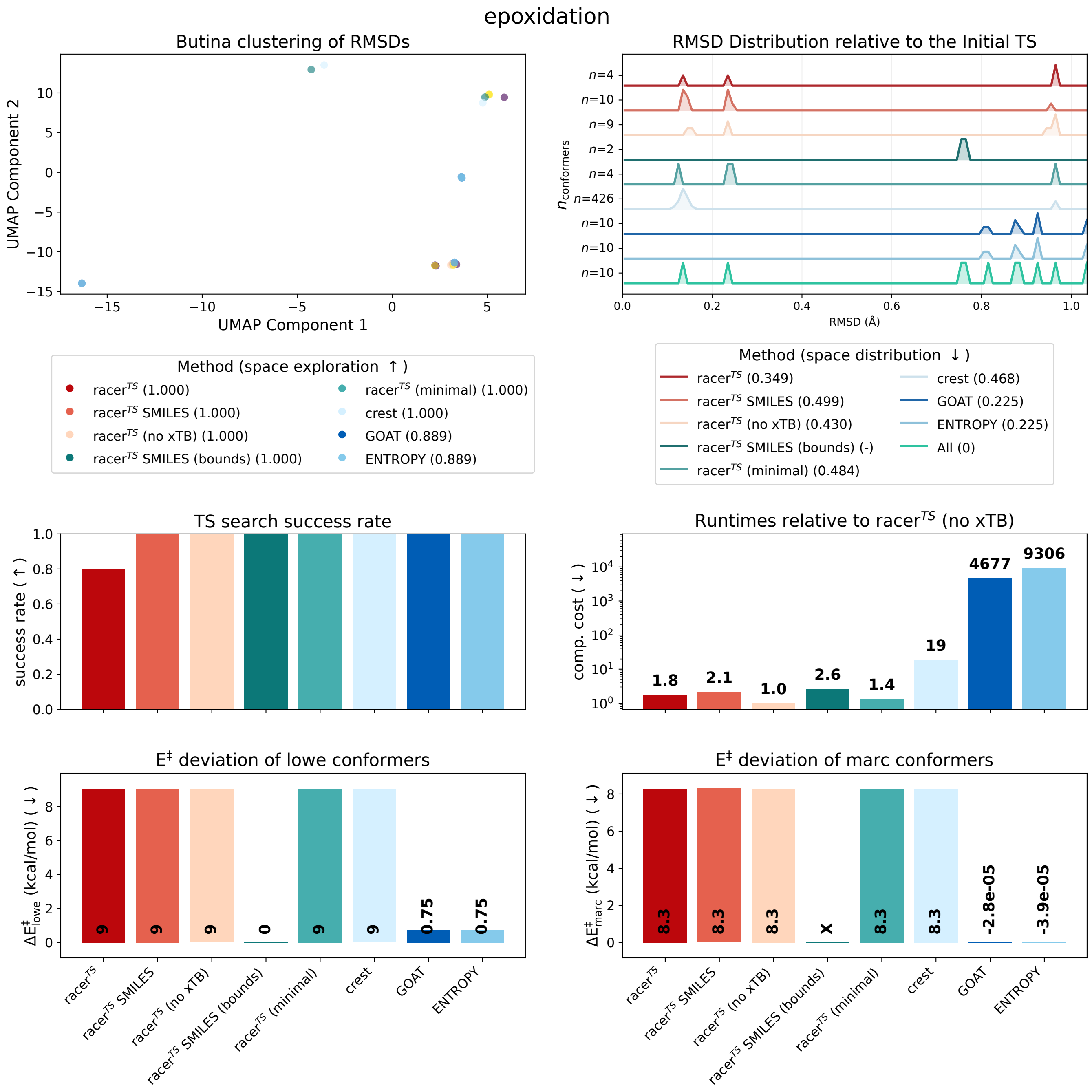

Figure S14: Summary plot of investigated metrics for the epoxidation reaction. **Top left:** UMAP projection and cluster assignment (shape of points) of generated conformers of all methods. Fraction of represented clusters for each method is given in the legend. **Top right:** RMSD distributions w.r.t. input conformer. JS divergence between the pruned combined distribution and distribution of each method is given in the legend. **Middle left:** Success rate in the DFT pipeline. **Middle right:** Runtime relative to **racer$^{\mathbf{TS}}$ (no xTB)**. **Bottom left:** Deviation of activation energy as calculated using the lowest energy conformer, $\Delta E^{\ddagger}_{\mathrm{lowe}}$. **Bottom right:** Deviation of activation energy as calculated by a marc-selected conformer ensemble $\Delta E^{\ddagger}_{\mathrm{marc}}$.

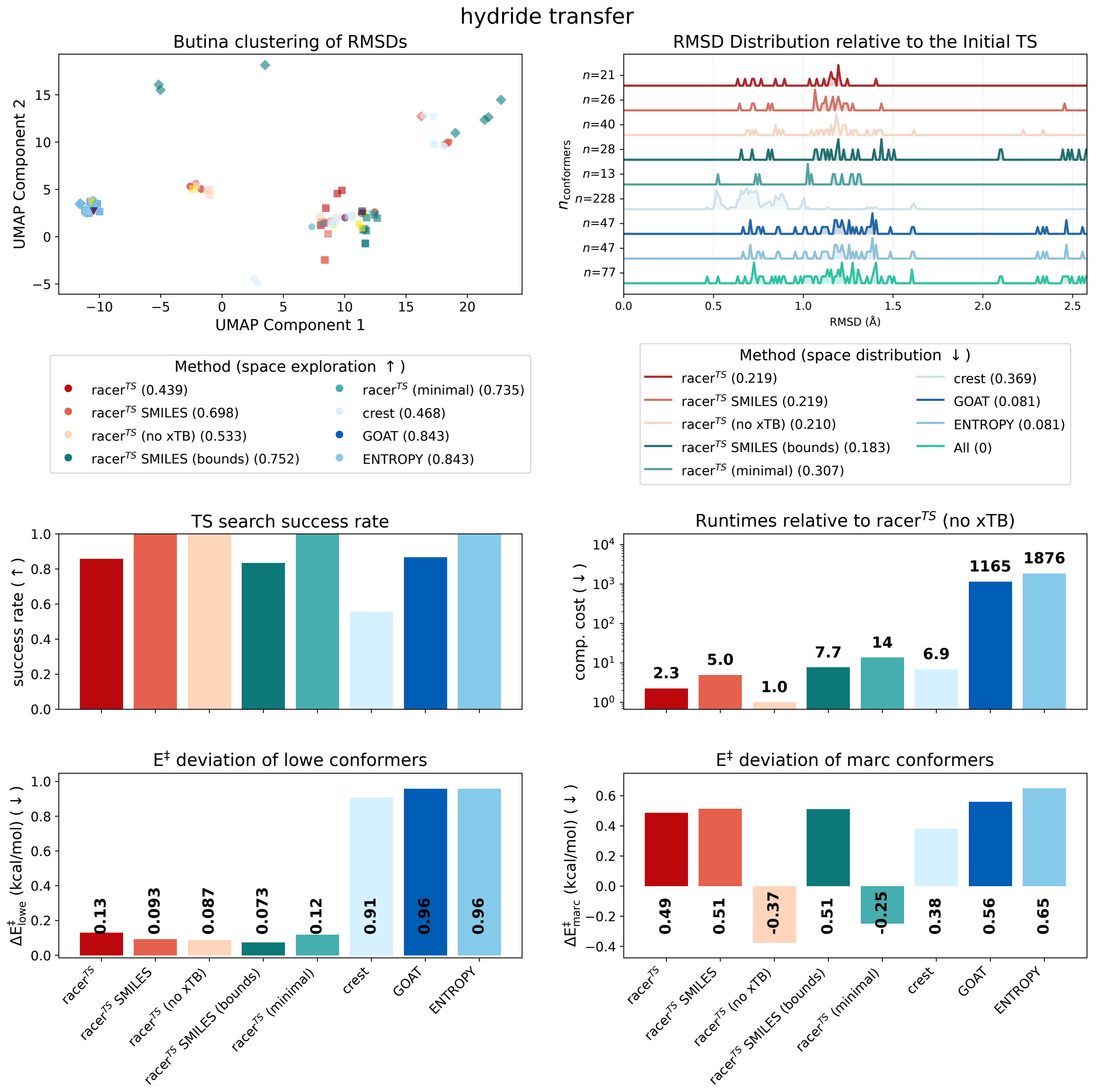

Figure S15: Summary plot of investigated metrics for the hydride transfer reaction. **Top left:** UMAP projection and cluster assignment (shape of points) of generated conformers of all methods. Fraction of represented clusters for each method is given in the legend. **Top right:** RMSD distributions w.r.t. input conformer. JS divergence between the pruned combined distribution and distribution of each method is given in the legend. **Middle left:** Success rate in the DFT pipeline. **Middle right:** Runtime relative to **racer$^{\mathbf{TS}}$ (no xTB)** . **Bottom left:** Deviation of activation energy as calculated using the lowest energy conformer, $\Delta E^{\ddagger}_{\mathrm{lowe}}$. **Bottom right:** Deviation of activation energy as calculated by a marc-selected conformer ensemble $\Delta E^{\ddagger}_{\mathrm{marc}}$.

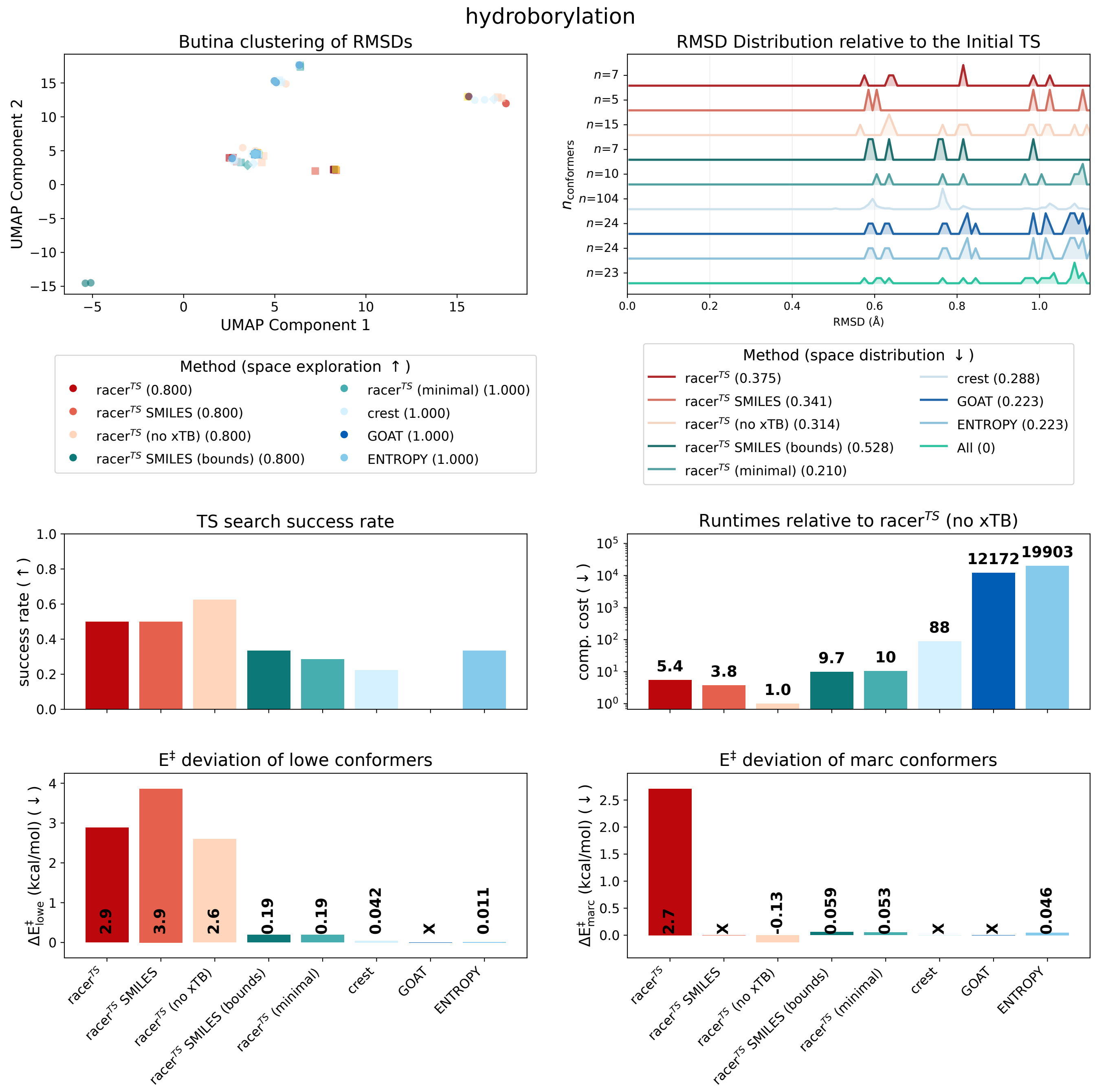

Figure S16: Summary plot of investigated metrics for the hydroborylation reaction. **Top left:** UMAP projection and cluster assignment (shape of points) of generated conformers of all methods. Fraction of represented clusters for each method is given in the legend. **Top right:** RMSD distributions w.r.t. input conformer. JS divergence between the pruned combined distribution and distribution of each method is given in the legend. **Middle left:** Success rate in the DFT pipeline. **Middle right:** Runtime relative to **racer$^{\mathbf{TS}}$ (no xTB)**. **Bottom left:** Deviation of activation energy as calculated using the lowest energy conformer, $\Delta E^{‡}_{\text{lowe}}$. **Bottom right:** Deviation of activation energy as calculated by a marc-selected conformer ensemble $\Delta E^{‡}_{\text{marc}}$.

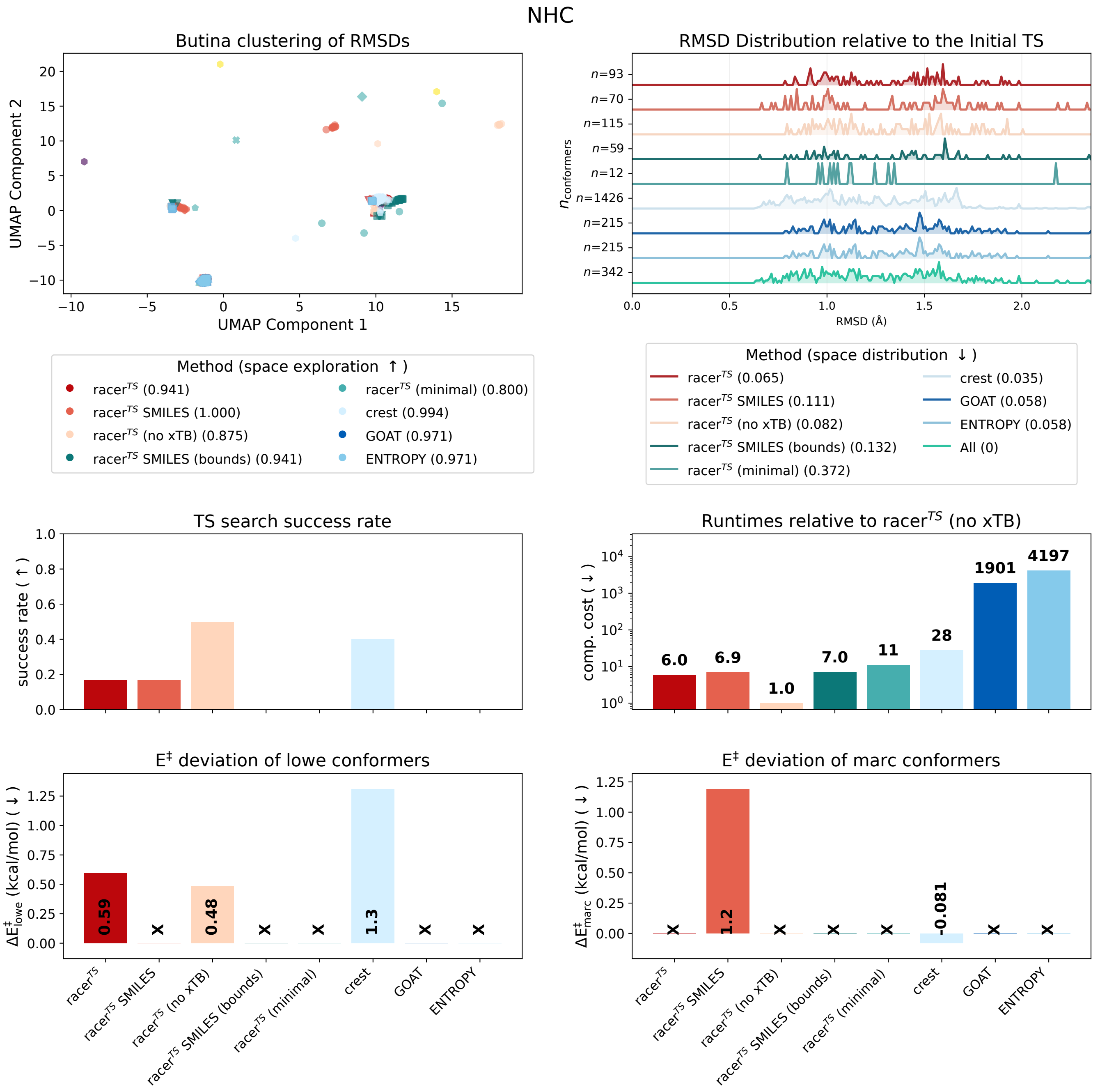

Figure S17: Summary plot of investigated metrics for the NHC reaction. **Top left:** UMAP projection and cluster assignment (shape of points) of generated conformers of all methods. Fraction of represented clusters for each method is given in the legend. **Top right:** RMSD distributions w.r.t. input conformer. JS divergence between the pruned combined distribution and distribution of each method is given in the legend. **Middle left:** Success rate in the DFT pipeline. **Middle right:** Runtime relative to **racer$^{\mathbf{TS}}$ (no xTB)**. **Bottom left:** Deviation of activation energy as calculated using the lowest energy conformer, $\Delta E^{\ddagger}_{\mathrm{lowe}}$. **Bottom right:** Deviation of activation energy as calculated by a marc-selected conformer ensemble $\Delta E^{\ddagger}_{\mathrm{marc}}$.

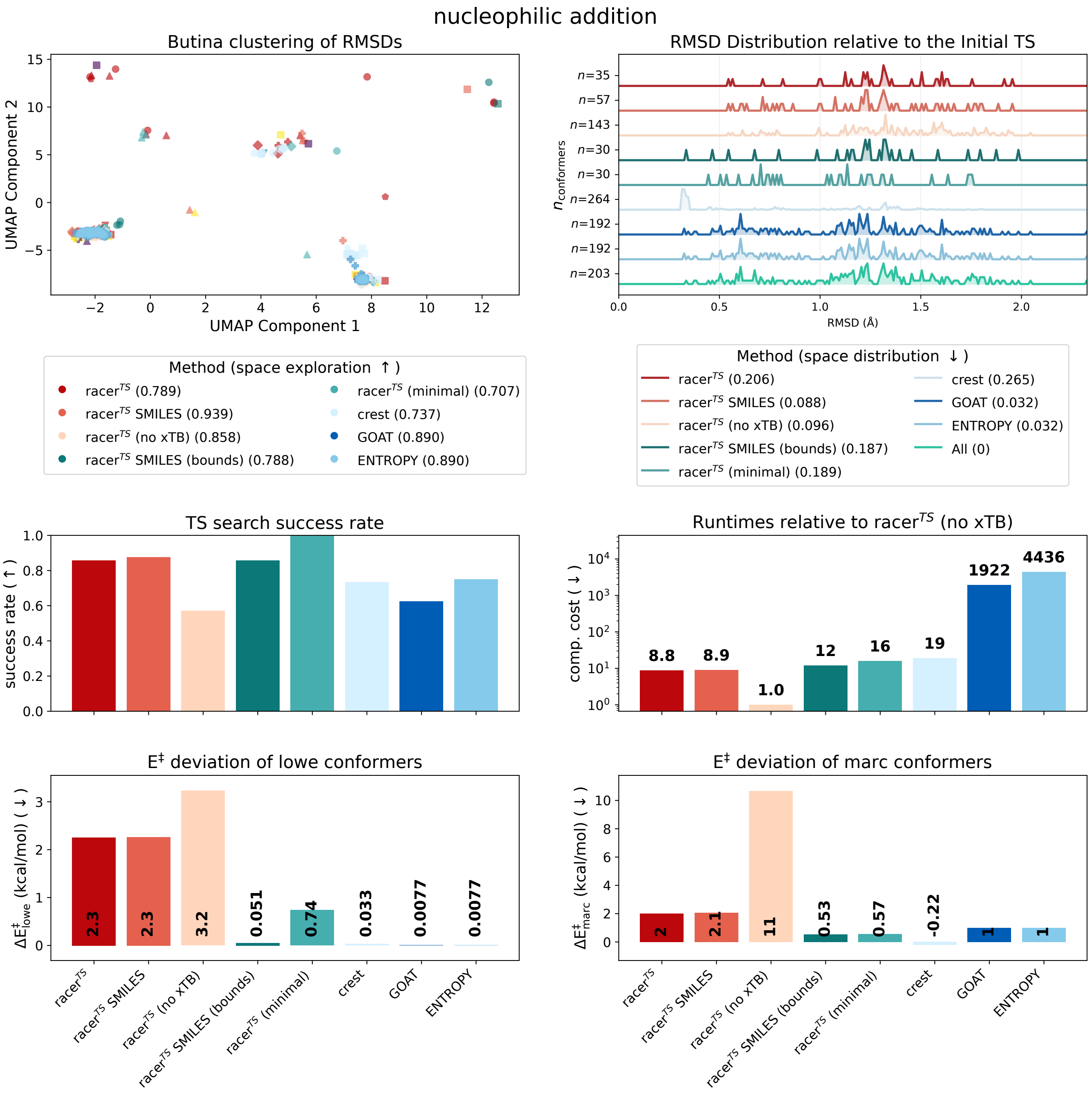

Figure S18: Summary plot of investigated metrics for the nucleophilic addition reaction. **Top left:** UMAP projection and cluster assignment (shape of points) of generated conformers of all methods. Fraction of represented clusters for each method is given in the legend. **Top right:** RMSD distributions w.r.t. input conformer. JS divergence between the pruned combined distribution and distribution of each method is given in the legend. **Middle left:** Success rate in the DFT pipeline. **Middle right:** Runtime relative to **racer^{TS} (no xTB)**. **Bottom left:** Deviation of activation energy as calculated using the lowest energy conformer, $\Delta E^{\ddagger}_{\text{lowe}}$. **Bottom right:** Deviation of activation energy as calculated by a marc-selected conformer ensemble $\Delta E^{\ddagger}_{\text{marc}}$.

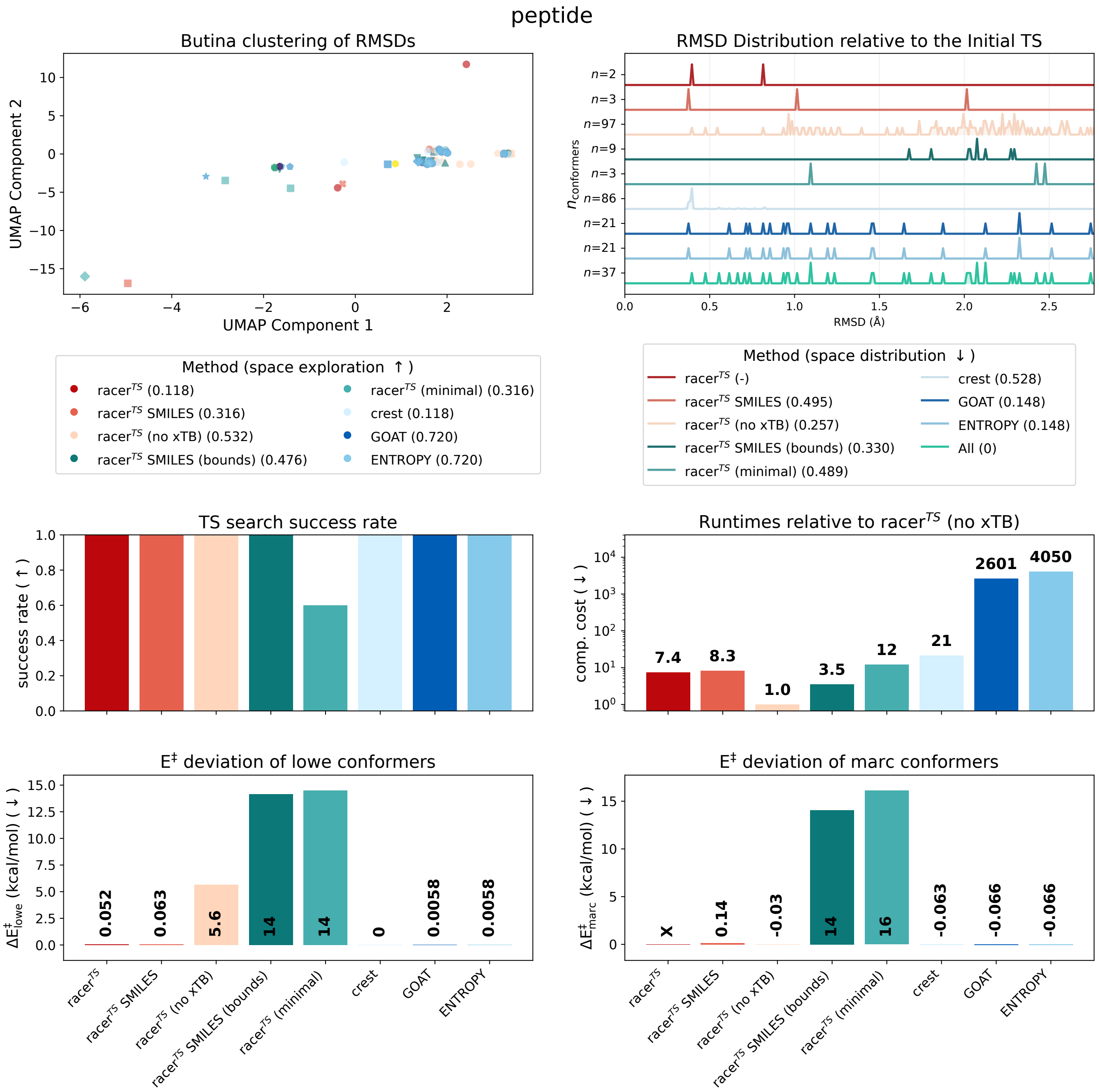

Figure S19: Summary plot of investigated metrics for the peptide reaction. **Top left:** UMAP projection and cluster assignment (shape of points) of generated conformers of all methods. Fraction of represented clusters for each method is given in the legend. **Top right:** RMSD distributions w.r.t. input conformer. JS divergence between the pruned combined distribution and distribution of each method is given in the legend. **Middle left:** Success rate in the DFT pipeline. **Middle right:** Runtime relative to **racer$^{\mathbf{TS}}$ (no xTB)**. **Bottom left:** Deviation of activation energy as calculated using the lowest energy conformer, $\Delta E^{\ddagger}_{\mathrm{lowe}}$. **Bottom right:** Deviation of activation energy as calculated by a marc-selected conformer ensemble $\Delta E^{\ddagger}_{\mathrm{marc}}$.

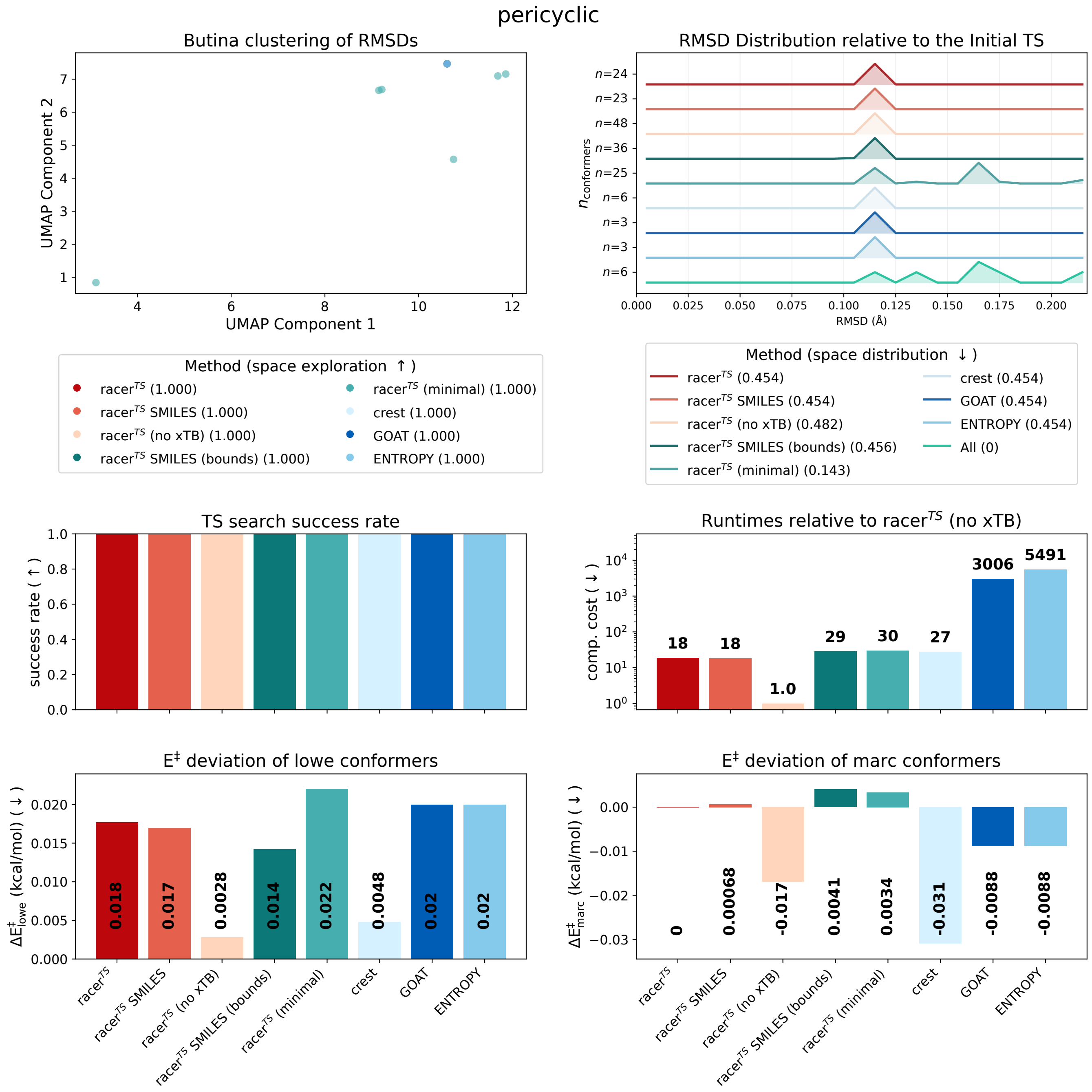

Figure S20: Summary plot of investigated metrics for the pericyclic reaction. **Top left:** UMAP projection and cluster assignment (shape of points) of generated conformers of all methods. Fraction of represented clusters for each method is given in the legend. **Top right:** RMSD distributions w.r.t. input conformer. JS divergence between the pruned combined distribution and distribution of each method is given in the legend. **Middle left:** Success rate in the DFT pipeline. **Middle right:** Runtime relative to **racer^{TS} (no xTB)**. **Bottom left:** Deviation of activation energy as calculated using the lowest energy conformer, $\Delta E^{\ddagger}_{\text{lowe}}$. **Bottom right:** Deviation of activation energy as calculated by a marc-selected conformer ensemble $\Delta E^{\ddagger}_{\text{marc}}$.

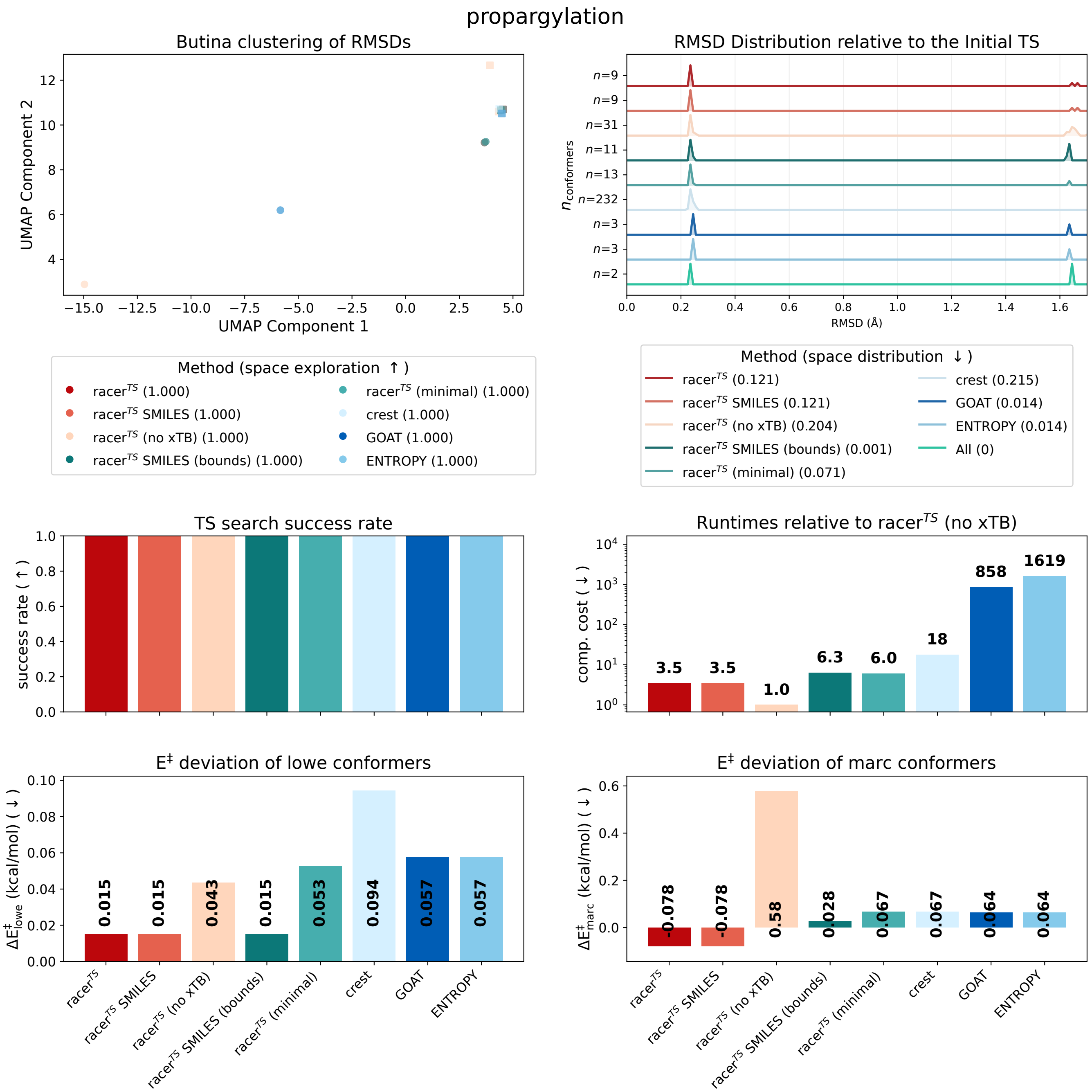

Figure S21: Summary plot of investigated metrics for the propargylation reaction. **Top left:** UMAP projection and cluster assignment (shape of points) of generated conformers of all methods. Fraction of represented clusters for each method is given in the legend. **Top right:** RMSD distributions w.r.t. input conformer. JS divergence between the pruned combined distribution and distribution of each method is given in the legend. **Middle left:** Success rate in the DFT pipeline. **Middle right:** Runtime relative to **racer$^{\mathbf{TS}}$ (no xTB)**. **Bottom left:** Deviation of activation energy as calculated using the lowest energy conformer, $\Delta E^{\ddagger}_{\text{lowe}}$. **Bottom right:** Deviation of activation energy as calculated by a marc-selected conformer ensemble $\Delta E^{\ddagger}_{\text{marc}}$.

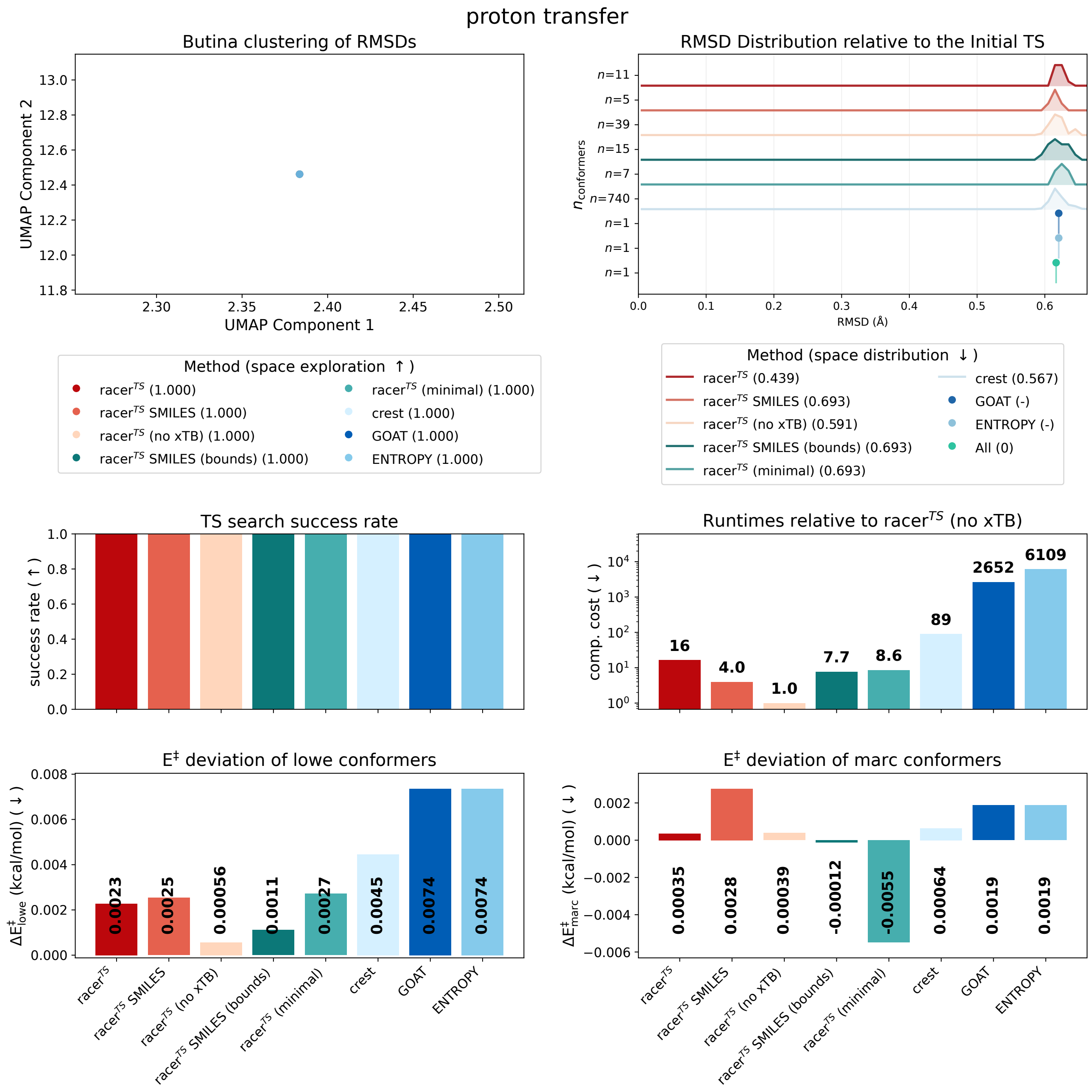

Figure S22: Summary plot of investigated metrics for the proton transfer reaction. **Top left:** UMAP projection and cluster assignment (shape of points) of generated conformers of all methods. Fraction of represented clusters for each method is given in the legend. **Top right:** RMSD distributions w.r.t. input conformer. JS divergence between the pruned combined distribution and distribution of each method is given in the legend. **Middle left:** Success rate in the DFT pipeline. **Middle right:** Runtime relative to **racer$^{\mathbf{TS}}$ (no xTB)**. **Bottom left:** Deviation of activation energy as calculated using the lowest energy conformer, $\Delta E^{\ddagger}_{\mathrm{lowe}}$. **Bottom right:** Deviation of activation energy as calculated by a marc-selected conformer ensemble $\Delta E^{\ddagger}_{\mathrm{marc}}$.

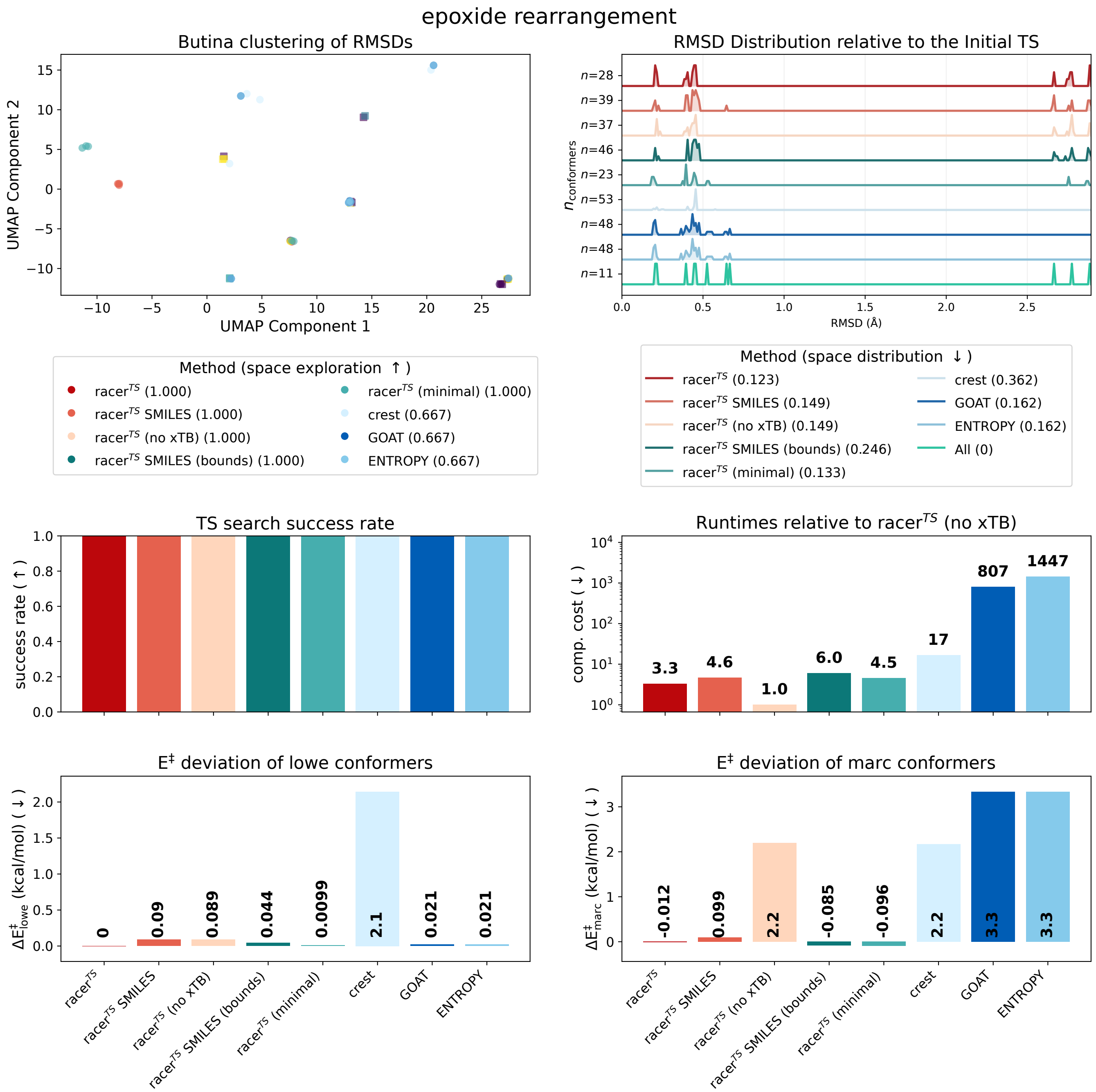

Figure S23: Summary plot of investigated metrics for the epoxide rearrangement reaction. **Top left:** UMAP projection and cluster assignment (shape of points) of generated conformers of all methods. Fraction of represented clusters for each method is given in the legend. **Top right:** RMSD distributions w.r.t. input conformer. JS divergence between the pruned combined distribution and distribution of each method is given in the legend. **Middle left:** Success rate in the DFT pipeline. **Middle right:** Runtime relative to **racer$^{\mathbf{TS}}$ (no xTB)**. **Bottom left:** Deviation of activation energy as calculated using the lowest energy conformer, $\Delta E^{\ddagger}_{\text{lowe}}$. **Bottom right:** Deviation of activation energy as calculated by a marc-selected conformer ensemble $\Delta E^{\ddagger}_{\text{marc}}$.

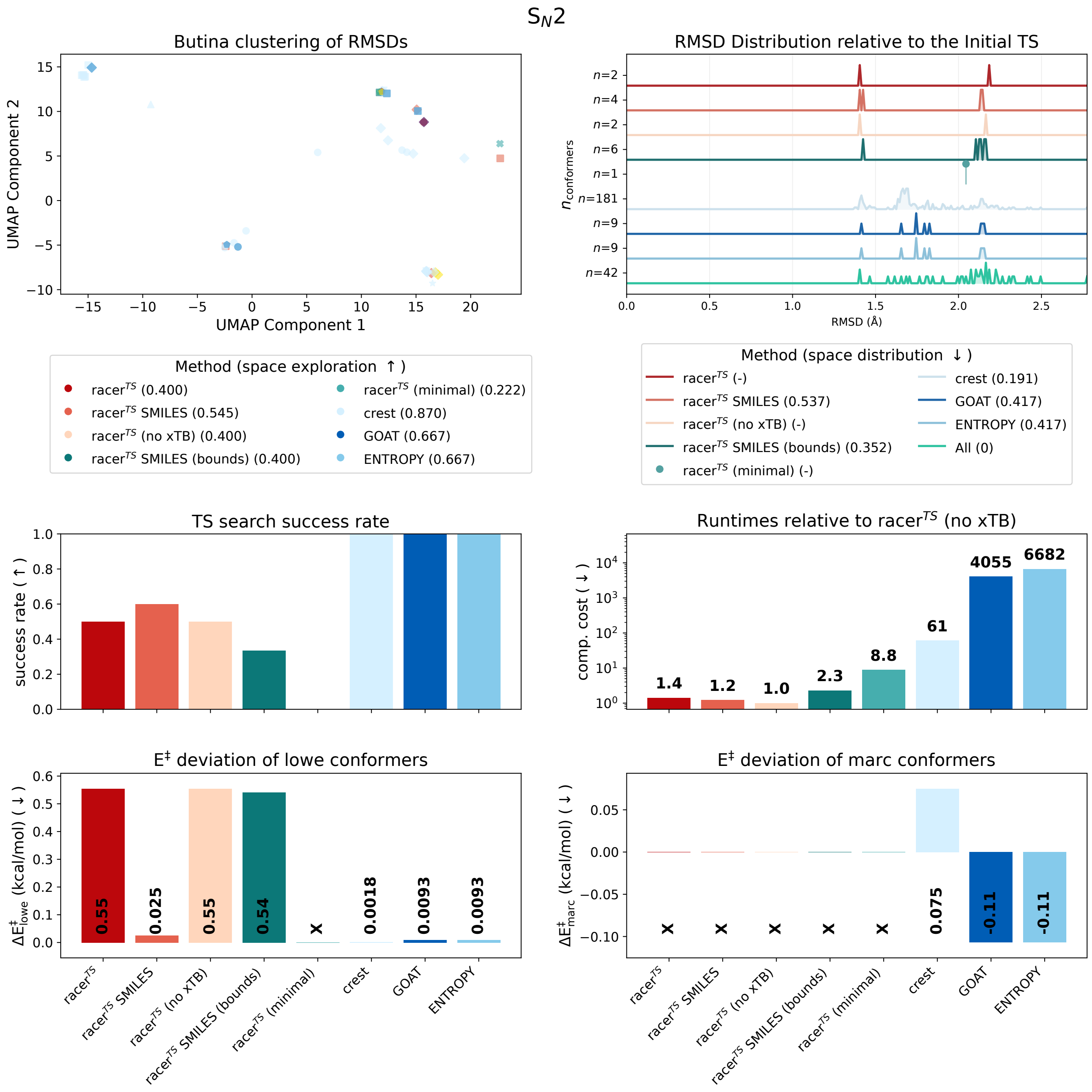

Figure S24: Summary plot of investigated metrics for the S_N2 reaction. **Top left:** UMAP projection and cluster assignment (shape of points) of generated conformers of all methods. Fraction of represented clusters for each method is given in the legend. **Top right:** RMSD distributions w.r.t. input conformer. JS divergence between the pruned combined distribution and distribution of each method is given in the legend. **Middle left:** Success rate in the DFT pipeline. **Middle right:** Runtime relative to **racer$^{\mathbf{TS}}$ (no xTB)**. **Bottom left:** Deviation of activation energy as calculated using the lowest energy conformer, $\Delta E^‡_{\mathrm{lowe}}$. **Bottom right:** Deviation of activation energy as calculated by a marc-selected conformer ensemble $\Delta E^‡_{\mathrm{marc}}$.

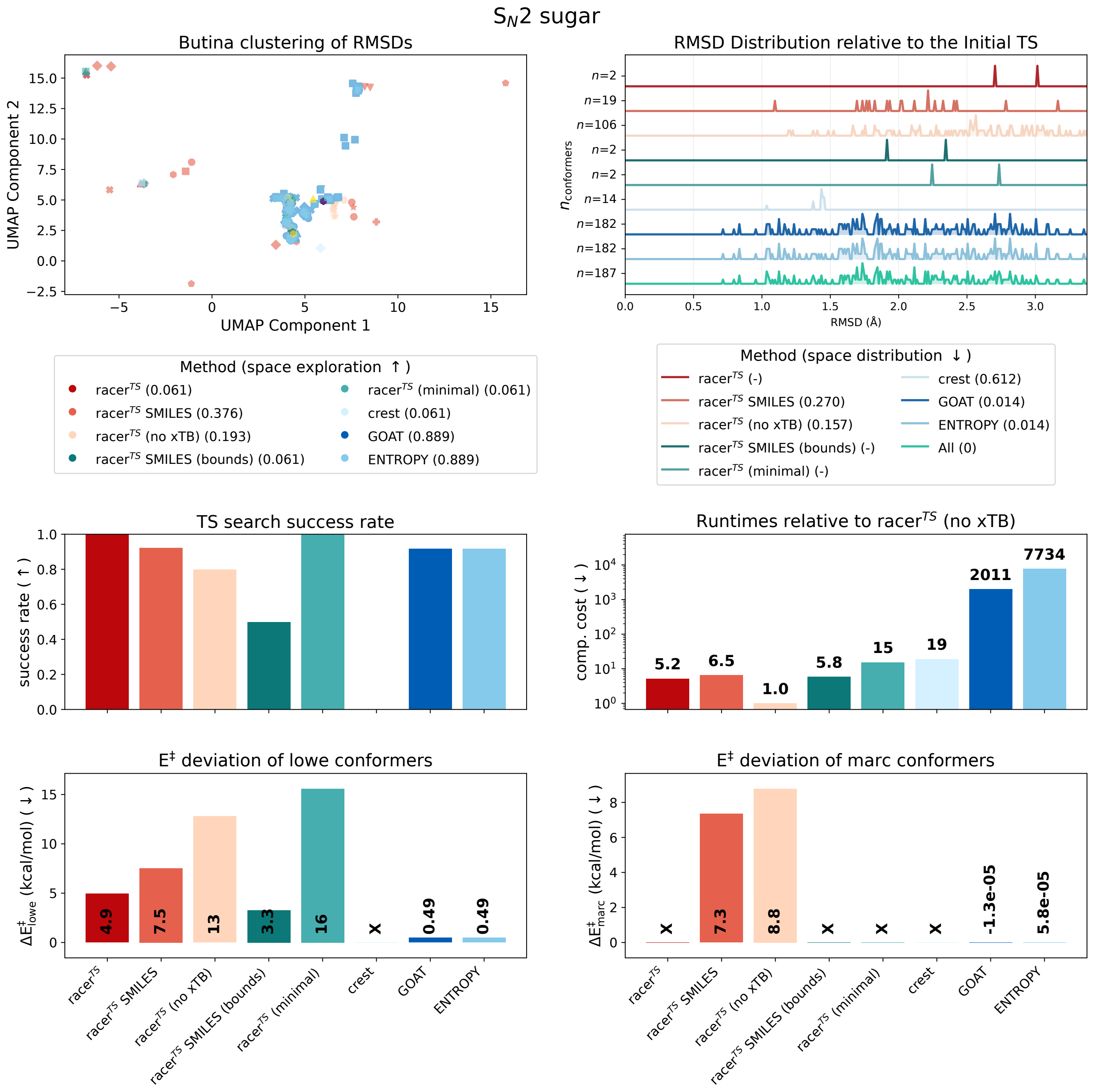

Figure S25: Summary plot of investigated metrics for the S_N2 sugar reaction. **Top left:** UMAP projection and cluster assignment (shape of points) of generated conformers of all methods. Fraction of represented clusters for each method is given in the legend. **Top right:** RMSD distributions w.r.t. input conformer. JS divergence between the pruned combined distribution and distribution of each method is given in the legend. **Middle left:** Success rate in the DFT pipeline. **Middle right:** Runtime relative to **racer$^{\mathbf{TS}}$ (no xTB)**. **Bottom left:** Deviation of activation energy as calculated using the lowest energy conformer, $\Delta E^{\ddagger}_{\mathrm{lowe}}$. **Bottom right:** Deviation of activation energy as calculated by a marc-selected conformer ensemble $\Delta E^{\ddagger}_{\mathrm{marc}}$.

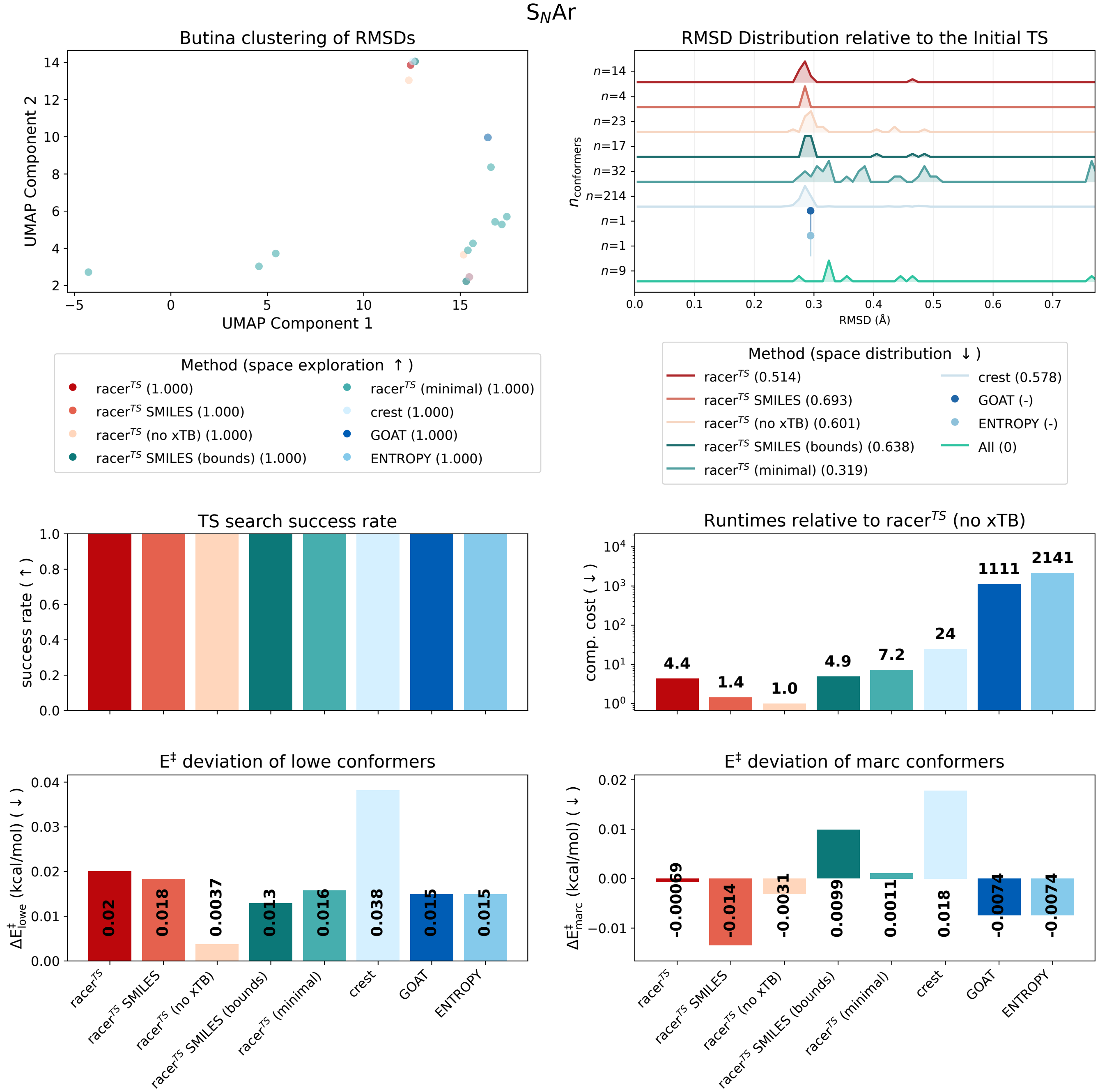

Figure S26: Summary plot of investigated metrics for the S_NAr reaction. **Top left:** UMAP projection and cluster assignment (shape of points) of generated conformers of all methods. Fraction of represented clusters for each method is given in the legend. **Top right:** RMSD distributions w.r.t. input conformer. JS divergence between the pruned combined distribution and distribution of each method is given in the legend. **Middle left:** Success rate in the DFT pipeline. **Middle right:** Runtime relative to **racer$^{\mathbf{TS}}$ (no xTB)**. **Bottom left:** Deviation of activation energy as calculated using the lowest energy conformer, $\Delta E^{\ddagger}_{\mathrm{lowe}}$. **Bottom right:** Deviation of activation energy as calculated by a marc-selected conformer ensemble $\Delta E^{\ddagger}_{\mathrm{marc}}$.

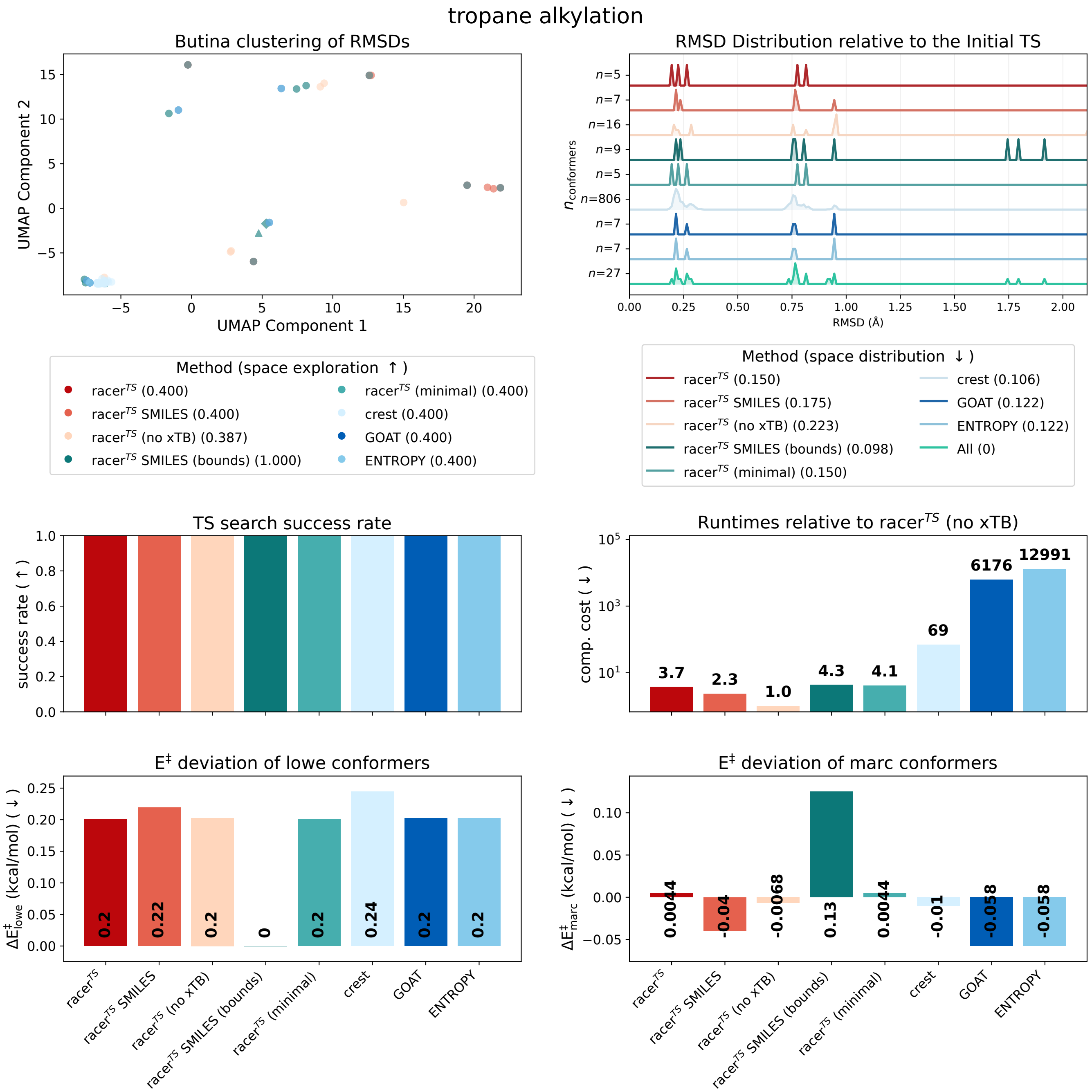

Figure S27: Summary plot of investigated metrics for the tropane alkylation reaction. **Top left:** UMAP projection and cluster assignment (shape of points) of generated conformers of all methods. Fraction of represented clusters for each method is given in the legend. **Top right:** RMSD distributions w.r.t. input conformer. JS divergence between the pruned combined distribution and distribution of each method is given in the legend. **Middle left:** Success rate in the DFT pipeline. **Middle right:** Runtime relative to **racer$^{\mathbf{TS}}$ (no xTB)**. **Bottom left:** Deviation of activation energy as calculated using the lowest energy conformer, $\Delta E^{\ddagger}_{\text{lowe}}$. **Bottom right:** Deviation of activation energy as calculated by a marc-selected conformer ensemble $\Delta E^{\ddagger}_{\text{marc}}$.

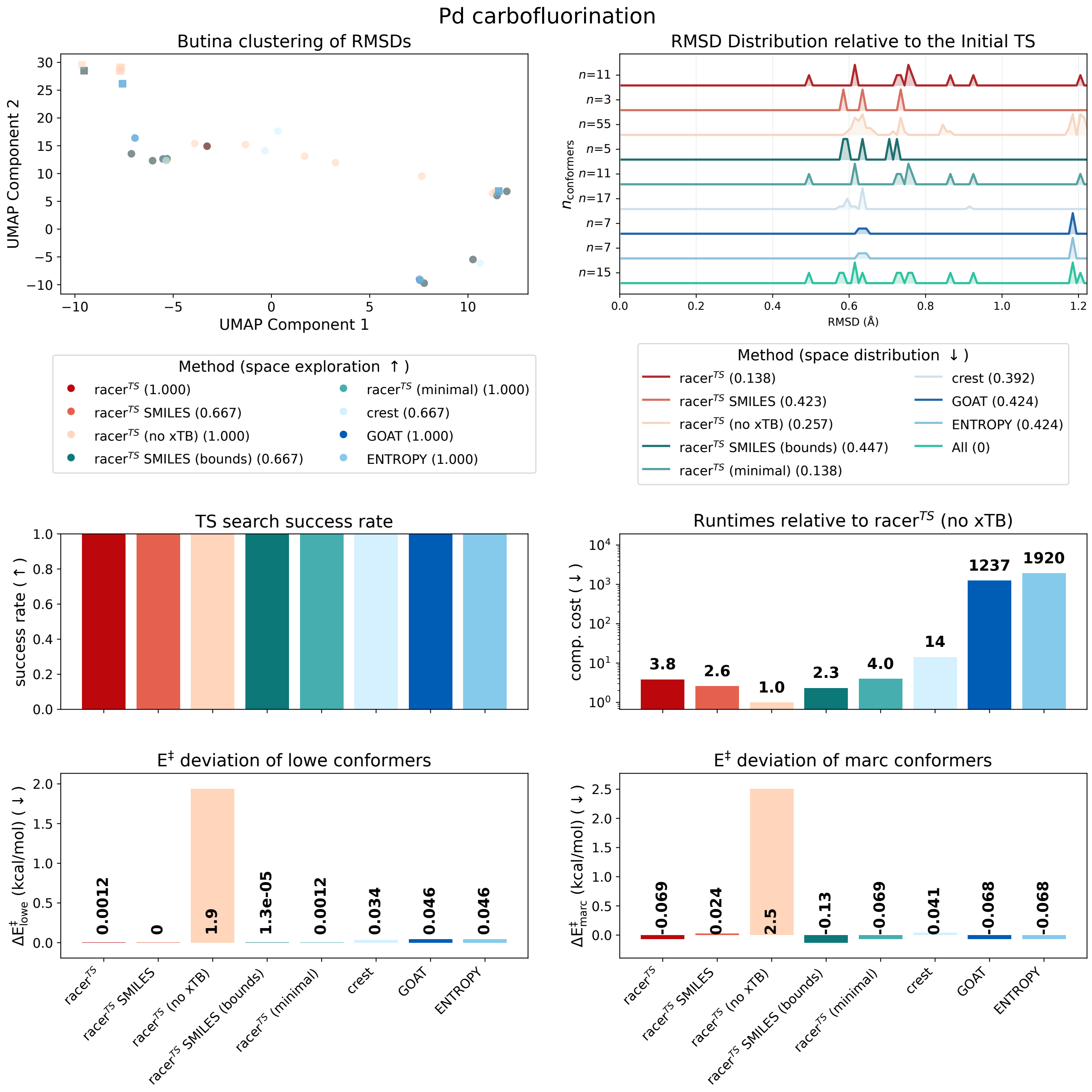

Figure S28: Summary plot of investigated metrics for the Pd carbofluorination reaction. **Top left:** UMAP projection and cluster assignment (shape of points) of generated conformers of all methods. Fraction of represented clusters for each method is given in the legend. **Top right:** RMSD distributions w.r.t. input conformer. JS divergence between the pruned combined distribution and distribution of each method is given in the legend. **Middle left:** Success rate in the DFT pipeline. **Middle right:** Runtime relative to **racer$^{\mathbf{TS}}$ (no xTB)**. **Bottom left:** Deviation of activation energy as calculated using the lowest energy conformer, $\Delta E^{\ddagger}_{\text{lowe}}$. **Bottom right:** Deviation of activation energy as calculated by a marc-selected conformer ensemble $\Delta E^{\ddagger}_{\text{marc}}$.

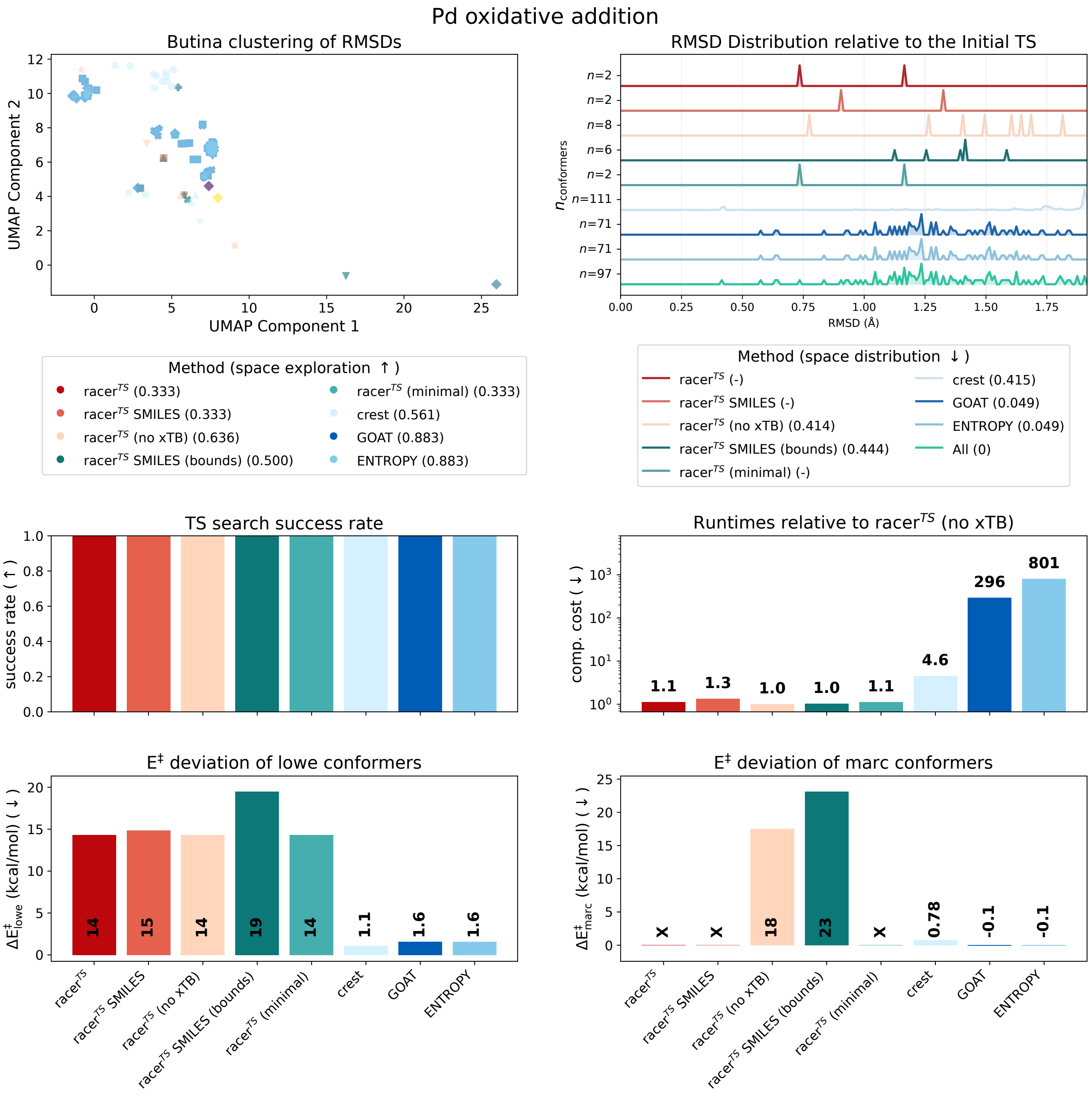

Figure S29: Summary plot of investigated metrics for the Pd oxidative addition reaction. **Top left:** UMAP projection and cluster assignment (shape of points) of generated conformers of all methods. Fraction of represented clusters for each method is given in the legend. **Top right:** RMSD distributions w.r.t. input conformer. JS divergence between the pruned combined distribution and distribution of each method is given in the legend. **Middle left:** Success rate in the DFT pipeline. **Middle right:** Runtime relative to **racer$^{\mathbf{TS}}$ (no xTB)**. **Bottom left:** Deviation of activation energy as calculated using the lowest energy conformer, $\Delta E^{\ddagger}_{\mathrm{lowe}}$. **Bottom right:** Deviation of activation energy as calculated by a marc-selected conformer ensemble $\Delta E^{\ddagger}_{\mathrm{marc}}$.

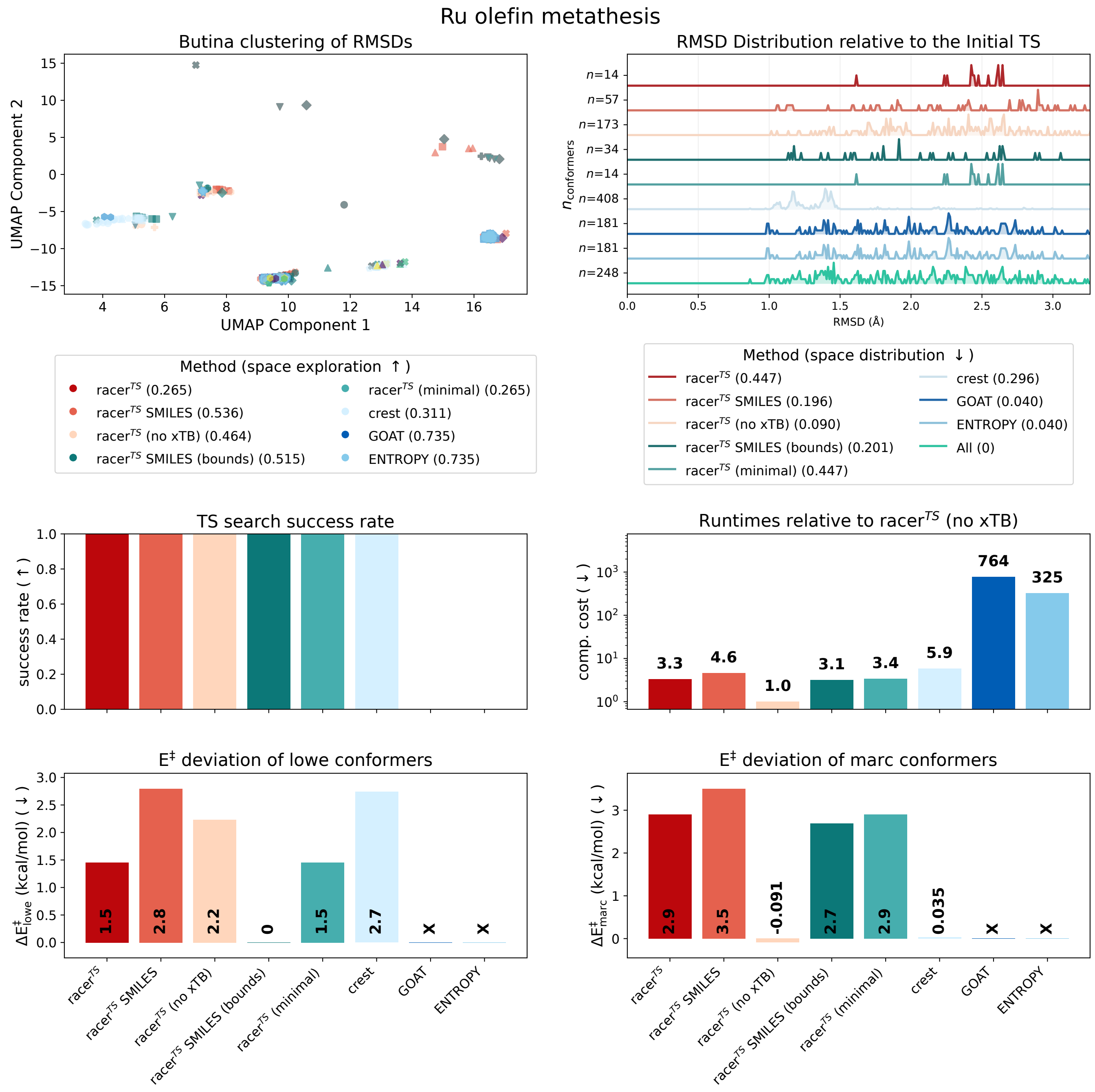

Figure S30: Summary plot of investigated metrics for the Ru olefin metathesis reaction. **Top left:** UMAP projection and cluster assignment (shape of points) of generated conformers of all methods. Fraction of represented clusters for each method is given in the legend. **Top right:** RMSD distributions w.r.t. input conformer. JS divergence between the pruned combined distribution and distribution of each method is given in the legend. **Middle left:** Success rate in the DFT pipeline. **Middle right:** Runtime relative to **racer$^{\mathbf{TS}}$ (no xTB)**. **Bottom left:** Deviation of activation energy as calculated using the lowest energy conformer, $\Delta E^{\ddagger}_{\text{lowe}}$. **Bottom right:** Deviation of activation energy as calculated by a marc-selected conformer ensemble $\Delta E^{\ddagger}_{\text{marc}}$.

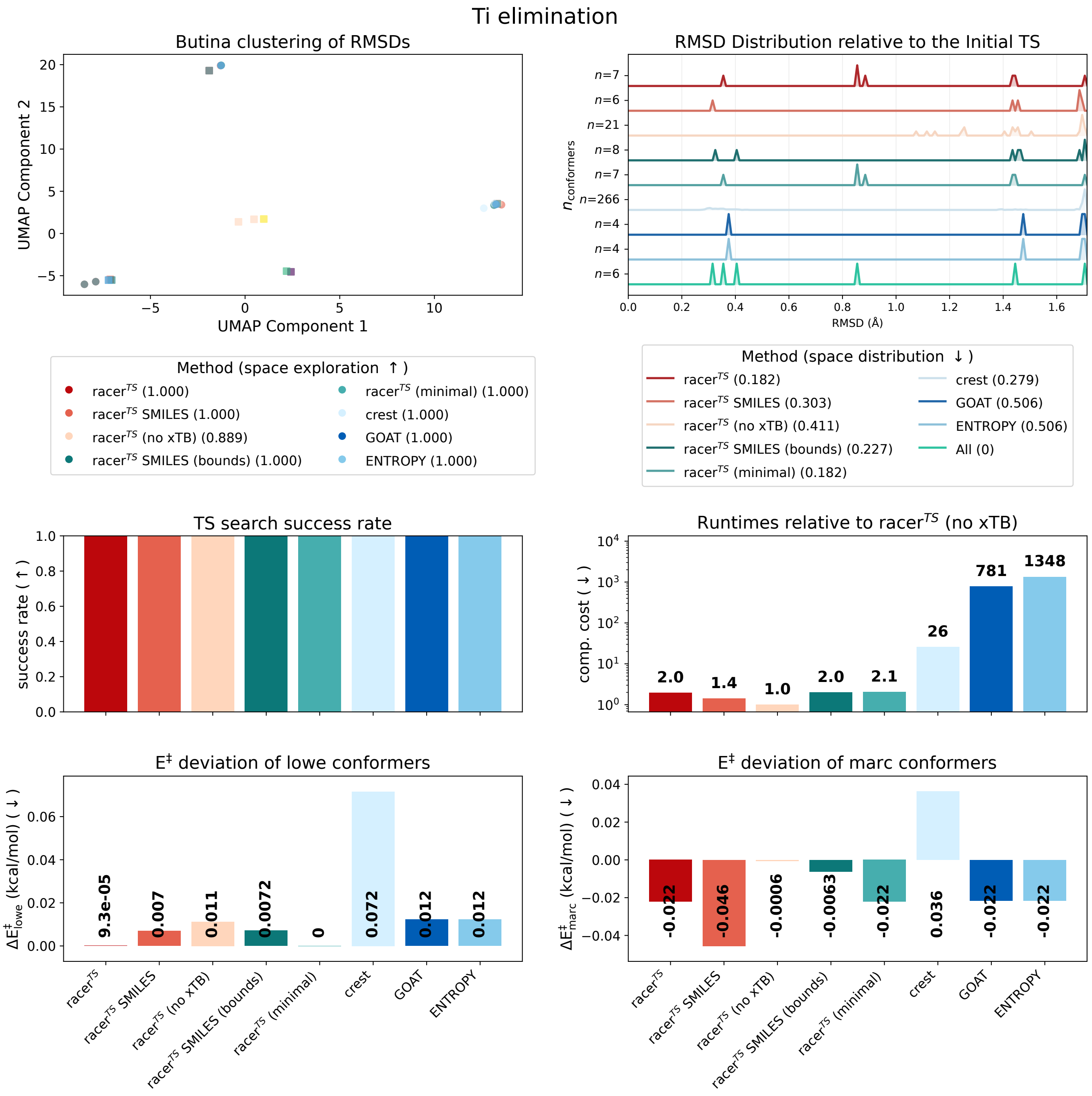

Figure S31: Summary plot of investigated metrics for the Ti elimination reaction. **Top left:** UMAP projection and cluster assignment (shape of points) of generated conformers of all methods. Fraction of represented clusters for each method is given in the legend. **Top right:** RMSD distributions w.r.t. input conformer. JS divergence between the pruned combined distribution and distribution of each method is given in the legend. **Middle left:** Success rate in the DFT pipeline. **Middle right:** Runtime relative to **racer$^{\mathbf{TS}}$ (no xTB)**. **Bottom left:** Deviation of activation energy as calculated using the lowest energy conformer, $\Delta E^{\ddagger}_{\mathrm{lowe}}$. **Bottom right:** Deviation of activation energy as calculated by a marc-selected conformer ensemble $\Delta E^{\ddagger}_{\mathrm{marc}}$.

Supporting Information References

(1) Landrum, G. A. More on constrained embedding – RDKit blog. 2023; https://greglandrum.github.io/rdkit-blog/posts/2023-02-10-more-on-constrained-embedding.html.

(2) Ebejer, J.-P.; Morris, G. M.; Deane, C. M. Freely Available Conformer Generation Methods: How Good Are They? *Journal of Chemical Information and Modeling* **2012**, *52*, 1146–1158.

(3) Landrum, G. A. Understanding conformer generation failures – RDKit blog. 2023; https://greglandrum.github.io/rdkit-blog/posts/2023-05-17-understanding-confgen-errors.html.

(4) Riniker, S.; Landrum, G. A. Better Informed Distance Geometry: Using What We Know To Improve Conformation Generation. *Journal of Chemical Information and Modeling* **2015**, *55*, 2562–2574.

(5) Iribarren, I.; Trujillo, C. Efficiency and Suitability when Exploring the Conformational Space of Phase-Transfer Catalysts. *Journal of Chemical Information and Modeling* **2022**, *62*, 5568–5580.

(6) McNutt, A. T.; Bisiriyu, F.; Song, S.; Vyas, A.; Hutchison, G. R.; Koes, D. R. Conformer Generation for Structure-Based Drug Design: How Many and How Good? *Journal of Chemical Information and Modeling* **2023**, *63*, 6598–6607.

(7) Halgren, T. A. Merck molecular force field. I. Basis, form, scope, parameterization, and performance of MMFF94. *Journal of Computational Chemistry* **1996**, *17*, 490–519.

(8) Rappe, A. K.; Casewit, C. J.; Colwell, K. S.; Goddard, W. A. I.; Skiff, W. M. UFF, a full periodic table force field for molecular mechanics and molecular dynamics simulations. *Journal of the American Chemical Society* **1992**, *114*, 10024–10035.

(9) Grimme, S.; Hansen, A.; Ehlert, S.; Mewes, J.-M. r2SCAN-3c: A "Swiss army knife" composite electronic-structure method. *The Journal of Chemical Physics* **2021**, *154*, 064103.

(10) Goodman, J. M.; Silva, M. A. QRC: a rapid method for connecting transition structures to reactants in the computational analysis of organic reactivity. *Tetrahedron Letters* **2003**, *44*, 8233–8236.

(11) Silva, M. A.; Goodman, J. M. Aziridinium ring opening: a simple ionic reaction pathway with sequential transition states. *Tetrahedron Letters* **2005**, *46*, 2067–2069.

(12) Qin, H.; Han, Z.; Bonku, E. M.; Sun, H.; Odilov, A.; Zhu, F.; Abduahadi, S.; Zhu, W.; Shen, J.; Aisa, H. A. Direct esterification of amides by the dimethylsulfate-mediated activation of amide C–N bonds. *Communications Chemistry* **2024**, *7*, 1–9.

(13) Hanaway, D. J.; Kennedy, C. R. Automated Variable Electric-Field DFT Application for Evaluation of Optimally Oriented Electric Fields on Chemical Reactivity. *The Journal of Organic Chemistry* **2023**, *88*, 106–115.

(14) Schneebeli, S. T.; Hall, M. L.; Breslow, R.; Friesner, R. Quantitative DFT Modeling of the Enantiomeric Excess for Dioxirane-Catalyzed Epoxidations. *Journal of the American Chemical Society* **2009**, *131*, 3965–3973.

(15) Prasad, V. K.; Pei, Z.; Edelmann, S.; Otero-de-la Roza, A.; DiLabio, G. A. BH9, a New Comprehensive Benchmark Data Set for Barrier Heights and Reaction Energies: Assessment of Density Functional Approximations and Basis Set Incompleteness Potentials. *Journal of Chemical Theory and Computation* **2022**, *18*, 151–166.

(16) Li, Y.; Kang, Y.; Xiao, J.; Zhang, Z. Mechanism, Chemoselectivity, and Stereoselectivity of an NHC-Catalyzed Reaction of Aldehydes and Hydrazones: A DFT Study. *The Journal of Physical Chemistry A* **2024**, *128*, 4483–4492.

(17) Zhao, Q. YARP reaction database. 2021; https://figshare.com/articles/dataset/YARP/14766624/7.

(18) Zhao, Q.; Savoie, B. M. Simultaneously improving reaction coverage and computational cost in automated reaction prediction tasks. *Nature Computational Science* **2021**, *1*, 479–490.

(19) Doney, A. C.; Rooks, B. J.; Lu, T.; Wheeler, S. E. Design of Organocatalysts for Asymmetric Propargylations through Computational Screening. *ACS Catalysis* **2016**, *6*, 7948–7955.

(20) Nakajima, M.; Adachi, Y.; Nemoto, T. Computation-guided asymmetric total syntheses of resveratrol dimers. *Nature Communications* **2022**, *13*, 152.

(21) Yan, M.; Zhang, Z.; Zhou, J.; Li, W.; Zhang, C.; Fan, S.; Yang, Z. DFT calculation and NMR data of novel aryloxymaleimides and the intermediates and transition states in the reaction. *Data in Brief* **2019**, *25*, 104110.

(22) Oloba-Whenu, O. A.; Isanbor, C. DFT Study of the SNAr Reactions of 1-Chloro-2-nitro- and 1-Phenoxy-2-nitro-benzenes with Aniline in Acetonitrile and Toluene: Concerted or Multistep Mechanism? *ChemistrySelect* **2023**, *8*, e202204480.

(23) Laplaza, R.; Wodrich, M. D.; Corminboeuf, C. Overcoming the Pitfalls of Computing Reaction Selectivity from Ensembles of Transition States. *The Journal of Physical Chemistry Letters* **2024**, *15*, 7363–7370.

(24) Yang, G.; Wu, H.; Gallarati, S.; Corminboeuf, C.; Wang, Q.; Zhu, J. Migrative Carbofluorination of Saturated Amides Enabled by Pd-Based Dyotropic Rearrangement. *Journal of the American Chemical Society* **2022**, *144*, 14047–14052.

(25) Lu, J.; Donnecke, S.; Paci, I.; Leitch, D. C. A reactivity model for oxidative addition to

palladium enables quantitative predictions for catalytic cross-coupling reactions. *Chemical Science* **2022**, *13*, 3477–3488.

(26) Martínez, J. P.; Trzaskowski, B. Olefin Metathesis Catalyzed by a Hoveyda–Grubbs-like Complex Chelated to Bis(2-mercaptoimidazolyl) Methane: A Predictive DFT Study. *The Journal of Physical Chemistry A* **2022**, *126*, 720–732.

(27) Guo, J.; Deng, X.; Song, C.; Lu, Y.; Qu, S.; Dang, Y.; Wang, Z.-X. Differences between the elimination of early and late transition metals: DFT mechanistic insights into the titanium-catalyzed synthesis of pyrroles from alkynes and diazenes. *Chemical Science* **2017**, *8*, 2413–2425.

(28) Spicher, S.; Grimme, S. Robust Atomistic Modeling of Materials, Organometallic, and Biochemical Systems. *Angewandte Chemie International Edition* **2020**, *59*, 15665–15673.

(29) Bannwarth, C.; Ehlert, S.; Grimme, S. GFN2-xTB—An Accurate and Broadly Parametrized Self-Consistent Tight-Binding Quantum Chemical Method with Multipole Electrostatics and Density-Dependent Dispersion Contributions. *Journal of Chemical Theory and Computation* **2019**, *15*, 1652–1671.

(30) Neese, F. The ORCA program system. *WIREs Computational Molecular Science* **2012**, *2*, 73–78.

(31) Neese, F. Software update: The ORCA program system—Version 5.0. *WIREs Computational Molecular Science* **2022**, *12*, e1606.

(32) Bhoorasingh, P. L.; Slakman, B. L.; Seyedzadeh Khanshan, F.; Cain, J. Y.; West, R. H. Automated Transition State Theory Calculations for High-Throughput Kinetics. *The Journal of Physical Chemistry A* **2017**, *121*, 6896–6904.

(33) Grambow, C. A.; Jamal, A.; Li, Y.-P.; Green, W. H.; Zádor, J.; Suleimanov, Y. V. Unimolecular Reaction Pathways of a -Ketohydroperoxide from Combined Application of Automated Reaction Discovery Methods. *Journal of the American Chemical Society* **2018**, *140*, 1035–1048.

(34) Friederich, P.; Gomes, G. d. P.; Bin, R. D.; Aspuru-Guzik, A.; Balcells, D. Machine learning dihydrogen activation in the chemical space surrounding Vaska's complex. *Chemical Science* **2020**, *11*, 4584–4601.

(35) Pattanaik, L.; Ingraham, J. B.; Grambow, C. A.; Green, W. H. Generating transition states of isomerization reactions with deep learning. *Physical Chemistry Chemical Physics* **2020**, *22*, 23618–23626.

(36) Nigam, A.; Pollice, R.; Tom, G.; Jorner, K.; Willes, J.; Thiede, L.; Kundaje, A.; Aspuru-Guzik, A. Tartarus: A Benchmarking Platform for Realistic And Practical Inverse Molecular Design. *Advances in Neural Information Processing Systems* **2023**, *36*, 3263–3306.

(37) Froitzheim, T.; Müller, M.; Hansen, A.; Grimme, S. g-xTB: A General-Purpose Extended Tight-Binding Electronic Structure Method For the Elements H to Lr (Z=1–103). 2025; https://chemrxiv.org/engage/chemrxiv/article-details/685434533ba0887c335fc974.

(38) Wood, B. M. et al. UMA: A Family of Universal Models for Atoms. 2025; http://arxiv.org/abs/2506.23971.
